# Synthesis of an arrayed sgRNA library targeting the human genome

**DOI:** 10.1038/srep14987

**Published:** 2015-10-08

**Authors:** Tobias Schmidt, Jonathan L. Schmid-Burgk, Veit Hornung

**Affiliations:** 1 Institute of Molecular Medicine, University Hospital, University of Bonn, Sigmund-Freud-Str. 25, 53127 Bonn, Germany

## Abstract

Clustered regularly interspaced short palindromic repeats (CRISPR) in conjunction with CRISPR-associated proteins (Cas) can be employed to introduce double stand breaks into mammalian genomes at user-defined loci. The endonuclease activity of the Cas complex can be targeted to a specific genomic region using a single guide RNA (sgRNA). We developed a ligation-independent cloning (LIC) assembly method for efficient and bias-free generation of large sgRNA libraries. Using this system, we performed an iterative shotgun cloning approach to generate an arrayed sgRNA library that targets one critical exon of almost every protein-coding human gene. An orthogonal mixing and deconvolution approach was used to obtain 19,506 unique sequence-validated sgRNAs (91.4% coverage). As tested in HEK 293T cells, constructs of this library have a median genome editing activity of 54.6% and employing sgRNAs of this library to generate knockout cells was successful for 19 out of 19 genes tested.

Clustered regularly interspaced short palindromic repeats (CRISPR) in conjunction with CRISPR-associated proteins (Cas) provide an adaptive immune system to bacteria and archaea targeting foreign genetic material^1^,^2^. Cas9, a member of the type II CRISPR-Cas system, requires RNA molecules to be directed as a sequence-specific endonuclease to its target sequence^3^. The native crRNA:tracrRNA complex can be mimicked by a chimeric, single guide RNA molecule (sgRNA), in which the invariant base pairing region is replaced by an intramolecular stem loop structure^3^. The target-specific protospacer region of the sgRNA usually consists of 20 nucleotides, in which the highest stringency in base pairing with a target site is demanded within the PAM adjacent region^3^. The CRISPR/Cas9 system can be reprogrammed in a user-defined fashion to induce double-strand breaks (DSBs) in prokaryotic^3^,^4^ and eukaryotic genomes^5^,^6^,^7^,^8^,^9^,^10^.

To reveal novel components in biological pathways, the CRISPR/Cas9 system has recently been used for polyclonal forward genetic screens, in which CRISPR libraries were stably transduced followed by a phenotypical selection step^11^,^12^,^13^. Deep sequencing then allowed the identification of sgRNA sequences that were enriched in selected populations. In these studies PCR- and Gibson-assembly-based approaches were employed to create large polyclonal pools of sgRNA vectors from chip-synthesized oligonucleotides covering the entire human or mouse transcriptome.

Besides polyclonal screening efforts, CIRPSR/Cas9 constitutes a promising tool for the fast and efficient generation of large numbers of knockout cells, e.g. comprising targets from entire pathways. To this end, arrayed sgRNA libraries encompassing the entire protein coding genome will constitute a powerful resource. Since the synthesis of sgRNAs using individual oligonucleotides is laborious and costly, the approach of generating arrayed libraries from chip-synthesized oligo pools is currently the most attractive choice. To this end, a method would be desirable that allows singling out clonal sgRNA constructs from polyclonal source material with minimal sequence bias. At the same time, an efficient sequence validation method is needed to allocate individual sgRNA clones in an arrayed library. To address these challenges, we developed an amplification-free LIC-based library assembly method combined with an orthogonal deep sequencing validation strategy to facilitate the synthesis of large, arrayed sgRNA libraries from polyclonal oligonucleotide pools. In four iterative rounds of shotgun assembly and sequencing we were able to generate an arrayed genome-spanning library comprising clonal sgRNAs for targeting most genes in the human genome.

## Results

### Ligation-independent assembly of sgRNA vectors

**Figure 1:**
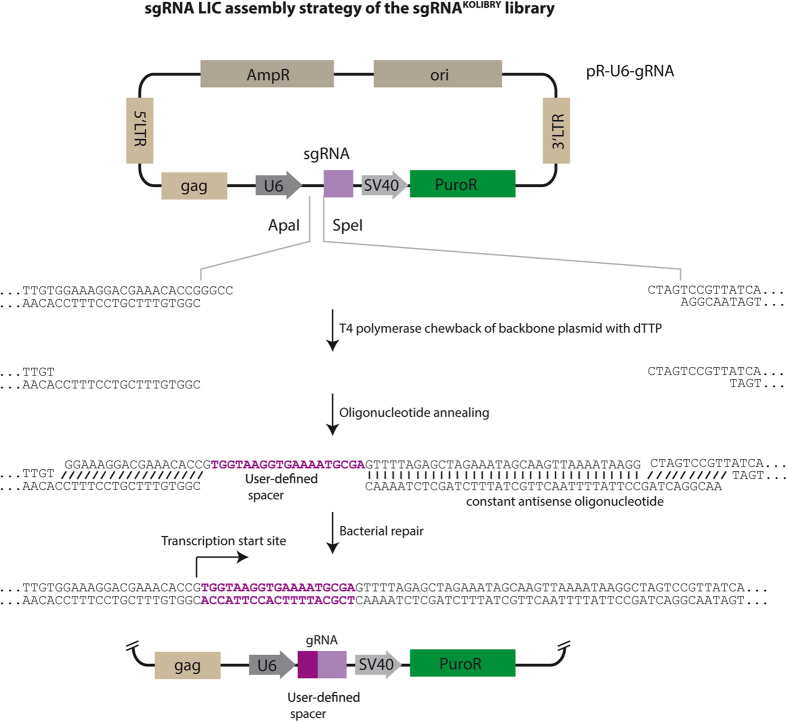
LIC based assembly strategy. A schematic view of the LIC-based sgRNA cloning system is depicted. In brief, a backbone plasmid containing constant portions of a hybrid sgRNA downstream the U6 promoter is first linearized using ApaI and SpeI restriction enzymes. Subsequently, a T4 DNA polymerase chewback reaction in the presence of dTTP sets free long 5′ overhangs required for LIC. After annealing of the backbone plasmid to one constant as well as to one target-specific oligonucleotide, a 19 bp stretch remains single-stranded. Upon transformation into *E. coli*, a perfectly repaired, amplification-competent circular plasmid is obtained, containing the desired hybrid sgRNA sequence.

Apart from traditional restriction-ligation cloning, LIC provides an efficient assembly route for DNA cassettes bearing complementary single-stranded overhangs through making use of covalent ligation by bacterial ligases^14^,^15^,^16^. Long overhangs (≥10 nt) can be generated by T4 DNA polymerase, whose proof reading activity removes bases from dsDNA 3′ ends until reaching a sequence position where a specific dNTP supplied in the reaction can be incorporated by the enzyme’s polymerase activity. Based on LIC, we developed a cloning strategy that allows determining the spacer sequence of an sgRNA expression plasmid only by a single ssDNA oligonucleotide, thus enabling low-quantity chip-synthesized oligo pools to be used as input DNA without prior PCR amplification (Fig. 1). For that, we prepared a number of LIC-compatible plasmids, in which sgRNA expression is driven by a U6 promoter (Supplementary Fig. S1).

**Figure 2:**
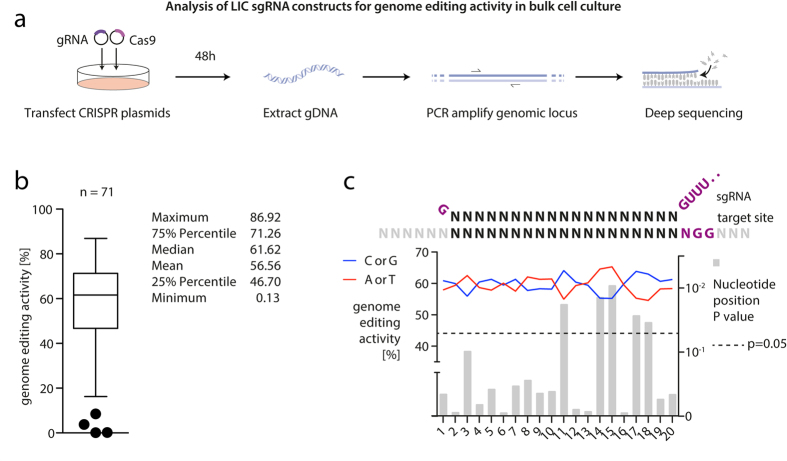
Analysis of LIC sgRNA constructs for genome editing activity. (**a**) sgRNA plasmids were generated by LIC and co-transfected with a Cas9 expression plasmid into HEK 293T cells. After 48 h cells were lysed, the targeted loci amplified by PCR, and the products analyzed using deep sequencing. (**b**) Genome editing activity of 71 sgRNAs is summarized in a box plot, whereas Tukey outliers are depicted as individual dots. (**c**) Shown is the mean genome editing activity of 67 CRISPR constructs (all CRIPSR, except the 4 outliers) calculated from the activity of all sgRNAs that have either a G/C or A/T at the indicated nucleotide position. The most 5′ base of the target site was determined as position 1, whereas the position 21 is the base N of the PAM (NGG). PAM is depicted in purple. Statistical analysis was carried out as an unpaired t-test, whereas the calculated p-values are depicted as bar charts. The dashed black line highlights the p-value threshold of 0.05.

For testing the method, we mixed 96 individual sgRNA oligonucleotides with a master mix containing LIC-ready pR-U6-gRNA backbone DNA as well as a PAGE-purified constant antisense oligonucleotide (Supplementary Table S1). Upon chemical transformation into *E. coli*, we observed 87.5% of resulting clones to be correct by Sanger sequencing. 71 of the assembled CRISPR constructs targeting human genes were tested for their genome editing activity by co-transfection with a Cas9 expression plasmid into HEK293T cells (Fig. 2a). Subsequent deep sequencing revealed a mean genome editing activity of 56.56% (Fig. 2b and Supplementary Table S2). Analyzing the genome editing activity of the CRISPR constructs as a function of their target site’s nucleotide composition, we found that the mean genome editing activity was higher in the group of CRISPR targets with an A or T at positions 14 or 15 and there was a preference for either C or G at positions 11, 17 and 18 (Fig. 2c and Supplementary Fig. S2). Considering these results, we *in silico* generated a library of CRISPR target sites covering the human protein coding genome based on the following rules: Only target sites in exons coding for the first half of an open reading frame were considered, while in case of multiple transcript variants being present, at least one site in every variant was targeted. Possible CRISPR target sites were identified by searching for the sequence 5′-N_21_-GG-3′ or 5′-CC-N_21_-3′ and only target sites harboring a GC-content of more than 35% in positions 2–20 were included. To avoid too many constraints for the sgRNA construct design, we focused on three of the five positional preferences (A/T at positions 14, 15 and C/G at position 18). Since previous studies had shown that mismatches in a so called PAM-proximal “seed” region of 6–13 bp are least tolerated by the CRISPR/Cas9 complex^3^,^6^, we favored target sites with unique seed regions within the human genome to avoid off target effects (approximately 90% of all library members fulfilled this criterion). One CRISPR target site for nearly every protein-coding gene fulfilling most of these criteria was chosen (for details see Supplementary Notes, Supplementary Fig. S3, S4 and Supplementary Table S3).

### Iterative synthesis of an arrayed sgRNA library

**Figure 3:**
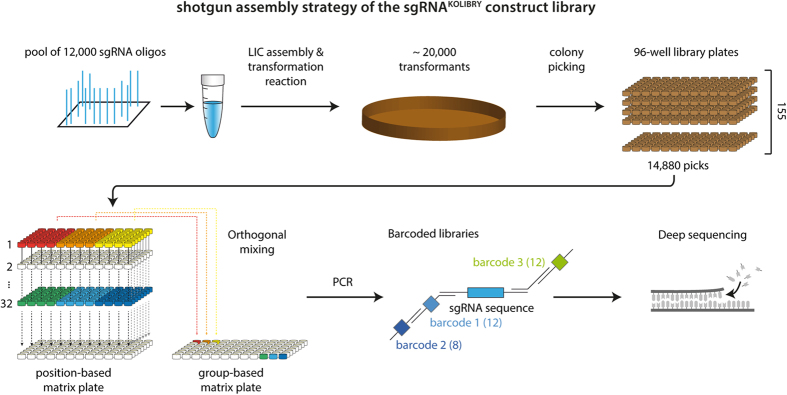
Shotgun sgRNA assembly strategy. Schematic diagram of the shotgun sgRNA assembly strategy using chip-synthesized oligo pools and of the orthogonal mixing strategy. The oligo pool was cloned by LIC and subsequently transformed into bacteria. Once colonies had grown on LB agar plates, clones were picked into single wells of 96-well plates (clone plates). Each set of 32 clone plates (each containing 32 × 96 clones) was mixed both position-wise and group-wise, wherein a group corresponds to the equally colored part of an individual clone plate. Two resulting matrix plates per set are used as templates for a dual-PCR. The first PCR amplifies the sgRNA sequence and adds one of 12 possible barcodes specific for the individual matrix plates (6 for the position-based matrix plates and 6 for the group-based matrix plates = barcode 1). The second PCR amplifies the first PCR product, introduces 96 barcode combinations (8 × 12 = barcodes 2 and 3) that are specific for every position on the 96-well matrix plates, as well as Illumina linker sequences. Finally, the PCRs are subjected to deep sequencing.

Using chip-based synthesis, we obtained an oligonucleotide pool (pool A) with 19,956 71-mer sgRNA coding ssDNA sequences. To construct the arrayed sgRNA library we decided to use a vector backbone that allows lentiviral packaging and that also contains a CMV-driven GFP gene for transfection or transduction control (pL-U6-gRNA, Supplementary Fig. S1). Following the LIC assembly process and transformation, we expanded 29,568 individual colonies in 308 96-well plates (Fig. 3). To allocate the position of an individual sgRNA construct in our arrayed CRISPR library without sequencing all clones individually, we created an orthogonal pooling and deconvolution scheme comparable to previously described schemes^17^,^18^, for which the 308 96-well plates were separated into 10 individual sets of 32 plates each. Within each set, we pooled 32 clones in a position-based as well as in a group-based matrix. For example, all 32 clones in position A1 of one plate set were pooled into position-based matrix A1, and in addition 32 clones from plate 1 (A1 − H4) were pooled into group-based matrix I. This approach led to 96 position-based pools (A1 − H12) and to 96 group-based pools (I − XCVI) for the first plate set. By a two-step PCR protocol we attached Illumina-compatible linker sequences as well as three barcodes to identify all 32 members of each pool by deep sequencing (Fig. 3). The design of the barcoding scheme allowed differentiation of up to 1,152 individual pools, translating to 18,432 individual clones being possibly analyzed in a single deep sequencing run.

From the first sequencing round, 6,514 unique, error-free library members could be unambiguously allocated to a defined position on the clone plates. Individually barcoded deep sequencing confirmed all position calls from the orthogonal mixing to be correct (Supplementary Fig. S5a). 54.2% of the clones contained a correct sgRNA insert, whereas mutations were due to the oligo chip synthesis process and not the LIC assembly technology. However, we observed a non-random overrepresentation of sequence duplicates on clone plates picked from a single agar plate (Supplementary Fig. S5a), likely due to clonal amplification of bacteria before agar plate streaking. Clonal duplicates inevitably result in a reduced coverage of the picked library and also hamper sequence calling by orthogonal mixing (Supplementary Notes). To this end, for subsequent sgRNA assemblies, we spared the usual 1 h incubation step of the bacteria prior to plating to eliminate the problem of clonal duplicates (Supplementary Fig. S5b). Consequently, the coverage distribution of individual sequences matched the expected random distribution of a non-redundant library (Supplementary Fig. S5c).

**Figure 4:**
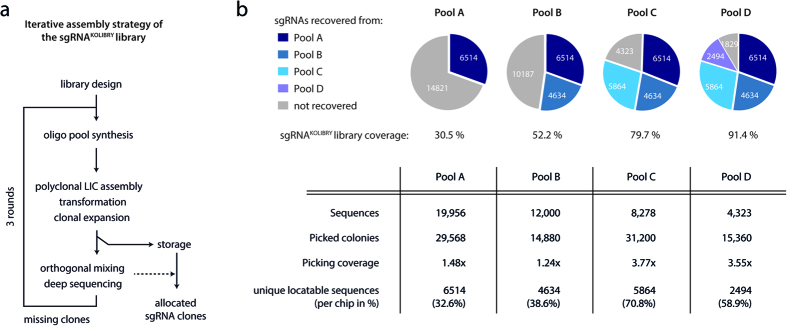
Iterative assembly of the sgRNA^KOLIBRY^ library. (**a**) Iterative assembly strategy to generate large sgRNA libraries. (**b**) Pie charts and table summarizing the results and performance of the iterative assembly rounds of the sgRNA^KOLIBRY^ library.

Based on this improved protocol, we went on to complete the library with three subsequent rounds of synthesis, clone picking, and sequencing as described above (pool B, C and D, Fig. 4). Pool B contained 12,000 sgRNA library constructs and with a picking coverage of 1.24 fold (14,880 colonies) we identified 4,635 additional library constructs. From pool C, comprising of 8,278 sgRNA library members, we picked 31,200 colonies (3.77 fold picking coverage) to allocate an additional set of 5,864 correct library sgRNAs. Pool D contained the remaining 4,323 sgRNAs and we picked 15,360 colonies (3.55 picking-fold) from this pool to successfully allocate 2,494 additional clones. Altogether, we obtained an overall library coverage of 91.4% (Supplementary Table S4).

### Validation of library performance

**Figure 5:**
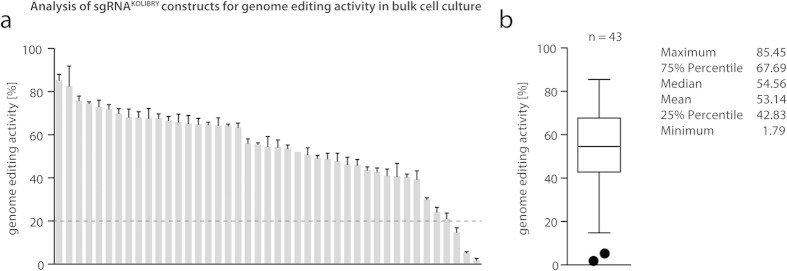
Analysis of sgRNA^KOLIBRY^ library constructs for genome editing activity. Shown are the genome editing activities of 43 picked sgRNA^KOLIBRY^ library constructs. The constructs were transfected together with a Cas9 expression plasmid into HEK 293T cells and genome editing activity was then determined by deep sequencing. (**a**) The activity of each sgRNA construct is depicted as mean values + SEM from three independent experiments. The dashed line indicates a threshold of 20% genome editing activity. (**b**) The box plot summarizes the genome editing activity of 43 sgRNAs, whereas Tukey outliers are depicted as individual dots.

To validate the genome editing performance of the constructed library we picked 43 sgRNA constructs targeting 43 genes associated with cytosolic nucleic acid sensing pathways (Supplementary Table S5). These sgRNA constructs were co-transfected with a Cas9 expression plasmid into HEK293T cells. Two days later genomic DNA was extracted to perform locus-specific deep sequencing. Analyzing these data revealed a mean genome editing activity of 53.14%, with 93% of all sgRNAs (40 of 43) having an activity higher than 20% (Fig. 5). Of note, the genome editing activity of this set of sgRNA constructs was not significantly higher when compared to the previous panel of 71 randomly designed sgRNAs side by side (data not shown). However, a formal quantitative comparison cannot be performed given the fact that different backbones were used for these studies (5.5 kb pR-U6-gRNA vs. 7.3 kb pL-U6-gRNA).

**Figure 6:**
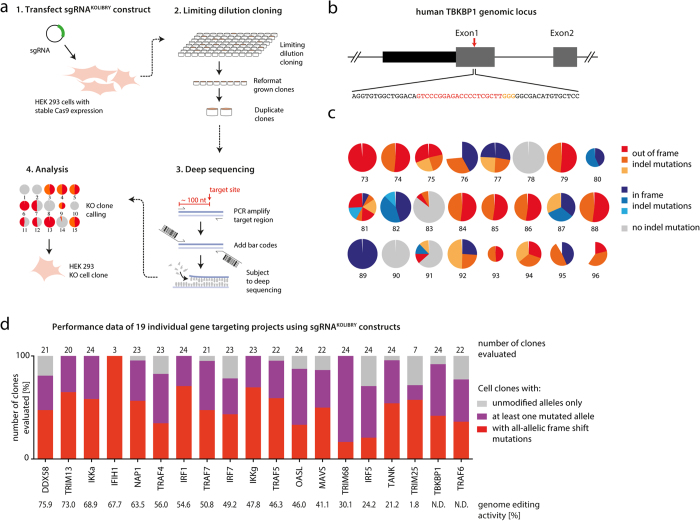
Generation of KO cell lines utilizing the sgRNA^KOLIBRY^ library. (**a**) Schematic diagram of knockout cell line generation. (**b**) Schematic view of the genetic locus of the human TBKBP1 gene. The red arrow indicates the location of the target site of the sgRNA. The red sequence highlights the target site whereby the orange sequence indicates the PAM. (**c**) OutKnocker was used to analyze deep sequencing data obtained from gene targeting experiments. Each pie chart represents the deep sequencing data from an individual cell clone. Each pie piece sums up all reads that have the same indel mutation, with its size representing the proportion of total reads analyzed. Grey pie charts show reads without indel mutations, blue colors indicate indel mutations that are in frame and red colors indicate indel mutation reads that are out of frame, whereas the different shades of blue and red represent individual indel mutations in one pie chart. Depicted is an exemplary output for the generation of TBKBP1 knockout HEK cells. (**d**) Shown is a histogram summarizing the results of 19 individual gene targeting projects. Grey bars show the number of clones that carry only unmodified alleles, while purple bars indicate clones with at least one mutated allele and red bars clones with all-allelic frame shift mutations.

Finally, we went on to verify that the library is suitable for the generation of knockout cell lines. To do so, we selected 22 sgRNA constructs and transfected them into HEK293T cells stably expressing Cas9 (Fig. 6a). Two days following sgRNA transfection, cells were re-plated under limiting dilution conditions. After two weeks 24 single cell clones per target gene were harvested and expanded for subsequent genotype analysis. Using a workflow that allows the multiplexed analysis of several cell clones from different gene targeting experiments in parallel^19^, the clones were analyzed for indel events at the genomic locus of interest. One exemplary result is depicted for the target gene TBKBP1 (Fig. 6b). In total, sequencing data of 19 individual genes targeted provided enough read numbers for subsequent analysis. All of these yielded at least three cell clones harboring all-allelic out-of-frame indel mutations in the target gene, whereby knockout efficiencies of up to 100% were achieved (Fig. 6c and Supplementary Fig. S6). We additionally validated the protein expression of six different target genes for which three individual knockout cell clones were available. These experiments revealed that all knockout clones lacked the expression of the respective target gene, as observed by immunoblotting (Supplementary Fig. S7). Similar results were obtained when employing constructs of this library to generate genetic knockouts in other cell lines and when using different means of transgene delivery, e.g. by electroporation of THP1 cells^16^,^20^,^21^,^22^. We furthermore tested the genome editing activity of two sgRNA constructs that were delivered by lentiviral transduction. Doing so, we observed that virally transduced sgRNAs induced indel mutations at the desired locus, whereas the overall genome editing activity was tightly correlated with the amount of cells being transduced (Supplementary Fig. S8). All in all these data confirmed that constructs from our sgRNA library are well suited to generate knockout cell lines.

## Discussion

We here present a LIC-based assembly method and an orthogonal sequence validation method for CRISPR sgRNA constructs that facilitate rapid iterative assembly and validation of large sgRNA libraries. Using this approach, we were able to recover 19,506 unique sequence-validated sgRNAs (91.4% library coverage) in four iterative clone picking rounds. To fulfill this task, we made use of the LIC assembly technology that only relies on a single fluid mixing step and no enzymatic amplification, yet stands out with high specificity and efficiency. In the absence of amplification steps, we obtained an evenly distributed polyclonal sgRNA pool, which could subsequently be used to generate an arrayed, monoclonal library with reasonable effort. Of note, an even distribution of library sequences is not only required for an optimal coverage, but also constitutes an important requirement to apply orthogonal mixing and deconvolution, since redundancy dramatically impacts on reliable allocation of sequences (Supplementary Notes).

Testing a randomly selected set of 71 sgRNA constructs for genome editing activity in human cells, we in general observed high genome editing activities. The mean activity level was ranging around 55%, with a low interquartile range of about 23%. Of note, in this panel only five sgRNAs displayed genome editing activities below 20% and two of these constructs encoded for four consecutive uridines, which is known to terminate RNA polymerase III driven expression^23^. To reveal possible targeting rules, we analyzed the genome editing activity of sgRNA sequences as a function of the position-wise C/G content of the sgRNA molecule rather than taking all individual base identities into account. We chose this reduced classification given the limited number of data points available, but also given the fact that this categorization represents a well-proven, simple measure of the local stability of a double stranded nucleic acid molecule. In this regard it is interesting to note that the recently published structures of RNA:DNA hybrids bound to Cas9 imply that the Cas9 protein binds to the sgRNA and the protospacer region in a largely sequence independent manner^24^,^25^,^26^. This suggests that the impact of the sgRNA sequence on Cas9 activity is rather due to thermodynamic properties of the respective sgRNA molecule or the sgRNA:protospacer complex than on Cas9 binding. Analyzing our data, we observed a trend towards C/G rich regions being favored at positions 17 and 18 of the protospacer region, whereas higher genome editing activity was observed for protospacer regions harboring A/T at positions 14 and 15. In line with our results, another study, in which data from pooled sgRNA screens were analyzed to predict possible targeting rules, suggested that C/G at positions 17 and 18 of the sgRNA molecule were associated with higher genome editing activity, whereas being less favorable at positions 14 and 15 of the sgRNA molecule^11^. Moreover, another group reported similar results for positions 14 and 15 of the sgRNA, while obtaining differing results for other positions^27^. All in all, we speculate that these results reflect the existence of a favorable thermodynamic profile of the protospacer region, the sgRNA:protospacer complex or the sgRNA molecule itself to facilitate functional enzyme activity, yet additional experiments are required to substantiate this hypothesis. Indeed, studying the genome editing activity of sgRNA constructs that were designed according to these rules did not display higher activities. However, in this regard it has to be noted that we switched to a considerably larger plasmid backbone to generate our genome-wide sgRNA library.

Designing our genome-wide sgRNA library, we favored sgRNA constructs abiding to the aforementioned position-based rules and we additionally premised an overall GC content of >35%, a constraint which has been experimentally validated by other groups^11^,^27^,^28^. Due to the fact that almost all sgRNAs show genome editing activity we decided to generate one sgRNA per gene, which we consider sufficient for the application of this library to generate knockout cells. However for arrayed screening purposes a higher coverage would be advantageous.

To render a target gene dysfunctional at the level of protein expression, we chose to place sgRNAs within the earliest possible coding exon, but at least within the first half of the coding region of a gene. An indel mutation in an early coding exon can shift the reading frame of the downstream coding region. This can result in premature translation-termination codon (PTC) and as such lead to a C-terminally truncated protein product. At the same time, the presence of a PTC can provoke the degradation of the mRNA due to nonsense-mediate mRNA decay, a mechanism that is most effective if the PTC is located in the first half of the open reading frame^29^. In line with these considerations, recent studies reported increased functional knock out rates for sgRNAs targeting early coding exons^11^,^27^.

At the time of designing our library, we opted for a stringent measure to avoid sgRNAs with putative off-target activities by assessing the PAM-proximal 13-mer sgRNA seed sequence to be unique in the genome^5^. Since then, several groups have reported on significant off-target mutagenesis by the CRISPR/Cas9 system^30^,^31^,^32^,^33^,^34^,^35^,^36^,^37^,^38^, suggesting that our specificity measures were probably not sufficient to eliminate all possible off-target effects of our sgRNAs constructs. Of note, while we have not experimentally assessed the frequency of putative off-target effects of our sgRNA construct design, we speculate that a large sample size would be required to obtain informative results that would allow reliable predictions for the entire collection. Nonetheless, recent studies analyzing single cell clones for CRISPR/Cas9 mediated off-target effects at a genome-wide level revealed a low incidence of such events. This suggests that off-target indels might not be a major concern when generating clonal knockout cell lines^39^,^40^. Indeed, it appears that when generating knockout cell lines by single cell cloning, clonal heterogeneity obtained irrespective of exogenous manipulations may indeed introduce a bigger variability than putative off target effects themselves^39^. Given the fact that our library was primarily designed for the generation of knockout cell lines, we therefore assume that off-target effects should not be of primary concern when applying the here-described sgRNA constructs for this purpose.

When employing constructs of our sgRNA library to generate knockout cells, we observed very high on-target activity, thus confirming the usefulness of our collection. Interestingly, we did not observe that knockout efficiencies correlated with the measured polyclonal activities of sgRNAs. In contrast, even sgRNA constructs with low transient genome editing activities yielded high amounts of knockout cell clones. The high knockout frequencies might be due to stable Cas9 expression in the cell line used and at the same time it is possible that the prolonged sgRNA and Cas9 expression during the cloning procedure leads to a high, cumulative genome editing frequency.

Taken together, we present an arrayed genome-wide sgRNA library designed to target human protein coding genes for loss of protein expression. Constructs of this library display high genome editing activity in polyclonal cell culture and are highly functional in generating knockout cell clones. This library will be a useful tool for dedicated, large-scale knock out cell generation projects or even the conduction of pathway- or genome-wide genetic screens. The sgRNA **K**nock**O**ut **LIBR**ar**Y** (sgRNA^KOLIBRY^) will be freely available as a resource to the research community.

## Methods

### CRISPR sgRNA plasmid assembly

10 μl of the sgRNA entry vector (200 ng/μL in H_2_O) were digested with ApaI, SpeI, and EcoRV (FastDigest, Fermentas) at 37 °C for 2 h. The digestion mix was separated on an agarose gel (1%), the cleaved product was purified using an innuPrep gel extraction kit (Analytik Jena) and eluted in 15 μl H_2_O (final concentration: 70 ng/μl). The linearized vector was chewed using T4 DNA polymerase (Enzymatics) for 5 min at 27 °C in the presence of dTTP as a stopping nucleotide. After heat-inactivation, the reaction was diluted 10-fold in 2× NEB2 buffer in the presence of 0.5 μM short universal reverse strand oligo (PAGE purified, 100 μM, IDT). Gene-specific sgRNA oligos (desalted, 100 μM, IDT) were diluted 1:400 in H_2_O and then mixed at equal volume with the vector pre-dilution and annealed in a PCR cycler (C100, Biorad). For a detailed protocol please refer to the Supplementary Methods section.

### Arrayed transformation and bacterial culture

5 μl of the assembly mix were transformed using 50 μL of chemo competent DH10b *E. coli* at 42 °C for 45 sec. Next, the bacteria where incubated with 450 μL LB medium at 37 °C, 900 rpm for 1 h. The bacteria were streaked out on LB-Agar plates supplemented with 100 μg/ml Ampicillin and incubated at 37 °C over night. On the next day LB medium containing 100 μg/ml Ampicillin was inoculated with a single colony and incubated at 37 °C, 900 rpm over night. The following day DNA was purified using standard silica spin column kits according to the manufacturer’s protocol.

### CRISPR/Cas9 transfection

HEK 293T or HEK 293-CAS9 cells were plated at a density of 2 × 10^4^ cells per 96-well. On the next day, CRISPR plasmids were transfected using GeneJuice transfection reagent (Merk Millipore) according to the manufacturer’s protocol. pRZ-mCherry-Cas9 and sgRNA constructs were transfected at a ratio of 150 ng to 50 ng per well. All vector sequences can be found in Supplementary Table S7.

### Cell lysis

The medium was removed and the cells were lysed in 30 μl of the following lysis buffer: 0.2 mg/ml proteinase K, 1 mM CaCl_2_, 3 mM MgCl_2_, 1 mM EDTA, 1% Triton X 100, 10 mM Tris pH 7.5. The reactions were incubated at 65 °C for 10 min and at 95 °C for 15 min.

### Dual PCR barcoding

First-level PCR reactions are performed using 2 μl PCR-compatible lysate as a template and a locus specific primer for a 12.5 μl Phusion PCR reaction (Thermo Scientific) according to the manufacturer’s protocol (annealing temperature: 60 °C, elongation time: 15 sec, 18 cycles). Of this reaction, 1 μl is transferred to a second-level PCR using the same cycling conditions and a second set of primers that amplifies the first PCR, while adding 96 individual barcode combinations and an Illumina specific sequencing linker. For all primer sequences see Supplementary Table S6.

### Deep sequencing

Crude PCR products were pooled and size-separated using a 1% agarose gel run at 100 V. After visualization with ethidium bromide under UV light, DNA bands from 350 bp to 500 bp were cut out and purified using Jena Analytik innuPrep gel extraction kit according to the manufacturer’s protocol. Eluted DNA was precipitated by adding 0.1 volumes of 3 M NaOAc (pH 5.2) and 1.1 volumes of isopropanol. After centrifugation for 15 minutes at 4 °C, the resulting pellets were washed once in 70% EtOH and air-dried. 35 μl water was added, non-soluble fractions were spun down and removed, and the DNA concentration was quantified using a NanoDrop spectrophotometer (Thermo scientific). Deep sequencing was performed according to the manufacturer’s protocol using the MiSeq (Illumina) bench top sequencing system and a 300-cycle-V2 cassette. Data were obtained in FASTQ format.

### Library assembly

Chip-synthesized oligo pools were obtained from CustomArray. The oligo pools were diluted to a final concentration of 0.04 ng/μl and incubated with the LIC-ready vector pre dilution as described for arrayed assemblies.

### Deep sequencing evaluation of polyclonal genome editing activity

Using a modified version of the OutKnocker software^19^, sequencing reads were compared to a given reference sequence. Reads with indel mutations spanning or touching CRISPR target sites were counted as mutated reads. To calculate the mutation frequency, the number of mutated reads was divided by the total number of reads.

### Orthogonal mixing and deconvolution

Bacterial liquid cultures were mixed using a Biomek FXp liquid handling station (Beckman Coulter) into group-based matrix plates and position-based matrix plates as indicated in Fig. 3. The first PCR amplifies the sgRNA sequence and adds one of 12 possible barcodes specific for the individual pool plates using the primers gRNA_fwd_1-gRNA_fwd_12 and gRNA_rev (barcode 1 in Fig. 3). The second PCR amplifies the first PCR, while adding 96 individual barcode combinations and an Illumina specific sequencing linker (barcode 2 and 3 in Fig. 3). For all primer sequences see Supplementary Table S6. Deconvolution was performed using a custom-written javascript program.

### Generation of knock out cell lines

HEK 293 cells stably expressing mCherry-CAS9 (HEK 293-CAS9) are HEK-Blue™ IFN-α/β Cells (purchased from Invivogen) that were stably transduced with a mCherry-CAS9 and cloned by limiting dilution cloning. HEK 293-CAS9 cells were plated at a density of 2 × 10^4^ cells per 96-well. Cells were transfected with 200 ng of sgRNA expression plasmid per well using GeneJuice (Merck Millipore). Two days after transfection, cells were trypsinized, counted, and monoclones were seeded at a density of 0.8 cells per 96-well in three 96-well plates. After two weeks, 24 monoclones per targeted gene were picked, expanded, and genotyped as described^19^. Only monoclones with at least 50 alignable reads were evaluated. No mycoplasma contamination was detected in regular screenings of our cell lines.

### Viral transduction

HEK293T cells were seeded at a density of 3 × 10^5^ cells/ml in a six well dish the day before transfection. A six well was transfected with the library plasmid containing the sgRNA, a viral packaging plasmid expressing pCMVΔ8.91 and a pseudotyping plasmid expressing VSV-G in a ratio of 10:10:1 with Lipofectamine 2000 according to the manufacturer’s instruction. One day after transfection the medium was changed to a standard medium containing 30% FCS. 48 h later the virus was harvested and filtered. HEK 293-CAS9 were seeded at a density of 3 × 10^5^ cells/ml the day before transduction in a 96 well plate. On the next day cells were transduced with virus. A titrating from 100 μl to 50 μl, 25 μl, 12.5 μl and finally 0 μl per well was performed. The wells were filled up to 100 μl with standard medium. Two days after transduction the cells were fixed with paraformaldehyde and analyzed by FACS. Lysis and analysis by deep sequencing was done as described above.

### Western blotting

Laemmli lysates of knockout cell clones were subjected to SDS-PAGE and subsequent immunoblotting. Membranes were probed for β-actin, IKKα, IRF1, MAVS, Sintbad, TANK and TRAF6. Antibodies were obtained from Cell Signaling (anti-IKKα #2682, anti-IRF1 #8478, anti-Sintbad #8605, anti-TANK #2141 and anti-TRAF6 #8028) and Santa Cruz Biotechnology (anti-MAVS sc-166583, anti- β-actin-HRP sc-47778 and secondary antibody anti-rabbit-HRP sc-2004).

### Statistical analysis

Outliers (Fig. 2b or Fig. 5b) were identified using the Tukey’s outlier filter. A two-tailed, unpaired t-test with or without Welch’s correction (an F test was performed to identify unequal variances) was used to compare genome editing activities of the pool of sgRNAs with either A/T or C/G at every position of the target region (Fig. 2c and Supplementary Fig. S2).

### Availability

All functionally validated constructs are available upon request.

## Additional Information

**How to cite this article**: Schmidt, T. *et al.* Synthesis of an arrayed sgRNA library targeting the human genome. *Sci. Rep.* **5**, 14987; doi: 10.1038/srep14987 (2015).

## Supplementary Material

Supplementary Material

Supplementary Table S3

Supplementary Table S4
