# Supplementary material for: Synthesis of an arrayed sgRNA library targeting the human genome
Source: Sci Rep. 2015 Oct 8;5:14987. doi: 10.1038/srep14987 (PMC4597219; doi:10.1038/srep14987)
Supplement: Supplementary Material [file srep14987-s1.pdf]

Synthesis of an arrayed sgRNA library targeting the human genome

Tobias Schmidt[1,+], Jonathan L. Schmid-Burgk[1,+], and Veit Hornung[1,*]

[1] Institute of Molecular Medicine, University Hospital, University of Bonn, Sigmund-Freud-Str. 25 , 53127 Bonn, Germany.

[+] T.S. and J.L. S.-B. contributed equally to this work

[*] Correspondence should be addressed to V.H. (veit.hornung@uni-bonn.de)

Supplementary Material

Supplementary Figures 1-8

Supplementary Notes

Supplementary Methods

Supplementary Tables 1, 2, 5, 6 and 7

Supplementary Tables 3 and 4 (see additional XLS spreadsheets)

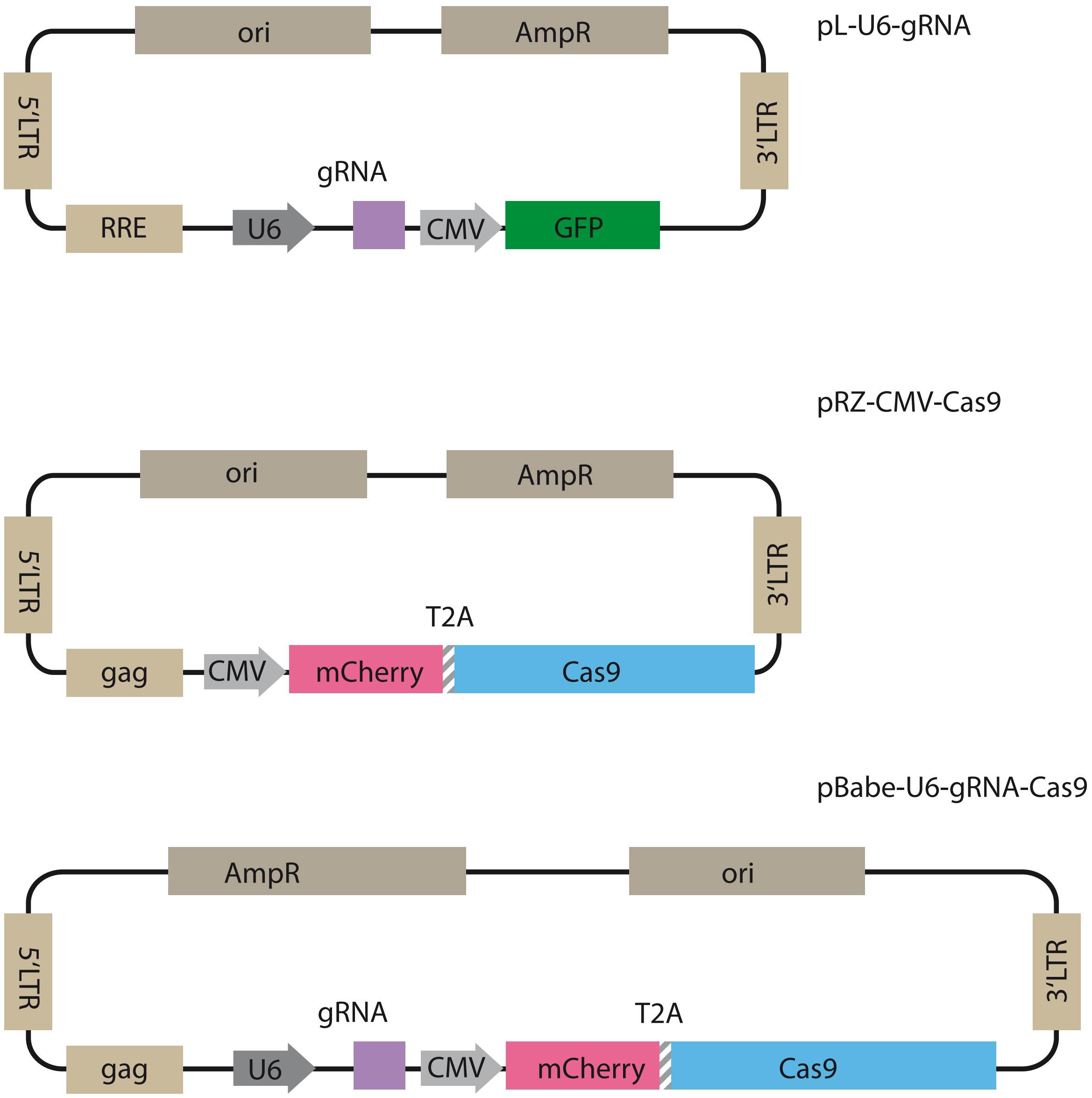

Supplementary Figure S1 | CRISPR/Cas9 Vectors. Depicted are three different vector: pL-U6-gRNA contains a LIC ready sgRNA cassette driven by a U6 promoter, followed by a CMV promoter-driven GFP cassette. pRZ-CMV-Cas9 contains an mCHerry-Cas9 cassette that is seperate by a T2A peptide and driben by a CMV promoter. pBABE-U6-gRNA-Cas9 contains both a LIC ready sgRNA cassette driven by a u6 promoter and a mCherry-Cas9 cassette that is seperated by a T2A peptide and is under control of a CMV promoter. pL-U6-gRNA additionally contains lentiviral packaging elements while pRZ-CMV-Cas9 and pBabe-U6-gRNA-Cas9 contain retroviral packaging elements.

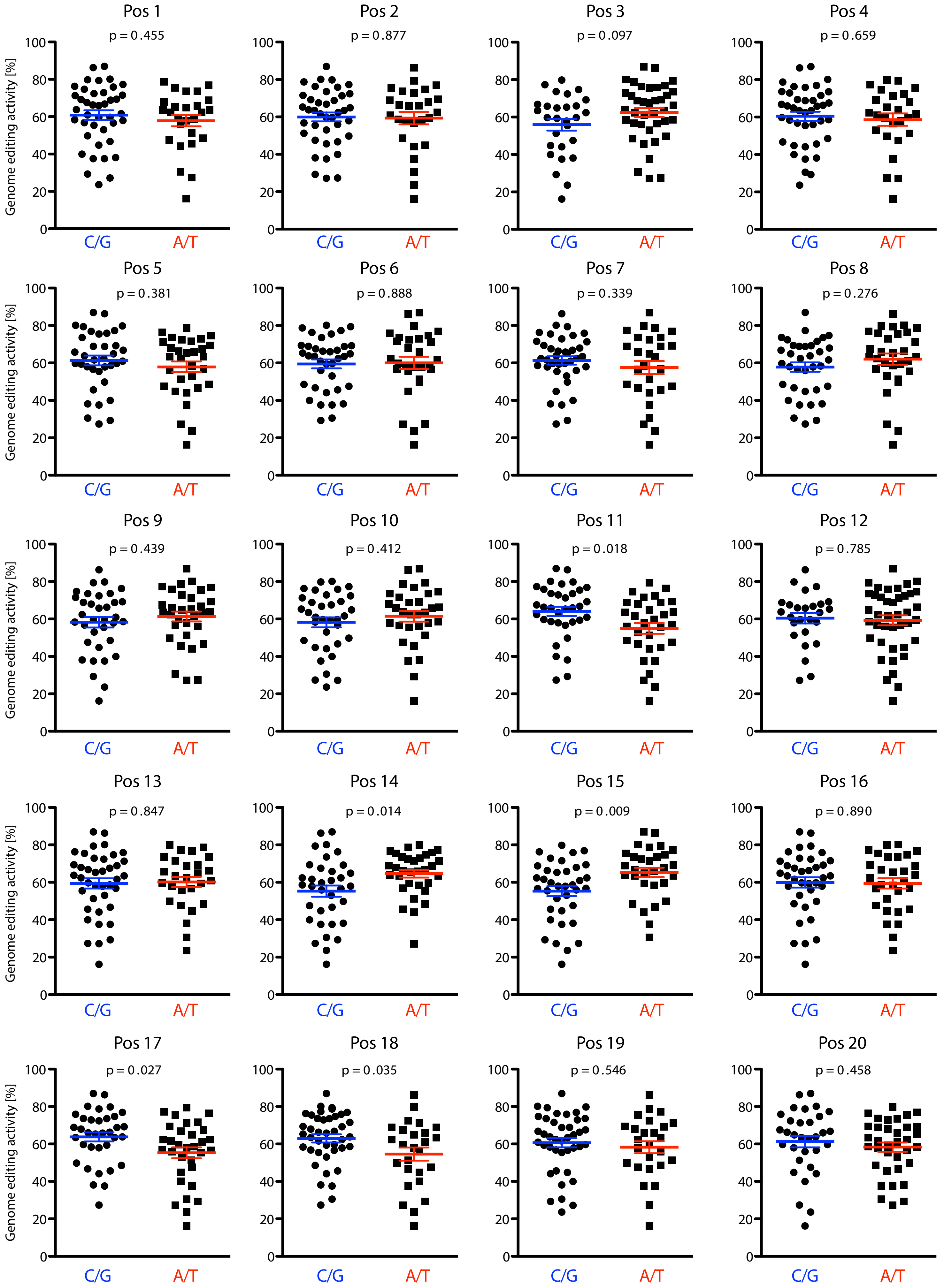

Supplementary Figure S2 | Base pair composition of the target site. Shown is the genome editing activity of the 67 sgRNA constructs depicted in Figure 2 as a function of their C/G or A/T content at position 1 to 20 (N of NGG in the PAM is position 21). Mean ± SEM is highlighted in colors (G/C blue, A/T red). Statistical analysis was performed using a two-sided, unpaired t-test, whereas nominal p values are depicted.

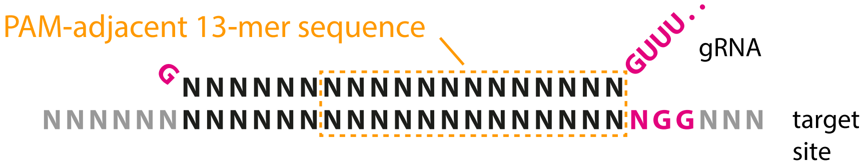

1.) Fetch all annotated mRNAs (NCBI RefSeq).

↓

2.) Identify longest open reading frame (ORF)

- If an in-frame *ATG* is present within the first 80 bases of an ORF: shift ORF boundary to that *ATG*.
- Select the first half of the ORF for target site identification.

↓

3.) Generate list of suitable spacer/protospacer regions (5'-N_{21}GG-3' or 5'-CCN_{21}-3')

- must map to genome (UCSC hg19 assembly).

↓

4.) Calculate activity score based on targeting algorithm:

- A or T at position 14 = score +1
- A ot T at position 15 = score +1
- G or C at position 18 = score +1
- if GC content in region 2 ... 20 < 35%: set score = 0

↓

5.) Determine number of target sites of the PAM-adjacent 13-mer sequence within genome.

↓

6.) Pick spacer/protospacer region according to the following priorities (list 1 - 4):

activity score = 3 or 2		activity score = 1 or 0	
target sites = 1	target sites = 2	target sites = 1	target sites = 2

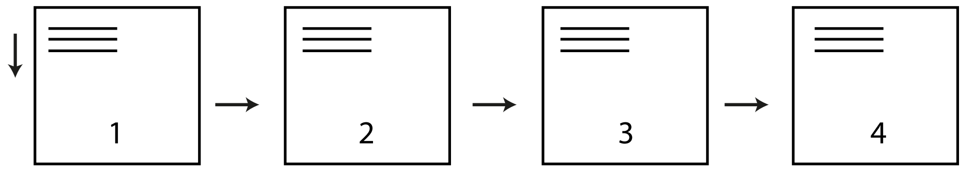

↓

7.) Eliminate duplicate spacer/protospacer regions from total pool.

Supplementary Figure S3 | Description of the sgRNA^{KOLIBRY} library design. A detailed workflow of the algorithm for the design of a genome-wide human CRISPR library sgRNA^{KOLIBRY} is depicted (please see Supplementary Note 2 for details).

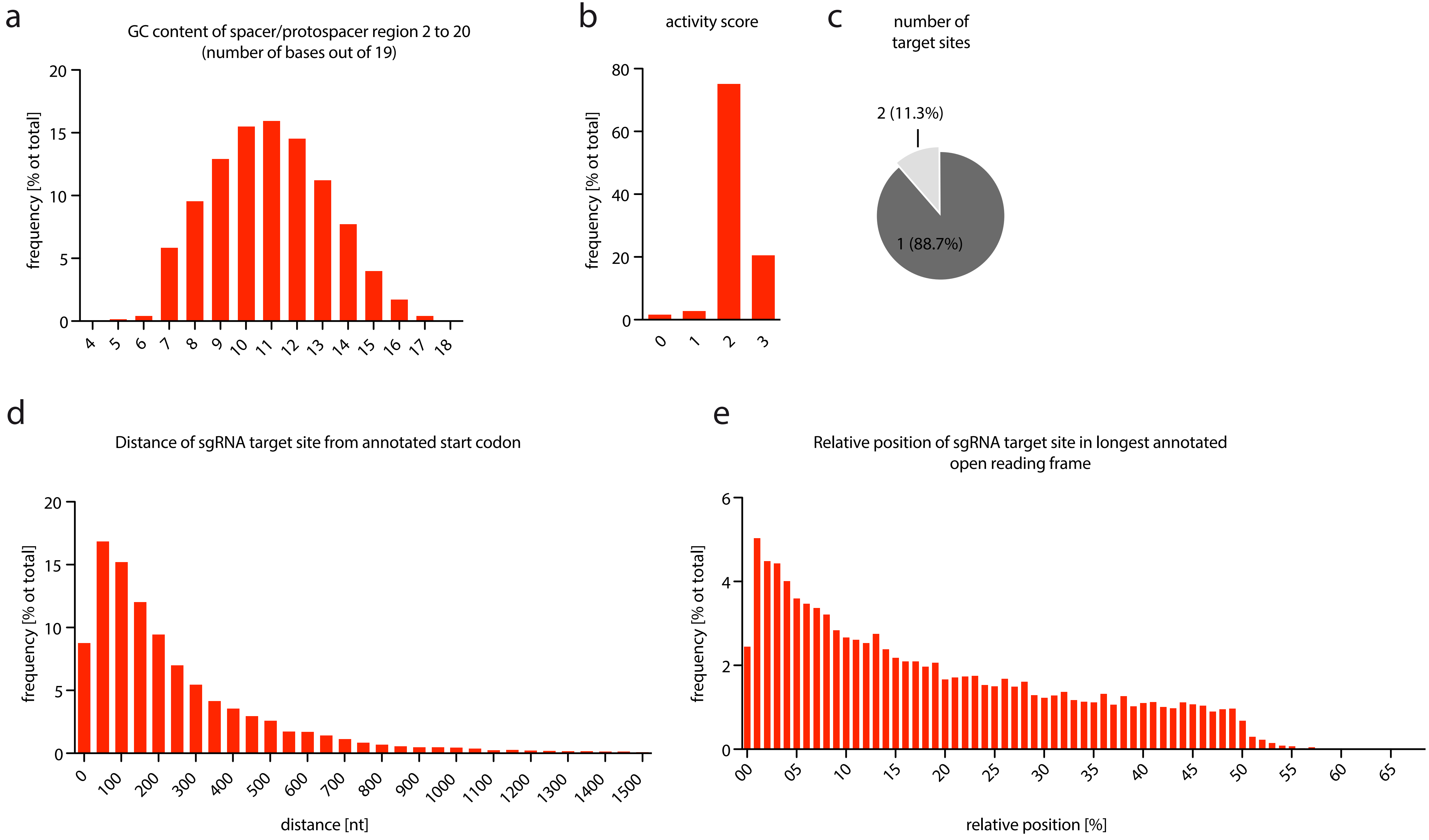

Supplementary Figure S4 | Design characterisitics of the sgRNA^{KOLIBRY} library. Depicted are histograms that show (**a**) the GC distribution, (**b**) the activity score according to the library design algorithm and (**c**) the pie chart shows the proportion of sgRNAs with one or two target sites of the PAM-adjacent 13-mer region. (**d** and **e**) Histograms depict the absolute (bin width 50 nt) and relative distance (bin width 1%) of the sgRNA target site from the start codon. Note that target sites beyond 50% of the ORF are obtained due to the fact that an offset of 80 nt from the first start codon is considered for some targets.

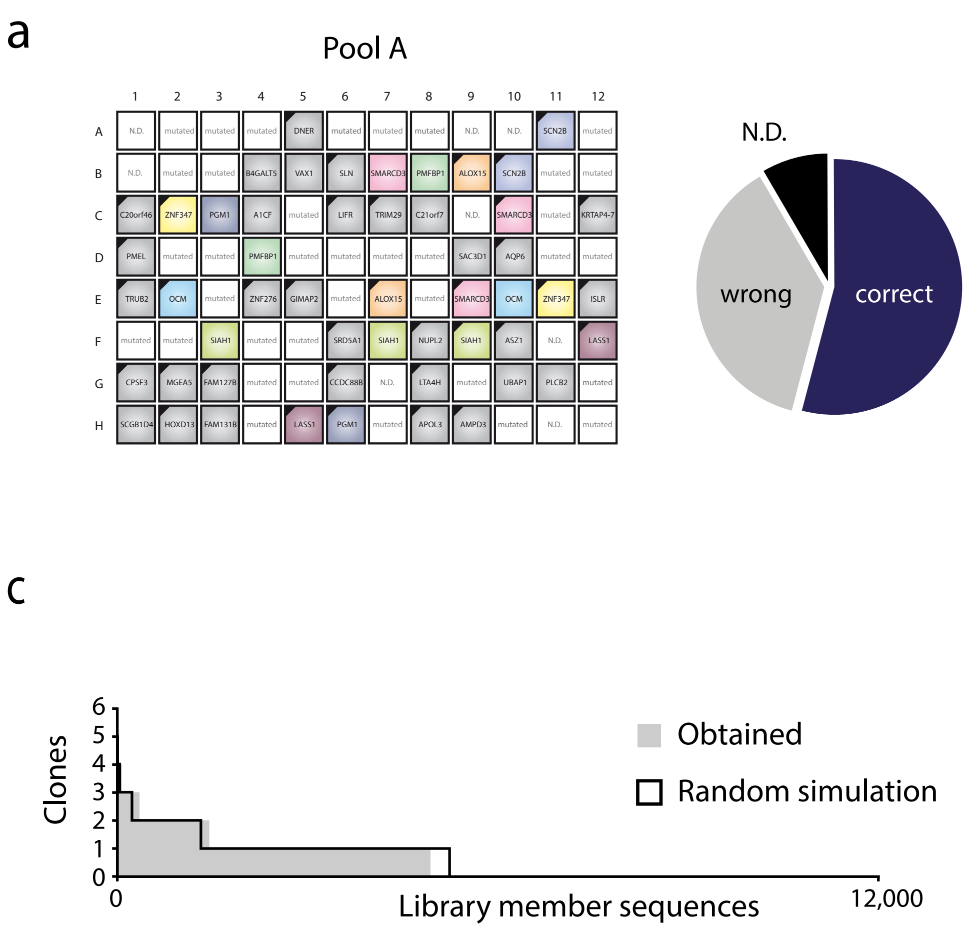

Supplementary Figure S5 | Elimination of clone duplicates validated by individual sequencing of sgRNAs from two individual pools. Sequencing results from 96 representative sgRNA library clones picked from pool A (**a**) or pool B (**b**). Indicated are the identifiers of the genes targeted by the retrieved correct sgRNA sequences, mutated sgRNA sequences (mutated), and plasmids that could not be sequenced (N.D.). Colors highlight sgRNA sequences that were observed more than once on the same plate. Black triangles in a well indicate that the respective clone was also identified by orthogonal mixing. Pie chart summarizing the identified sgRNA oligos that are depicted in (**c**). The coverage of individual library members picked from pool B is depicted as compared to a simulation assuming a complete random distribution of library members with no sequence or founder bias.

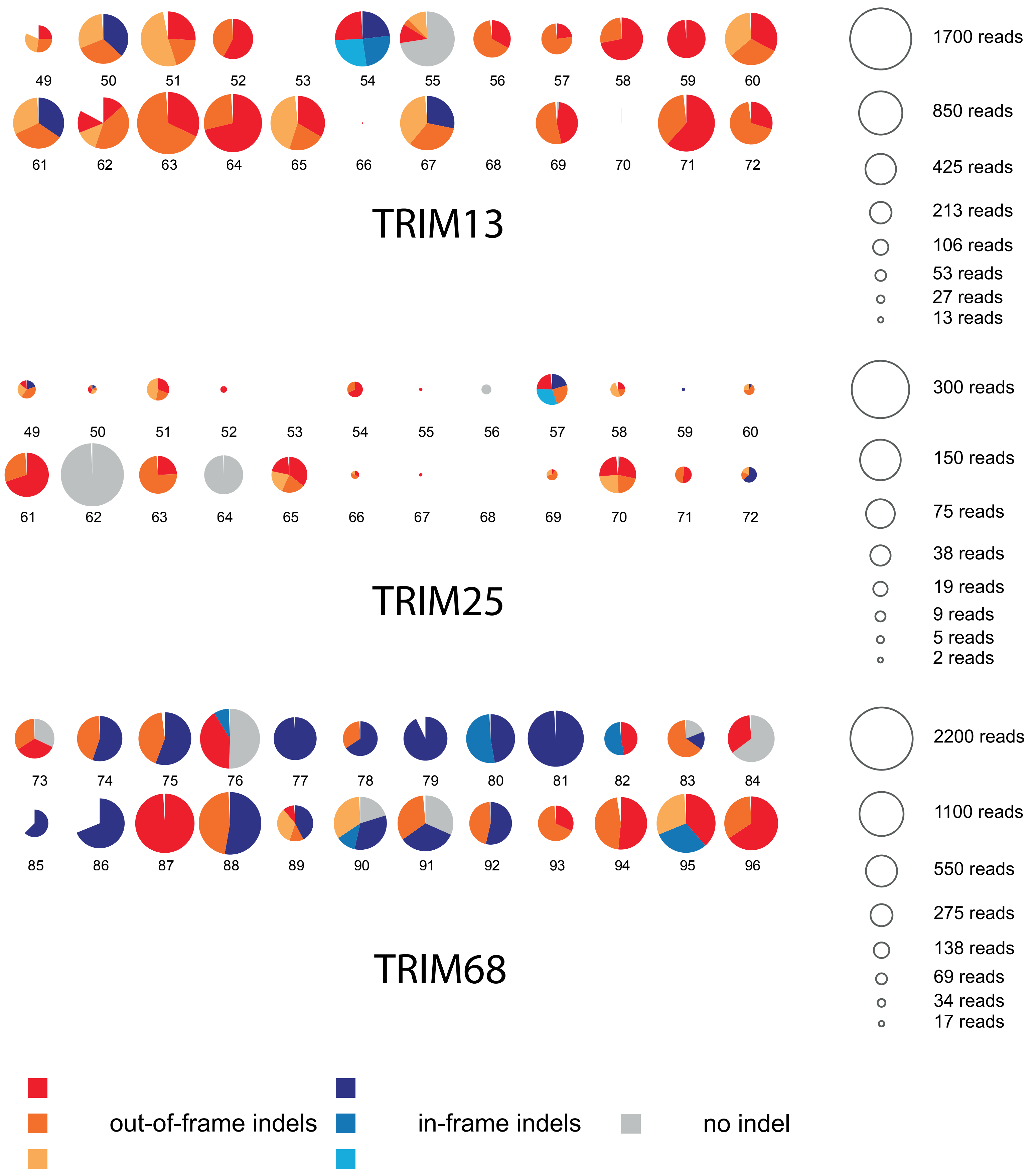

Supplementary Figure S6 | OutKnocker analysis of 19 individual gene targeting projects. Depicted is the deep sequencing result of 19 gene targeting projects analyzed by the web tool OutKnocker. For every gene targeting project 24 clones were picked and sequenced. Each pie pieces sums up all reads that have the same mutation and represents the proportion of total reads analyzed. Grey pie charts show reads without indel mutations, blue colors indel mutations that are in frame, whereas red colors indicate indel mutation reads that are out of frame.

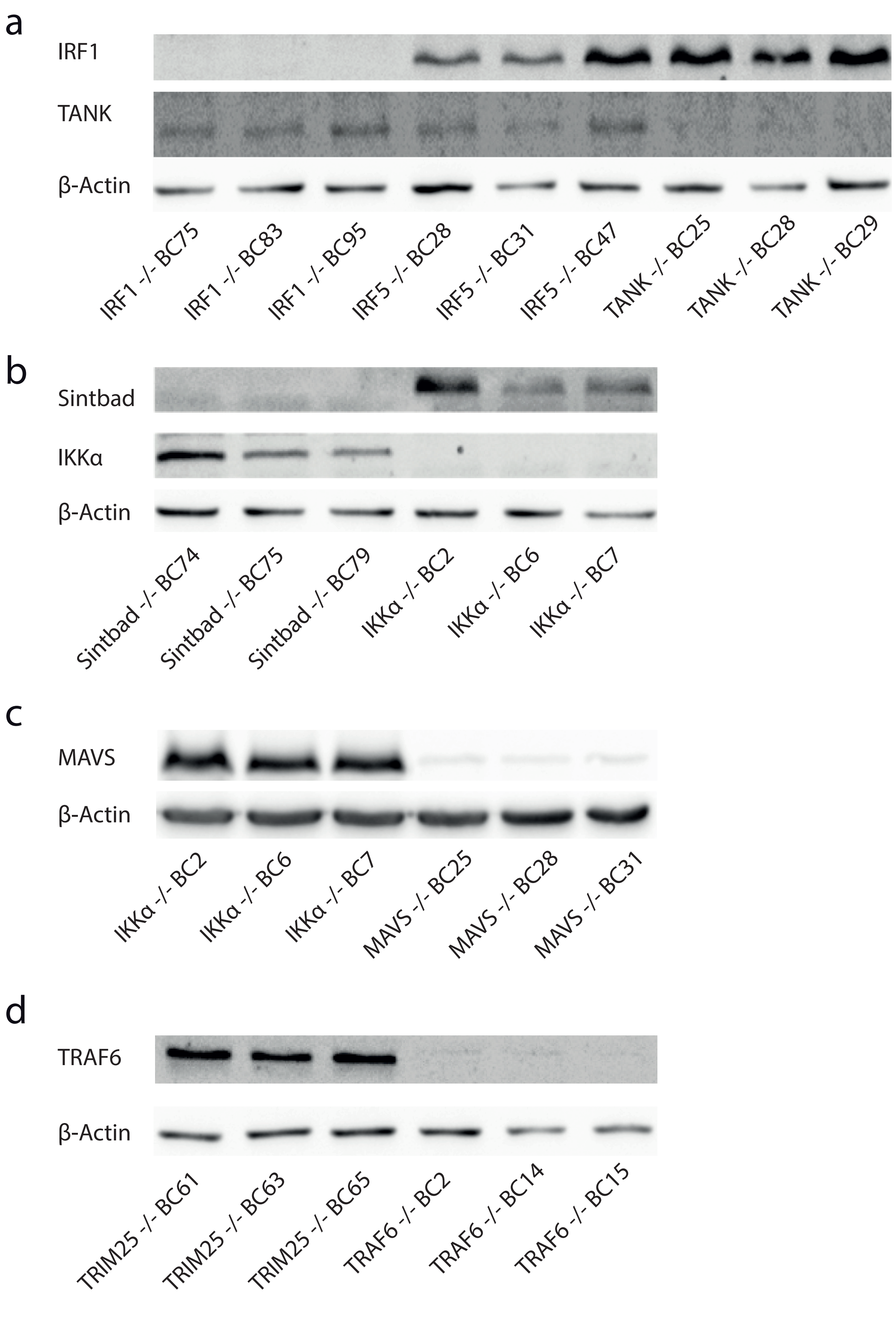

Supplementary Figure S7 | Expression analysis of six different target genes by immunoblot. Lysates of knockout cell clones were probed for β-Actin, (**a**) IRF1 and TANK, (**b**) Sintbad and IKKα, (**c**) MAVS or (**d**) TRAF6. Knockout cell clones with different genotypes served as wildtype controls. The genotype is indicated on the bottom of the blots, BC indicates the barcode combination that was used for deep sequencing to identify individual clones.

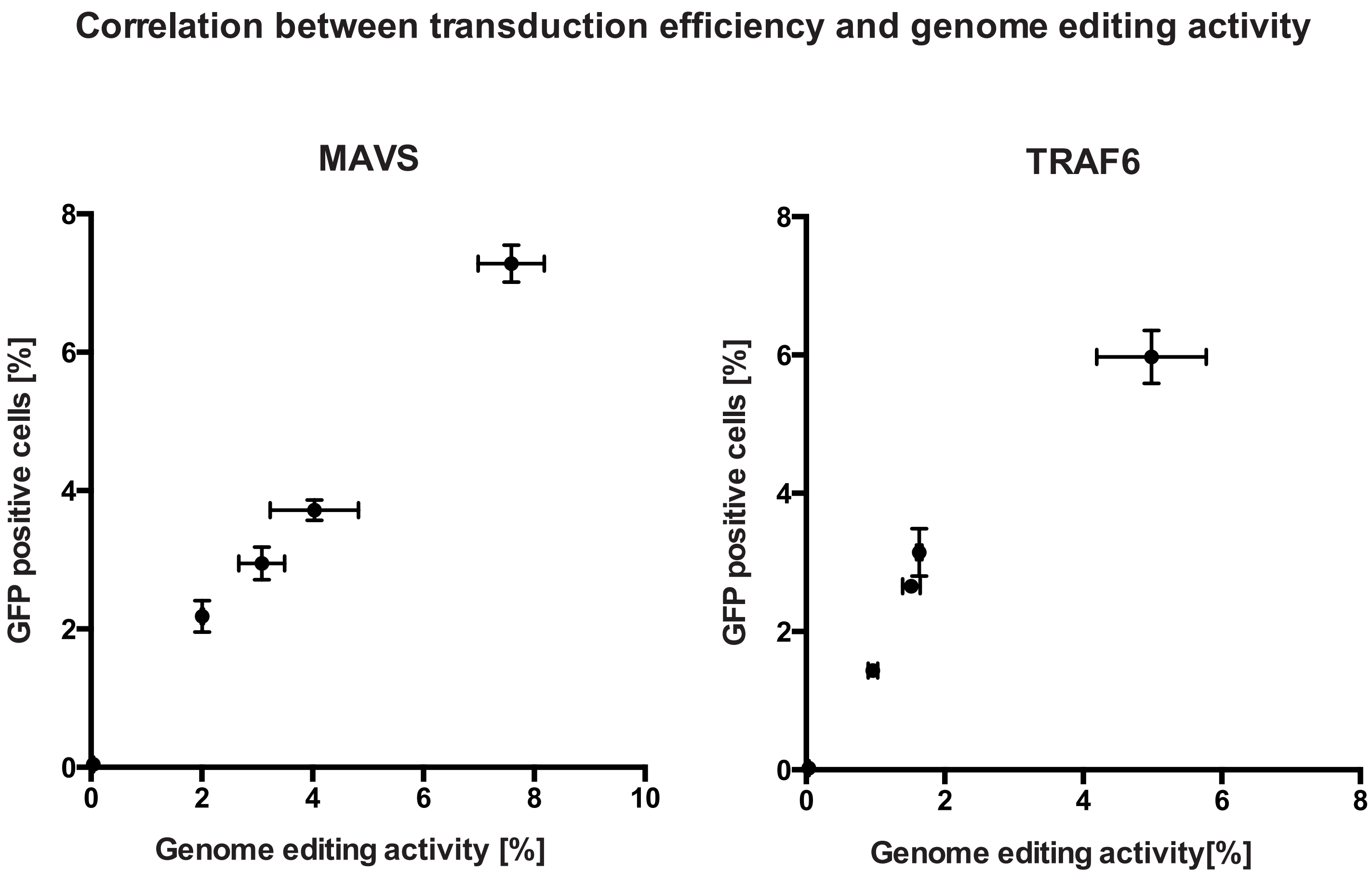

Supplementary Figure S8 | Correlation between transduction efficiency and genome editing activity. HEK 293T cells stably expressing Cas9 were transduced with different amounts of lentivirus containing a library plasmid expressing the sgRNA for MAVS and TRAF6. Depicted are plots that show the correlation between the transduction efficiency of the lentiviruses and the genome editing activity in HEK293T cells. Shown are mean values of three independent replicates +/-SEM.

Supplementary Notes

Orthogonal mixing and clone calling considerations

The orthogonal mixing strategy we applied allows allocating library member sequences within a large number of library clones while requiring a significantly lower number of PCR reactions and barcoding primer combinations in comparison to the number of clones to be analyzed (Figure 3).

Considering one set of 32 clone plates, an exemplary library sequence found only in position A1 of the respective position-based mixing plate and only in pool I on the group-based mixing plate can be clearly allocated to position A1 on plate 1 of the set. However, when considering a library sequence present twice within a set of 32 clone plates, it depends on the localization of the duplicates if the position of both clones can be unambiguously recovered from the data. For example, if the duplicates are located on a single plate of the set at two different positions, a reliable call is possible. However, considering a library sequence present on plate 1 at position A1 as well as on plate 2 at position A2, the respective sequence would appear twice in the set of the position-based mix plates and twice in the set of the group-based mix plates. As such, it is impossible to conclude from the sequencing data if the sequence is located at (plate 1 A1) and (plate 2 A2) or, alternatively, at (plate 1 A2) and (plate 2 A1). Therefore, any clone calling algorithm must reject calling these positions. Consequently, sequence duplicates do not only reduce the coverage of a library by reducing the total number of individual clones being picked, but also do so by reducing the number of unambiguously allocatable sequences if the duplicates appear within qualifying relative locations among the clone plates.

In the clone library picked from pool A, we observed a high number of clonal duplicates among 96 clones picked from a single transformation reaction streaked on a single agar plate. This is in line with our observation that after 29,568 picks out of a library of 19,956 member sequences at a per-clone fidelity of 54.2%, only 39.4% of library sequences were recovered, while a random clone picking simulation predicts 55.2% of the library to be covered according to the equation:

$$\textit{Expected Coverage} = 1 - ((1-(1/\textit{Library Sequences}))\ \char`^\ (\textit{Pick Number} * \textit{Fidelity}))$$

A likely explanation for the obvious phenomenon of clonal duplicates within clone sets or on single clone picking plates is that during recovery from transformation before agar plate streaking, bacterial clones might have divided one or two times. Indeed, when we omitted the 1 h recovery step prior to plating the transformed bacteria (pool B), a much lower number of clonal duplicates was observed.

The sgRNA^{KOLIBRY} target site selection criteria

After extracting a list of all annotated human mRNA sequences from the current NCBI Reference Sequence Database, every mRNA sequence was scanned for start codons by the sequence *ATG*. At every start codon, the length of a possible open reading frame (ORF) was determined by seeking the first cognate stop codon with the sequence *TGA*, *TAG* or *TAA* shifted downstream by 3 x n bases. Of all ORFs identified in a given mRNA, only the longest ORF was considered for further analyses. To rule out the possibility that an alternative, cognate start codon in the same reading frame renders CRISPR targeting inefficient for disrupting functional gene expression, the first 80 bases of the longest identified ORF were scanned for the presence of secondary in-frame start codons. Upon encounter of such, the

ORF boundary was shifted to that additional, upstream *ATG*. Next, possible CRISPR target sites were identified by searching the sequence 5’-N_{21}-GG-3’ or 5’-CC-N_{21}-3’ with the limitation that the target site has to match the human genome (UCSC hg19 assembly). To avoid the possibility of expressing truncated proteins that still retain the full or partial functions of the full-length protein, only target sites located within the first half of the ORF were considered. This measure also increases the likelihood of subjecting the frame-shifted mRNA molecule to nonsense mediated mRNA decay. For all chosen target sites an activity prediction score was calculated based on the base preferences at position 14, 15 (A or T) and 18 (C or G) we observed. We focused on these three positions for their location in the PAM-proximal seed region and as a trade-off between enhancing sgRNA activity and limiting the possible targeting space. Previous studies had identified that point mutations within the PAM-proximal seed region of 6-13bp strongly impact on DNA cleavage activity by the CRISPR:Cas9 complex. Therefore, to limit off-target effects, we favored target sites for which the PAM-proximal 13bp sequence was most unique in the human genome. Considering these criteria, target sites were identified in a hierarchical selection process as depicted in Supplementary Figure 3. The best target site per target gene was then chosen for the genome-wide sgRNA library, whereas the characteristics of these target sites are depicted in Supplementary Figure 4.

Supplementary Methods

CRISPR sgRNA assembly

Backbone preparation

10 µl of the sgRNA entry vector (200 ng/µL in H_2O) were digested with ApaI and SpeI (Fermentas, FastDigest) at 37 °C for 2 h.

Vector Digestion Mix:

2 µl	10 x Buffer FastDigest
10 µl	Plasmid Prep 200 ng/µl
1 µl	FastDigest ApaI
1 µl	FastDigest SpeI
6 µl	H_2O

After 2h, any undigested vector was cleaved by EcoRV (Fermentas, FastDigest) at 37 °C for 1h.

1 µl	FastDigest EcoRV

The digestion mix was separated on an agarose gel (1 %), the cleaved product was purified using the innuPrep gel extraction kit (Analytik Jena) and eluted in 15 µl H_2O (typical concentration: 70 ng/µl).

Chewback reaction

The digested and purified entry vector was chewed using T4 DNA polymerase (Enzymatics).

Chewback Reaction Mix:

10 µl	10 x NEB2
10 µl	Vector 70 ng/µl
1 µl	BSA 10 mg/ml
1 µl	dTTP 100 mM
74.66 µl	H_2O
3.33 µl	T4 DNA polymerase 3 U/µl

The reaction was incubated at 27 °C for 5 min, put on ice and subsequently inactivated at 75°C for 20min. Next, the chewed vector was diluted with the short universal reverse strand oligo (5'-PHOS-AACGGACTAGCCTTATTTTAACTTGCTATTTCTAGCTCTAAAAC-3', PAGE purified, 100 µM, IDT) to a final buffer concentration of 2 x NEB2.

Vector Dilution Mix:

20 µl	10 x NEB2
10 µl	chewed Vector
0.5 µl	short universal reverse strand oligo 100 µM
69.5 µl	H_2O

Assembly

Gene specific sgRNA oligos (desalted, 100 µM, IDT) were diluted 1:400 in H_2O and next mixed with the Vector dilution and annealed in a PCR cycler (C100, Biorad).

Assembly Mix:

2.5 µL	Vector Dilution Mix
2.5 µL	sgRNA oligo Dilution 0.25 µM

Annealing Program:

Temp	Time	Ramp	
75 °C	2.5 min	0.1 °C/sec	│15 cycles; -1 °C per cycle
60 °C	30 min	0.1 °C/sec	
60 °C	2.5 min	0.1 °C/sec	│35 cycles; -1 °C per cycle
25 °C	∞		

Supplementary Table S1 | 96 individual sgRNA oligonucleotides

Gene	Target site + PAM (5' -> 3')	Species	Oligo (5' -> 3')
AIM2	GCTTTTGGCAAAACGTCTTCAGG	human	GGAAAGGACGAAACACCGCTTTTGGCAAAACGTCTTCGTTTTAGAGCTAGAAATAG CAAGTTAAAATAAGG
IFI16	GGTTTTAAACACCAGCTTGAAGG	human	GGAAAGGACGAAACACCGGTTTTAAACACCAGCTTGAGTTTTAGAGCTAGAAATAG CAAGTTAAAATAAGG
IFI16	TTTTAAATCGTTGCTCAGTAAGG	human	GGAAAGGACGAAACACCGTTTAAATCGTTGCTCAGTAGTTTTAGAGCTAGAAATAG CAAGTTAAAATAAGG
IFI16	TCAGCCAGGTCTTCAAGCGTTGG	human	GGAAAGGACGAAACACCGCAGCCAGGTCTTCAAGCGTGTTTTAGAGCTAGAAATAG CAAGTTAAAATAAGG
IFI16	TTCCTCTTTCTTGATAGGGCTGG	human	GGAAAGGACGAAACACCGTCCTCTTTCTTGATAGGGCGTTTTAGAGCTAGAAATAG CAAGTTAAAATAAGG
IFI16	CAAAGGGAGTAAGGTGTCCGAGG	human	GGAAAGGACGAAACACCGAAAGGGAGTAAGGTGTCCGGTTTTAGAGCTAGAAATA GCAAGTTAAAATAAGG
PYHIN1	GCCAGCACGTCCACAGCCATGGG	human	GGAAAGGACGAAACACCGCCAGCACGTCCACAGCCATGTTTTAGAGCTAGAAATAG CAAGTTAAAATAAGG
PYHIN1	TTTTAAATCGTTACTCAGTAAGG	human	GGAAAGGACGAAACACCGTTTAAATCGTTACTCAGTAGTTTTAGAGCTAGAAATAG CAAGTTAAAATAAGG
PYHIN1	TTCTATTAGTTTGCCCAAACCGG	human	GGAAAGGACGAAACACCGTCTATTAGTTTGCCCAAACGTTTTAGAGCTAGAAATAG CAAGTTAAAATAAGG
PYHIN1	TTAGCTGTGAGACGGTTGCTTGG	human	GGAAAGGACGAAACACCGTAGCTGTGAGACGGTTGCTGTTTTAGAGCTAGAAATAG CAAGTTAAAATAAGG
PYHIN1	ACTGGCAGTTGCGTGACGGTTGG	human	GGAAAGGACGAAACACCGCTGGCAGTTGCGTGACGGTGTTTTAGAGCTAGAAATA GCAAGTTAAAATAAGG
MNDA	GGAGTAAACGAAGTGTTGGATGG	human	GGAAAGGACGAAACACCGGAGTAAACGAAGTGTTGGAGTTTTAGAGCTAGAAATA GCAAGTTAAAATAAGG
MNDA	TTCTATTAGTTTGTCTAGACAGG	human	GGAAAGGACGAAACACCGTCTATTAGTTTGTCTAGACGTTTTAGAGCTAGAAATAG CAAGTTAAAATAAGG
MNDA	CAAGAGCAGAGTAAGCCCCCAGG	human	GGAAAGGACGAAACACCGAAGAGCAGAGTAAGCCCCCGTTTTAGAGCTAGAAATA GCAAGTTAAAATAAGG
MNDA	ACTGTCACTGGGTCGTTTTGGGG	human	GGAAAGGACGAAACACCGCTGTCACTGGGTCGTTTTGGTTTTAGAGCTAGAAATAG CAAGTTAAAATAAGG
MNDA	CCACTGTCACTGGGTCGTTTTGG	human	GGAAAGGACGAAACACCGCACTGTCACTGGGTCGTTTGTTTTAGAGCTAGAAATAG CAAGTTAAAATAAGG
TMEM173	GCGGGCCGACCGCATTTGGGAGG	human	GGAAAGGACGAAACACCGCGGGCCGACCGCATTTGGGGTTTTAGAGCTAGAAATA GCAAGTTAAAATAAGG
TMEM173	GGCTCACTGCACCCCGTAGCAGG	human	GGAAAGGACGAAACACCGGCTCACTGCACCCCGTAGCGTTTTAGAGCTAGAAATAG CAAGTTAAAATAAGG
TMEM173	TCCATCCATCCCGTGTCCCAGGG	human	GGAAAGGACGAAACACCGCCATCCATCCCGTGTCCCAGTTTTAGAGCTAGAAATAG CAAGTTAAAATAAGG
TMEM173	AGAGCACACTCTCCGGTACCTGG	human	GGAAAGGACGAAACACCGGAGCACACTCTCCGGTACCGTTTTAGAGCTAGAAATAG CAAGTTAAAATAAGG
TMEM173	CAACAGGGCCCCACGGCGGAGGG	human	GGAAAGGACGAAACACCGAACAGGGCCCCACGGCGGAGTTTTAGAGCTAGAAATA GCAAGTTAAAATAAGG
TMEM173	TACTCCCTCCCAAATGCGGTCGG	human	GGAAAGGACGAAACACCGACTCCCTCCCAAATGCGGTGTTTTAGAGCTAGAAATAG CAAGTTAAAATAAGG
MB21D1	GGCGCCCCTGGCATTCCGTGCGG	human	GGAAAGGACGAAACACCGGCGCCCCTGGCATTCCGTGGTTTTAGAGCTAGAAATAG CAAGTTAAAATAAGG
MB21D1	GGAGACTCGGTGGGATCCATCGG	human	GGAAAGGACGAAACACCGGAGACTCGGTGGGATCCATGTTTTAGAGCTAGAAATA GCAAGTTAAAATAAGG
MB21D1	GAACTTTCCCGCCTTAGGCAGGG	human	GGAAAGGACGAAACACCGAACTTTCCCGCCTTAGGCAGTTTTAGAGCTAGAAATAG CAAGTTAAAATAAGG
MB21D1	GGCCGCCCGTCCGCGCAACTGGG	human	GGAAAGGACGAAACACCGGCCGCCCGTCCGCGCAACTGTTTTAGAGCTAGAAATAG CAAGTTAAAATAAGG
MB21D1	GCCCCAGTTGCGCGGACGGGCGG	human	GGAAAGGACGAAACACCGCCCCAGTTGCGCGGACGGGGTTTTAGAGCTAGAAATA GCAAGTTAAAATAAGG
MB21D1	GCGGGCCCCAGTTGCGCGGACGG	human	GGAAAGGACGAAACACCGCGGGCCCCAGTTGCGCGGAGTTTTAGAGCTAGAAATA GCAAGTTAAAATAAGG
IFIT1	AAATCCCTTCCGCTATAGAATGG	human	GGAAAGGACGAAACACCGAATCCCTTCCGCTATAGAAGTTTTAGAGCTAGAAATAG CAAGTTAAAATAAGG
IFIT1	ACACTCCATTCTATAGCGGAAGG	human	GGAAAGGACGAAACACCGCACTCCATTCTATAGCGGAGTTTTAGAGCTAGAAATAG CAAGTTAAAATAAGG
IFIT1	CATACCCAGCGCTGGATTCAGGG	human	GGAAAGGACGAAACACCGATACCCAGCGCTGGATTCAGTTTTAGAGCTAGAAATAG CAAGTTAAAATAAGG
IFIT1	ATCTCTGCCTATCGCCTGGATGG	human	GGAAAGGACGAAACACCGTCTCTGCCTATCGCCTGGAGTTTTAGAGCTAGAAATAG CAAGTTAAAATAAGG
IFIT1	TTTAAAGCCATCCAGGCGATAGG	human	GGAAAGGACGAAACACCGTTAAAGCCATCCAGGCGATGTTTTAGAGCTAGAAATAG CAAGTTAAAATAAGG

IFIT2	GGGTGGACACGGTTAAAGTGTGG	human	GGAAAGGACGAAACACCGGGTGGACACGGTTAAAGTGGTTTTAGAGCTAGAAATAGCAAGTTAAAATAAGG
IFIT2	GGGTCAATGGCGTTCTGAGATGG	human	GGAAAGGACGAAACACCGGGTCAATGGCGTTCTGAGAGTTTTAGAGCTAGAAATAGCAAGTTAAAATAAGG
IFIT2	AGCCACAATGTGCAACCTACTGG	human	GGAAAGGACGAAACACCGGCCACAATGTGCAACCTACGTTTTAGAGCTAGAAATAGCAAGTTAAAATAAGG
IFIT2	CAGCTTTACGTAAGCATTCCAGG	human	GGAAAGGACGAAACACCGAGCTTTACGTAAGCATTCCGTTTTAGAGCTAGAAATAGCAAGTTAAAATAAGG
IFIT2	ACTCTCAATTCTATAGGGACTGG	human	GGAAAGGACGAAACACCGCTCTCAATTCTATAGGGACGTTTTAGAGCTAGAAATAGCAAGTTAAAATAAGG
IFIT2	AAACCAAAATGAAAGAGCGAAGG	human	GGAAAGGACGAAACACCGAACCAAAATGAAAGAGCGAGTTTTAGAGCTAGAAATAGCAAGTTAAAATAAGG
IFIT3	GGCAATTGCGATGTACCATCTGG	human	GGAAAGGACGAAACACCGGCAATTGCGATGTACCATCGTTTTAGAGCTAGAAATAGCAAGTTAAAATAAGG
IFIT3	CAGCTTGCCGTAAGCATTCCAGG	human	GGAAAGGACGAAACACCGAGCTTGCCGTAAGCATTCCGTTTTAGAGCTAGAAATAGCAAGTTAAAATAAGG
IFIT3	ACCTTGACGTATTGGTTATCAGG	human	GGAAAGGACGAAACACCGCCTTGACGTATTGGTTATCGTTTTAGAGCTAGAAATAGCAAGTTAAAATAAGG
IFIT3	CCAAGAGAACCTTGACGTATTGG	human	GGAAAGGACGAAACACCGCAAGAGAACCTTGACGTATGTTTTAGAGCTAGAAATAGCAAGTTAAAATAAGG
IFIT5	CAAAGAAGAAGTACGAAGCCTGG	human	GGAAAGGACGAAACACCGAAAGAAGAAGTACGAAGCCGTTTTAGAGCTAGAAATAGCAAGTTAAAATAAGG
IFIT5	ACCTTAATATAGCTGTTATCTGG	human	GGAAAGGACGAAACACCGCCTTAATATAGCTGTTATCGTTTTAGAGCTAGAAATAGCAAGTTAAAATAAGG
NLRP3	GCTAATGATCGACTTCAATGGGG	human	GGAAAGGACGAAACACCGCTAATGATCGACTTCAATGGTTTTAGAGCTAGAAATAGCAAGTTAAAATAAGG
NLRP3	GATCGCAGCGAAGATCCACACGG	human	GGAAAGGACGAAACACCGATCGCAGCGAAGATCCACAGTTTTAGAGCTAGAAATAGCAAGTTAAAATAAGG
NLRP3	GTCGTGTGTAGCGTTTGTTGAGG	human	GGAAAGGACGAAACACCGTCGTGTGTAGCGTTTGTTGGTTTTAGAGCTAGAAATAGCAAGTTAAAATAAGG
NLRP3	GATGATGTTGGACTGGGCGTCGG	human	GGAAAGGACGAAACACCGATGATGTTGGACTGGGCGTGTTTTAGAGCTAGAAATAGCAAGTTAAAATAAGG
NLRP3	GCTGGACCATCCTCGGCATGTGG	human	GGAAAGGACGAAACACCGCTGGACCATCCTCGGCATGGTTTTAGAGCTAGAAATAGCAAGTTAAAATAAGG
NLRP3	GGATCTCCACATGCCGAGGATGG	human	GGAAAGGACGAAACACCGGATCTCCACATGCCGAGGAGTTTTAGAGCTAGAAATAGCAAGTTAAAATAAGG
PYCARD	GCGCGCGCGCGACGCCATCCTGG	human	GGAAAGGACGAAACACCGCGCGCGCGCGACGCCATCCGTTTTAGAGCTAGAAATAGCAAGTTAAAATAAGG
PYCARD	GCTGGAGAACCTGACCGCCGAGG	human	GGAAAGGACGAAACACCGCTGGAGAACCTGACCGCCGGTTTTAGAGCTAGAAATAGCAAGTTAAAATAAGG
PYCARD	GGTGAGGTCCAAGGCGTCCATGG	human	GGAAAGGACGAAACACCGGTGAGGTCCAAGGCGTCCAGTTTTAGAGCTAGAAATAGCAAGTTAAAATAAGG
PYCARD	GCTAACGTGCTGCGCGACATGGG	human	GGAAAGGACGAAACACCGCTAACGTGCTGCGCGACATGTTTTAGAGCTAGAAATAGCAAGTTAAAATAAGG
PYCARD	CGACGCCATCCTGGATGCGCTGG	human	GGAAAGGACGAAACACCGGACGCCATCCTGGATGCGCGTTTTAGAGCTAGAAATAGCAAGTTAAAATAAGG
PYCARD	TCTTGAGCTCCTCGGCGGTCAGG	human	GGAAAGGACGAAACACCGCTTGAGCTCCTCGGCGGTCGTTTTAGAGCTAGAAATAGCAAGTTAAAATAAGG
CASP4	GCTCATCCGAATATGGAGGCTGG	human	GGAAAGGACGAAACACCGCTCATCCGAATATGGAGGCGTTTTAGAGCTAGAAATAGCAAGTTAAAATAAGG
CASP5	GGGGCTCACTATGACATCGTGGG	human	GGAAAGGACGAAACACCGGGGCTCACTATGACATCGTGTTTTAGAGCTAGAAATAGCAAGTTAAAATAAGG
CASP5	TTGATCCGTATTAGGTACTAGGG	human	GGAAAGGACGAAACACCGTGATCCGTATTAGGTACTAGTTTTAGAGCTAGAAATAGCAAGTTAAAATAAGG
CASP5	CTCTTTGCGAAAGAATCGCGTGG	human	GGAAAGGACGAAACACCGTCTTTGCGAAAGAATCGCGGTTTTAGAGCTAGAAATAGCAAGTTAAAATAAGG
CASP5	TGGGGCTCACTATGACATCGTGG	human	GGAAAGGACGAAACACCGGGGGCTCACTATGACATCGGTTTTAGAGCTAGAAATAGCAAGTTAAAATAAGG
UNC93B1	GACCTGCTCGGGGTCCCGGACGG	human	GGAAAGGACGAAACACCGACCTGCTCGGGGTCCCGGAGTTTTAGAGCTAGAAATAGCAAGTTAAAATAAGG
UNC93B1	GCACGTTCTTGAGCACGCCCAGG	human	GGAAAGGACGAAACACCGCACGTTCTTGAGCACGCCCGTTTTAGAGCTAGAAATAGCAAGTTAAAATAAGG
UNC93B1	GCTCACCTACGGCGTCTACCTGG	human	GGAAAGGACGAAACACCGCTCACCTACGGCGTCTACCGTTTTAGAGCTAGAAATAGCAAGTTAAAATAAGG
GJA1	ATGGGTGACTGGAGCGCCTTAGG	human	GGAAAGGACGAAACACCGTGGGTGACTGGAGCGCCTTGTTTTAGAGCTAGAAATAGCAAGTTAAAATAAGG
GJA1	TGAGCCAGGTACAAGAGTGTGGG	human	GGAAAGGACGAAACACCGGAGCCAGGTACAAGAGTGTGTTTTAGAGCTAGAAATAGCAAGTTAAAATAAGG
GJA1	ATGGTAAGGTGAAAATGCGAGGG	human	GGAAAGGACGAAACACCGTGGTAAGGTGAAAATGCGAGTTTTAGAGCTAGAAATAGCAAGTTAAAATAAGG

GJC1	GAACACCCAGAAGCGTACATGGG	human	GGAAAGGACGAAACACCGAACACCCAGAAGCGTACATGTTTTAGAGCTAGAAATAGCAAGTTAAAATAAGG
GJC1	GCAAGCCCTATGCAATGCGCTGG	human	GGAAAGGACGAAACACCGCAAGCCCTATGCAATGCGCGTTTTAGAGCTAGAAATAGCAAGTTAAAATAAGG
GJC1	CTTCCTGACTCGCCTGCTAGAGG	human	GGAAAGGACGAAACACCGTTCCTGACTCGCCTGCTAGGTTTTAGAGCTAGAAATAGCAAGTTAAAATAAGG
CASP1	TAACGGAGTCAATCAAAGCTCGG	human	GGAAAGGACGAAACACCGAACGGAGTCAATCAAAGCTGTTTTAGAGCTAGAAATAGCAAGTTAAAATAAGG
CASP1	ATTGACTCCGTTATTCCGAAAGG	human	GGAAAGGACGAAACACCGTTGACTCCGTTATTCCGAAGTTTTAGAGCTAGAAATAGCAAGTTAAAATAAGG
CASP1	TGACTCCGTTATTCCGAAAGGGG	human	GGAAAGGACGAAACACCGGACTCCGTTATTCCGAAAGGTTTTAGAGCTAGAAATAGCAAGTTAAAATAAGG
CASP1	CTGTGCCCCTTTCGGAATAACGG	human	GGAAAGGACGAAACACCGTGTGCCCCTTTCGGAATAAGTTTTAGAGCTAGAAATAGCAAGTTAAAATAAGG
AAVS1	GGGGCCACTAGGGACAGGATTGG	human	GGAAAGGACGAAACACCGGGGCCACTAGGGACAGGATGTTTTAGAGCTAGAAATAGCAAGTTAAAATAAGG
PYHIN1	TTGCCCAAACCGGCATCACCTGG	human	GGAAAGGACGAAACACCGTGCCCAAACCGGCATCACCGTTTTAGAGCTAGAAATAGCAAGTTAAAATAAGG
CASP5	GCAAAGAGTCTACCAAGATCAGG	human	GGAAAGGACGAAACACCGCAAAGAGTCTACCAAGATCGTTTTAGAGCTAGAAATAGCAAGTTAAAATAAGG
IFIT5	GAAGAAGCTCAGAAGTATACAGG	human	GGAAAGGACGAAACACCGAAGAAGCTCAGAAGTATACGTTTTAGAGCTAGAAATAGCAAGTTAAAATAAGG
Mb21d1	GGCGCCGTCGTCCTTCTACGCGG	mouse	GGAAAGGACGAAACACCGGCGCCGTCGTCCTTCTACGGTTTTAGAGCTAGAAATAGCAAGTTAAAATAAGG
Mb21d1	GCGGACGGCTTCTTAGCGCGTGG	mouse	GGAAAGGACGAAACACCGCGGACGGCTTCTTAGCGCGGTTTTAGAGCTAGAAATAGCAAGTTAAAATAAGG
Mb21d1	GTCGGGGCGCGCTTCGCGGACGG	mouse	GGAAAGGACGAAACACCGTCGGGGCGCGCTTCGCGGAGTTTTAGAGCTAGAAATAGCAAGTTAAAATAAGG
Mb21d1	GCAGCTTTCCGCGTGGGCCCTGG	mouse	GGAAAGGACGAAACACCGCAGCTTTCCGCGTGGGCCCGTTTTAGAGCTAGAAATAGCAAGTTAAAATAAGG
Mb21d1	GCGGGCCGCAGCTTTCCGCGTGG	mouse	GGAAAGGACGAAACACCGCGGGCCGCAGCTTTCCGCGGTTTTAGAGCTAGAAATAGCAAGTTAAAATAAGG
Mb21d1	GAAAGCTGCGGCCCGCAAAGGGG	mouse	GGAAAGGACGAAACACCGAAAGCTGCGGCCCGCAAAGGTTTTAGAGCTAGAAATAGCAAGTTAAAATAAGG
Mb21d1	GGGGGCTCGATCGCGGCGGGCGG	mouse	GGAAAGGACGAAACACCGGGGGCTCGATCGCGGCGGGGTTTTAGAGCTAGAAATAGCAAGTTAAAATAAGG
Mb21d1	GGTGGCTCCTGCCGCGTCCATGG	mouse	GGAAAGGACGAAACACCGGTGGCTCCTGCCGCGTCCAGTTTTAGAGCTAGAAATAGCAAGTTAAAATAAGG
Mb21d1	GGCTGGGGCTCCCGTACGGCGGG	mouse	GGAAAGGACGAAACACCGGCTGGGGCTCCCGTACGGCGTTTTAGAGCTAGAAATAGCAAGTTAAAATAAGG
gRNA 16	-	Random	GGAAAGGACGAAACACCGACTGAGAACCCGAAAACAGGTTTTAGAGCTAGAAATAGCAAGTTAAAATAAGG
gRNA 26	-	Random	GGAAAGGACGAAACACCGACTTCGTTTACTCCGAATCGTTTTAGAGCTAGAAATAGCAAGTTAAAATAAGG
gRNA 72	-	Random	GGAAAGGACGAAACACCGAAAAAAGCTCATCCGAATAGTTTTAGAGCTAGAAATAGCAAGTTAAAATAAGG
gRNA 73	-	Random	GGAAAGGACGAAACACCGAGCGCTGACTCCATATCCCGTTTTAGAGCTAGAAATAGCAAGTTAAAATAAGG
gRNA 78	-	Random	GGAAAGGACGAAACACCGAAACCTCTTCTGCAAATCGGTTTTAGAGCTAGAAATAGCAAGTTAAAATAAGG
gRNA 81	-	Random	GGAAAGGACGAAACACCGCCCACCAGCTCGTCCAGCGGTTTTAGAGCTAGAAATAGCAAGTTAAAATAAGG
gRNA 82	-	Random	GGAAAGGACGAAACACCGCGCCCACCAGCTCGTCCAGGTTTTAGAGCTAGAAATAGCAAGTTAAAATAAGG
gRNA 85	-	Random	GGAAAGGACGAAACACCGAGGCCCAGGTAGACGCCGTGTTTTAGAGCTAGAAATAGCAAGTTAAAATAAGG

Supplementary Table S2 | Genome editing activities of 71 CRISPR constructs

Gene	Target site + PAM (5' -> 3')	Background Activity [%]	Activity N1 [%]	Activity N2 [%]	Oligo (5' -> 3')
CASP1	ATTGACTCCGTTATTCCGAAAGG	0,02	62,65	34,41	GGAAAGGACGAAACACCGTTGACTCCGTTATTCCGAAGTTTTAGAGCTAGAAATAGCAAGTTAAAATAAGG
CASP1	CTGTGCCCCTTTCGGAATAACGG	0,09	49,16	25,96	GGAAAGGACGAAACACCGTGTGCCCCTTTCGGAATAAGTTTTAGAGCTAGAAATAGCAAGTTAAAATAAGG
CASP1	TAACGGAGTCAATCAAAGCTCGG	0,09	35,61	25,49	GGAAAGGACGAAACACCGAACGGAGTCAATCAAAGCTGTTTTAGAGCTAGAAATAGCAAGTTAAAATAAGG
CASP1	TGACTCCGTTATTCCGAAAGGGG	0,06	62,71	49,15	GGAAAGGACGAAACACCGGACTCCGTTATTCCGAAAGGTTTTAGAGCTAGAAATAGCAAGTTAAAATAAGG
CASP4	GCTCATCCGAATATGGAGGCTGG	0,00	63,17	79,84	GGAAAGGACGAAACACCGCTCATCCGAATATGGAGGCGTTTTAGAGCTAGAAATAGCAAGTTAAAATAAGG
CASP5	CTCTTTGCGAAAGAATCGCGTGG	0,02	76,72	72,74	GGAAAGGACGAAACACCGTCTTTGCGAAAGAATCGCGGTTTTAGAGCTAGAAATAGCAAGTTAAAATAAGG
CASP5	GGGGCTCACTATGACATCGTGGG	0,00	66,19	44,68	GGAAAGGACGAAACACCGGGGCTCACTATGACATCGTGTTTTAGAGCTAGAAATAGCAAGTTAAAATAAGG
CASP5	TGGGGCTCACTATGACATCGTGG	0,00	85,65	44,03	GGAAAGGACGAAACACCGGGGGCTCACTATGACATCGGTTTTAGAGCTAGAAATAGCAAGTTAAAATAAGG
CASP5	TTGATCCGTATTAGGTACTAGGG	0,03	71,06	53,64	GGAAAGGACGAAACACCGTGATCCGTATTAGGTACTAGTTTTAGAGCTAGAAATAGCAAGTTAAAATAAGG
GJA1	ATGGGTGACTGGAGCGCCTTAGG	0,04	68,52	63,00	GGAAAGGACGAAACACCGTGGGTGACTGGAGCGCCTTGTTTTAGAGCTAGAAATAGCAAGTTAAAATAAGG
GJA1	ATGGTAAGGTGAAAATGCGAGGG	0,09	95,69	51,64	GGAAAGGACGAAACACCGTGGTAAGGTGAAAATGCGAGTTTTAGAGCTAGAAATAGCAAGTTAAAATAAGG
GJA1	TGAGCCAGGTACAAGAGTGTGGG	0,01	87,26	50,26	GGAAAGGACGAAACACCGGAGCCAGGTACAAGAGTGTGTTTTAGAGCTAGAAATAGCAAGTTAAAATAAGG
GJC1	CTTCCTGACTCGCCTGCTAGAGG	0,02	74,96	97,63	GGAAAGGACGAAACACCGTTCCTGACTCGCCTGCTAGGTTTTAGAGCTAGAAATAGCAAGTTAAAATAAGG
GJC1	GAACACCCAGAAGCGTACATGGG	0,01	89,79	48,92	GGAAAGGACGAAACACCGAACACCCAGAAGCGTACATGTTTTAGAGCTAGAAATAGCAAGTTAAAATAAGG
GJC1	GCAAGCCCTATGCAATGCGCTGG	0,00	71,32	56,25	GGAAAGGACGAAACACCGCAAGCCCTATGCAATGCGCGTTTTAGAGCTAGAAATAGCAAGTTAAAATAAGG
IFI16	CAAAGGGAGTAAGGTGTCCGAGG	0,00	84,15	74,55	GGAAAGGACGAAACACCGAAAGGGAGTAAGGTGTCCGGTTTTAGAGCTAGAAATAGCAAGTTAAAATAAGG
IFI16	GGTTTTAAACACCAGCTTGAAGG	0,03	37,19	17,15	GGAAAGGACGAAACACCGGTTTTAAACACCAGCTTGAGTTTTAGAGCTAGAAATAGCAAGTTAAAATAAGG
IFI16	TCAGCCAGGTCTTCAAGCGTTGG	0,05	90,30	56,78	GGAAAGGACGAAACACCGCAGCCAGGTCTTCAAGCGTGTTTTAGAGCTAGAAATAGCAAGTTAAAATAAGG
IFI16	TTCCTCTTTCTTGATAGGGCTGG	0,03	54,95	33,23	GGAAAGGACGAAACACCGTCCTCTTTCTTGATAGGGCGTTTTAGAGCTAGAAATAGCAAGTTAAAATAAGG
IFI16	TTTTAAATCGTTGCTCAGTAAGG	0,00	0,21	0,05	GGAAAGGACGAAACACCGTTTAAATCGTTGCTCAGTAGTTTTAGAGCTAGAAATAGCAAGTTAAAATAAGG
IFIT1	AAATCCCTTCCGCTATAGAATGG	0,72	72,20	78,79	GGAAAGGACGAAACACCGAATCCCTTCCGCTATAGAAGTTTTAGAGCTAGAAATAGCAAGTTAAAATAAGG
IFIT1	ACACTCCATTCTATAGCGGAAGG	0,04	79,08	47,76	GGAAAGGACGAAACACCGCACTCCATTCTATAGCGGAGTTTTAGAGCTAGAAATAGCAAGTTAAAATAAGG
IFIT1	ATCTCTGCCTATCGCCTGGATGG	5,88	64,16	51,66	GGAAAGGACGAAACACCGTCTCTGCCTATCGCCTGGAGTTTTAGAGCTAGAAATAGCAAGTTAAAATAAGG
IFIT1	CATACCCAGCGCTGGATTCAGGG	0,05	66,76	55,20	GGAAAGGACGAAACACCGATACCCAGCGCTGGATTCAGTTTTAGAGCTAGAAATAGCAAGTTAAAATAAGG
IFIT1	TTTAAAGCCATCCAGGCGATAGG	0,00	80,90	54,93	GGAAAGGACGAAACACCGTTAAAGCCATCCAGGCGATGTTTTAGAGCTAGAAATAGCAAGTTAAAATAAGG
IFIT2	AAACCAAAATGAAAGAGCGAAGG	0,00	74,04	79,68	GGAAAGGACGAAACACCGAACCAAAATGAAAGAGCGAGTTTTAGAGCTAGAAATAGCAAGTTAAAATAAGG
IFIT2	ACTCTCAATTCTATAGGGACTGG	0,13	78,45	79,02	GGAAAGGACGAAACACCGCTCTCAATTCTATAGGGACGTTTTAGAGCTAGAAATAGCAAGTTAAAATAAGG
IFIT2	AGCCACAATGTGCAACCTACTGG	0,00	69,58	57,35	GGAAAGGACGAAACACCGGCCACAATGTGCAACCTACGTTTTAGAGCTAGAAATAGCAAGTTAAAATAAGG
IFIT2	CAGCTTTACGTAAGCATTCCAGG	0,00	19,88	27,44	GGAAAGGACGAAACACCGAGCTTTACGTAAGCATTCCGTTTTAGAGCTAGAAATAGCAAGTTAAAATAAGG
IFIT2	GGGTCAATGGCGTTCTGAGATGG	0,01	83,22	76,34	GGAAAGGACGAAACACCGGGTCAATGGCGTTCTGAGAGTTTTAGAGCTAGAAATAGCAAGTTAAAATAAGG
IFIT2	GGGTGGACACGGTTAAAGTGTGG	0,00	79,44	75,16	GGAAAGGACGAAACACCGGGTGGACACGGTTAAAGTGGTTTTAGAGCTAGAAATAGCAAGTTAAAATAAGG
IFIT3	ACCTTGACGTATTGGTTATCAGG	0,03	37,91	57,11	GGAAAGGACGAAACACCGCCTTGACGTATTGGTTATCGTTTTAGAGCTAGAAATAGCAAGTTAAAATAAGG
IFIT3	CAGCTTGCCGTAAGCATTCCAGG	0,01	49,37	40,27	GGAAAGGACGAAACACCGAGCTTGCCGTAAGCATTCCGTTTTAGAGCTAGAAATAGCAAGTTAAAATAAGG

IFIT3	CCAAGAGAACCTTGACGTATTGG	0,00	54,24	45,26	GGAAAGGACGAAACACCGCAAGAGAACCTTGACGTATGTTTTAGAGCTAGAAATAGCAAGTTAAAATAAGG
IFIT3	GGCAATTGCGATGTACCATCTGG	0,00	87,17	57,66	GGAAAGGACGAAACACCGGCAATTGCGATGTACCATCGTTTTAGAGCTAGAAATAGCAAGTTAAAATAAGG
IFIT5	ACCTTAATATAGCTGTTATCTGG	0,04	47,21	55,43	GGAAAGGACGAAACACCGCCTTAATATAGCTGTTATCGTTTTAGAGCTAGAAATAGCAAGTTAAAATAAGG
IFIT5	CAAAGAAGAAGTACGAAGCCTGG	0,01	65,67	47,57	GGAAAGGACGAAACACCGAAAGAAGAAGTACGAAGCCGTTTTAGAGCTAGAAATAGCAAGTTAAAATAAGG
MB21D1	GAACTTTCCCGCCTTAGGCAGGG	0,00	72,06	44,76	GGAAAGGACGAAACACCGAACTTTCCCGCCTTAGGCAGTTTTAGAGCTAGAAATAGCAAGTTAAAATAAGG
MB21D1	GCGGGCCCCAGTTGCGCGGACGG	0,07	47,72	28,55	GGAAAGGACGAAACACCGCCCCAGTTGCGCGGACGGGGTTTTAGAGCTAGAAATAGCAAGTTAAAATAAGG
MB21D1	GGAGACTCGGTGGGATCCATCGG	0,00	34,89	40,15	GGAAAGGACGAAACACCGGAGACTCGGTGGGATCCATGTTTTAGAGCTAGAAATAGCAAGTTAAAATAAGG
MB21D1	GGCCGCCCGTCCGCGCAACTGGG	0,02	25,59	33,00	GGAAAGGACGAAACACCGGCCGCCCGTCCGCGCAACTGTTTTAGAGCTAGAAATAGCAAGTTAAAATAAGG
MB21D1	GGCGCCCCTGGCATTCCGTGCGG	0,04	68,47	50,99	GGAAAGGACGAAACACCGGCGCCCCTGGCATTCCGTGGTTTTAGAGCTAGAAATAGCAAGTTAAAATAAGG
MNDA	CAAGAGCAGAGTAAGCCCCCAGG	0,08	69,13	63,53	GGAAAGGACGAAACACCGAAGAGCAGAGTAAGCCCCCGTTTTAGAGCTAGAAATAGCAAGTTAAAATAAGG
MNDA	CCACTGTCACTGGGTCGTTTTGG	0,08	47,95	45,45	GGAAAGGACGAAACACCGCACTGTCACTGGGTCGTTTGTTTTAGAGCTAGAAATAGCAAGTTAAAATAAGG
MNDA	GGAGTAAACGAAGTGTTGGATGG	0,01	56,49	57,03	GGAAAGGACGAAACACCGGAGTAAACGAAGTGTTGGAGTTTTAGAGCTAGAAATAGCAAGTTAAAATAAGG
MNDA	TTCTATTAGTTTGTCTAGACAGG	0,01	6,28	1,26	GGAAAGGACGAAACACCGTCTATTAGTTTGTCTAGACGTTTTAGAGCTAGAAATAGCAAGTTAAAATAAGG
NLRP3	GATCGCAGCGAAGATCCACACGG	0,02	74,21	63,60	GGAAAGGACGAAACACCGATCGCAGCGAAGATCCACAGTTTTAGAGCTAGAAATAGCAAGTTAAAATAAGG
NLRP3	GATGATGTTGGACTGGGCGTCGG	0,02	85,00	60,68	GGAAAGGACGAAACACCGATGATGTTGGACTGGGCGTGTTTTAGAGCTAGAAATAGCAAGTTAAAATAAGG
NLRP3	GCTAATGATCGACTTCAATGGGG	0,00	76,49	66,03	GGAAAGGACGAAACACCGCTAATGATCGACTTCAATGGTTTTAGAGCTAGAAATAGCAAGTTAAAATAAGG
NLRP3	GCTGGACCATCCTCGGCATGTGG	0,00	57,78	58,11	GGAAAGGACGAAACACCGCTGGACCATCCTCGGCATGGTTTTAGAGCTAGAAATAGCAAGTTAAAATAAGG
NLRP3	GGATCTCCACATGCCGAGGATGG	0,01	63,58	59,67	GGAAAGGACGAAACACCGGATCTCCACATGCCGAGGAGTTTTAGAGCTAGAAATAGCAAGTTAAAATAAGG
NLRP3	GTCGTGTGTAGCGTTTGTTGAGG	0,10	74,03	57,88	GGAAAGGACGAAACACCGTCGTGTGTAGCGTTTGTTGGTTTTAGAGCTAGAAATAGCAAGTTAAAATAAGG
PYCARD	CGACGCCATCCTGGATGCGCTGG	N.D.	96,56	63,72	GGAAAGGACGAAACACCGGACGCCATCCTGGATGCGCGTTTTAGAGCTAGAAATAGCAAGTTAAAATAAGG
PYCARD	GCGCGCGCGCGACGCCATCCTGG	0,38	39,35	40,53	GGAAAGGACGAAACACCGCGCGCGCGCGACGCCATCCGTTTTAGAGCTAGAAATAGCAAGTTAAAATAAGG
PYCARD	GCTAACGTGCTGCGCGACATGGG	0,05	49,76	56,38	GGAAAGGACGAAACACCGCTAACGTGCTGCGCGACATGTTTTAGAGCTAGAAATAGCAAGTTAAAATAAGG
PYCARD	GCTGGAGAACCTGACCGCCGAGG	0,15	81,02	70,75	GGAAAGGACGAAACACCGCTGGAGAACCTGACCGCCGGTTTTAGAGCTAGAAATAGCAAGTTAAAATAAGG
PYCARD	GGTGAGGTCCAAGGCGTCCATGG	0,01	82,00	70,51	GGAAAGGACGAAACACCGGTGAGGTCCAAGGCGTCCAGTTTTAGAGCTAGAAATAGCAAGTTAAAATAAGG
PYCARD	TCTTGAGCTCCTCGGCGGTCAGG	0,03	30,79	23,91	GGAAAGGACGAAACACCGCTTGAGCTCCTCGGCGGTCGTTTTAGAGCTAGAAATAGCAAGTTAAAATAAGG
PYHIN1	ACTGGCAGTTGCGTGACGGTTGG	0,02	53,79	37,39	GGAAAGGACGAAACACCGCTGGCAGTTGCGTGACGGTGTTTTAGAGCTAGAAATAGCAAGTTAAAATAAGG
PYHIN1	GCCAGCACGTCCACAGCCATGGG	0,00	49,92	88,56	GGAAAGGACGAAACACCGCCAGCACGTCCACAGCCATGTTTTAGAGCTAGAAATAGCAAGTTAAAATAAGG
PYHIN1	TTAGCTGTGAGACGGTTGCTTGG	0,03	65,31	52,00	GGAAAGGACGAAACACCGTAGCTGTGAGACGGTTGCTGTTTTAGAGCTAGAAATAGCAAGTTAAAATAAGG
PYHIN1	TTCTATTAGTTTGCCCAAACCGG	0,06	21,58	10,91	GGAAAGGACGAAACACCGTCTATTAGTTTGCCCAAACGTTTTAGAGCTAGAAATAGCAAGTTAAAATAAGG
PYHIN1	TTTTAAATCGTTACTCAGTAAGG	0,01	0,38	0,04	GGAAAGGACGAAACACCGTTTAAATCGTTACTCAGTAGTTTTAGAGCTAGAAATAGCAAGTTAAAATAAGG
TMEM173	AGAGCACACTCTCCGGTACCTGG	0,00	63,20	61,59	GGAAAGGACGAAACACCGGAGCACACTCTCCGGTACCGTTTTAGAGCTAGAAATAGCAAGTTAAAATAAGG
TMEM173	CAACAGGGCCCCACGGCGGAGGG	0,05	16,55	0,37	GGAAAGGACGAAACACCGAACAGGGCCCCACGGCGGAGTTTTAGAGCTAGAAATAGCAAGTTAAAATAAGG
TMEM173	GCGGGCCGACCGCATTTGGGAGG	0,00	74,43	59,19	GGAAAGGACGAAACACCGCGGGCCGACCGCATTTGGGGTTTTAGAGCTAGAAATAGCAAGTTAAAATAAGG
TMEM173	GGCTCACTGCACCCCGTAGCAGG	0,00	75,86	43,58	GGAAAGGACGAAACACCGGCTCACTGCACCCCGTAGCGTTTTAGAGCTAGAAATAGCAAGTTAAAATAAGG
TMEM173	TACTCCCTCCCAAATGCGGTCGG	0,20	73,05	45,63	GGAAAGGACGAAACACCGACTCCCTCCCAAATGCGGTGTTTTAGAGCTAGAAATAGCAAGTTAAAATAAGG

TMEM 173	TCCATCCATCCCGTGTCCCAGGG	0,03	79,71	50,93	GGAAAGGACGAAACACCGCCATCCATCCCGTGTCCCAGTTTTAGAGCTAGAAATAGCAAGTTAAAATAAGG
UNC93 B1	GCACGTTCTTGAGCACGCCCAGG	0,00	87,50	86,35	GGAAAGGACGAAACACCGCACGTTCTTGAGCACGCCCGTTTTAGAGCTAGAAATAGCAAGTTAAAATAAGG
UNC93 B1	GCTCACCTACGGCGTCTACCTGG	0,00	64,09	70,99	GGAAAGGACGAAACACCGCTCACCTACGGCGTCTACCGTTTTAGAGCTAGAAATAGCAAGTTAAAATAAGG

Supplementary Table S5 | Genome editing activity of 43 CRISPR constructs from genome wide library

Gene	Target site + PAM (5' -> 3')	Background Activity [%]	Activity [%]	Oligo (5' -> 3')
AGXT	GTGGACATCGGGGAGCGCATAGG	0,01	64,37	GGAAAGGACGAAACACCGTGGACATCGGGGAGCGCATGTTTTAGAGCTAGAAATAGCAAGTTAAAATAAGG
AIFM1	CCGTGTGCGTCCGAAGCCCGAGG	0,00	68,03	GGAAAGGACGAAACACCGCGTGTGCGTCCGAAGCCCGGTTTTAGAGCTAGAAATAGCAAGTTAAAATAAGG
ARG1	AGGAGCTCCAATAATCCCTATGG	0,04	74,63	GGAAAGGACGAAACACCGGGAGCTCCAATAATCCCTAGTTTTAGAGCTAGAAATAGCAAGTTAAAATAAGG
ARG2	GGATGGAATGCACTCGCGTCTGG	0,00	54,29	GGAAAGGACGAAACACCGGATGGAATGCACTCGCGTCGTTTTAGAGCTAGAAATAGCAAGTTAAAATAAGG
ASL	GAGCTCATTGGTGCAACGGCAGG	0,03	55,62	GGAAAGGACGAAACACCGAGCTCATTGGTGCAACGGCGTTTTAGAGCTAGAAATAGCAAGTTAAAATAAGG
ATF3	GGCAGAAGCACTCACTTCCGAGG	0,01	72,07	GGAAAGGACGAAACACCGGCAGAAGCACTCACTTCCGGTTTTAGAGCTAGAAATAGCAAGTTAAAATAAGG
CTSB	GCCTGCAAGCTTCGATGCACGGG	0,00	68,17	GGAAAGGACGAAACACCGCCTGCAAGCTTCGATGCACGTTTTAGAGCTAGAAATAGCAAGTTAAAATAAGG
CYBB	GTAAGACCTCCGGATGGTTTTGG	0,00	39,34	GGAAAGGACGAAACACCGTAAGACCTCCGGATGGTTTGTTTTAGAGCTAGAAATAGCAAGTTAAAATAAGG
DDX58	GGGTCTTCCGGATATAATCCTGG	0,05	75,93	GGAAAGGACGAAACACCGGGTCTTCCGGATATAATCCGTTTTAGAGCTAGAAATAGCAAGTTAAAATAAGG
DES	GCTGGCTGGACGAGTAGGCCTGG	0,00	85,45	GGAAAGGACGAAACACCGCTGGCTGGACGAGTAGGCCGTTTTAGAGCTAGAAATAGCAAGTTAAAATAAGG
ERBB2	GGTGGGTCTCGGGACTGGCAGGG	0,02	53,67	GGAAAGGACGAAACACCGGTGGGTCTCGGGACTGGCAGTTTTAGAGCTAGAAATAGCAAGTTAAAATAAGG
ERBB3	CAGCATCGCCGGTCACACTCAGG	0,00	64,86	GGAAAGGACGAAACACCGAGCATCGCCGGTCACACTCGTTTTAGAGCTAGAAATAGCAAGTTAAAATAAGG
GGCX	GCTGCTGGCCAGCATGACGTAGG	0,03	66,73	GGAAAGGACGAAACACCGCTGCTGGCCAGCATGACGTGTTTTAGAGCTAGAAATAGCAAGTTAAAATAAGG
IFIH1	GAGGGCTGCCGGTTCTCCGGAGG	0,03	67,69	GGAAAGGACGAAACACCGAGGGCTGCCGGTTCTCCGGGTTTTAGAGCTAGAAATAGCAAGTTAAAATAAGG
CHUK	TAGTTTAGTAGTAGAACCCATGG	0,02	69,89	GGAAAGGACGAAACACCGAGTTTAGTAGTAGAACCCAGTTTTAGAGCTAGAAATAGCAAGTTAAAATAAGG
IKBKG	AATCTGGTTGCTCTGCCGGATGG	0,01	47,79	GGAAAGGACGAAACACCGATCTGGTTGCTCTGCCGGAGTTTTAGAGCTAGAAATAGCAAGTTAAAATAAGG
IRAK4	GATGAACGACCCATTTCTGTTGG	0,02	65,24	GGAAAGGACGAAACACCGATGAACGACCCATTTCTGTGTTTTAGAGCTAGAAATAGCAAGTTAAAATAAGG
IRF1	GGCCCAGCTCCGGAACAAACAGG	0,02	54,56	GGAAAGGACGAAACACCGGCCCAGCTCCGGAACAAACGTTTTAGAGCTAGAAATAGCAAGTTAAAATAAGG
IRF3	GGAGGATTTCGGAATCTTCCAGG	0,00	5,28	GGAAAGGACGAAACACCGGAGGATTTCGGAATCTTCCGTTTTAGAGCTAGAAATAGCAAGTTAAAATAAGG
IRF5	GGGCTTCAGCCGCACGCGGCGGG	0,00	24,17	GGAAAGGACGAAACACCGGGCTTCAGCCGCACGCGGCGTTTTAGAGCTAGAAATAGCAAGTTAAAATAAGG
IRF7	GCAGCCCCACGCGTGCTGTTCGG	0,03	49,20	GGAAAGGACGAAACACCGCAGCCCCACGCGTGCTGTTGTTTTAGAGCTAGAAATAGCAAGTTAAAATAAGG
KDR	GGTAACCAAGGTACTTCGCAGGG	0,05	67,69	GGAAAGGACGAAACACCGGTAACCAAGGTACTTCGCAGTTTTAGAGCTAGAAATAGCAAGTTAAAATAAGG
MAP3K7	GTAAACACCAACTCATTGCGTGG	N.D.	65,96	GGAAAGGACGAAACACCGTAAACACCAACTCATTGCGGTTTTAGAGCTAGAAATAGCAAGTTAAAATAAGG
MAVS	TACTTCATTGCGGCACTGAGGGG	N.D.	41,12	GGAAAGGACGAAACACCGACTTCATTGCGGCACTGAGGTTTTAGAGCTAGAAATAGCAAGTTAAAATAAGG
NAP1	GGCCTATCATGCATATCGAGAGG	0,00	63,49	GGAAAGGACGAAACACCGGCCTATCATGCATATCGAGGTTTTAGAGCTAGAAATAGCAAGTTAAAATAAGG
NCBP2	CGTCCCATTTATGTACCGCATGG	0,04	40,42	GGAAAGGACGAAACACCGGTCCCATTTATGTACCGCAGTTTTAGAGCTAGAAATAGCAAGTTAAAATAAGG
NCF1	CCACCTCCTCGACTTCTTCAAGG	0,04	14,80	GGAAAGGACGAAACACCGCACCTCCTCGACTTCTTCAGTTTTAGAGCTAGAAATAGCAAGTTAAAATAAGG
OASL	GGGCTCCTTCGGGAATGGCACGG	0,29	45,95	GGAAAGGACGAAACACCGGGCTCCTTCGGGAATGGCAGTTTTAGAGCTAGAAATAGCAAGTTAAAATAAGG
OTC	GAATGAAAGTCTCACGGACACGG	N.D.	52,14	GGAAAGGACGAAACACCGAATGAAAGTCTCACGGACAGTTTTAGAGCTAGAAATAGCAAGTTAAAATAAGG
PSPH	CCTACAGGAGCGAAATGTTCAGG	0,00	40,61	GGAAAGGACGAAACACCGCTACAGGAGCGAAATGTTCGTTTTAGAGCTAGAAATAGCAAGTTAAAATAAGG
RIPK1	GCTCCTGGGCGTCATCATAGAGG	7,58	42,83	GGAAAGGACGAAACACCGCTCCTGGGCGTCATCATAGGTTTTAGAGCTAGAAATAGCAAGTTAAAATAAGG

SOLH	GTTGAGGTCGGGCTTGTGCCGGG	0,05	48,85	GGAAAGGACGAAACACCGTTGAGGTCGGGCTTGTGCCGTTTTAGAGCTAGAAATAGCAAGTTAAAATAAGG
TANK	CTCTGTGCCTATACAGTGTACGG	0,02	21,20	GGAAAGGACGAAACACCGTCTGTGCCTATACAGTGTAGTTTTAGAGCTAGAAATAGCAAGTTAAAATAAGG
TBK1	TTCAGATTCTGGTAGTCCATAGG	0,00	82,59	GGAAAGGACGAAACACCGTCAGATTCTGGTAGTCCATGTTTTAGAGCTAGAAATAGCAAGTTAAAATAAGG
TP53	GAGCGCTGCTCAGATAGCGATGG	0,02	64,19	GGAAAGGACGAAACACCGAGCGCTGCTCAGATAGCGAGTTTTAGAGCTAGAAATAGCAAGTTAAAATAAGG
TRAF4	GTGGTGCCCGCATGATGCGGCGG	0,02	56,04	GGAAAGGACGAAACACCGTGGTGCCCGCATGATGCGGGTTTTAGAGCTAGAAATAGCAAGTTAAAATAAGG
TRAF5	CAAACGCATGTGCTCCCGTAAGG	0,01	46,28	GGAAAGGACGAAACACCGAAACGCATGTGCTCCCGTAGTTTTAGAGCTAGAAATAGCAAGTTAAAATAAGG
TRAF7	GCTACAACCGCTTCTCCGGGGGG	0,12	50,79	GGAAAGGACGAAACACCGCTACAACCGCTTCTCCGGGGTTTTAGAGCTAGAAATAGCAAGTTAAAATAAGG
TRIM13	GGAGTGTGCGGAATTCCTTGTGG	0,01	73,04	GGAAAGGACGAAACACCGGAGTGTGCGGAATTCCTTGGTTTTAGAGCTAGAAATAGCAAGTTAAAATAAGG
TRIM25	GTCGCGCCTGGTAGACGGCGCGG	0,00	1,80	GGAAAGGACGAAACACCGTCGCGCCTGGTAGACGGCGGTTTTAGAGCTAGAAATAGCAAGTTAAAATAAGG
TRIM68	CCAGCTGCCAATTAGGCCGCAGG	0,00	30,07	GGAAAGGACGAAACACCGCAGCTGCCAATTAGGCCGCGTTTTAGAGCTAGAAATAGCAAGTTAAAATAAGG
VEZT	ACCACCTCTTCGTCAAACTCCGG	0,05	64,71	GGAAAGGACGAAACACCGCCACCTCTTCGTCAAACTCGTTTTAGAGCTAGAAATAGCAAGTTAAAATAAGG
VKORC1L1	CTTCTCCCGCTCCACGTGGTAGG	0,00	43,62	GGAAAGGACGAAACACCGTTCTCCCGCTCCACGTGGTGTTTTAGAGCTAGAAATAGCAAGTTAAAATAAGG

Supplementary Table S6 | Primer sequences

Primers for amplifying gRNA sequences from orthogonal pools (first PCR):

grna fwd 1	ACACTCTTTCCCTACACGACGCTCTTCCGATCTATTACTCGCTCGGTGCCACTTTTTCAAGTTG
grna fwd 2	ACACTCTTTCCCTACACGACGCTCTTCCGATCTTCCGGAGACTCGGTGCCACTTTTTCAAGTTG
grna fwd 3	ACACTCTTTCCCTACACGACGCTCTTCCGATCTCGCTCATTCTCGGTGCCACTTTTTCAAGTTG
grna fwd 4	ACACTCTTTCCCTACACGACGCTCTTCCGATCTGAGATTCCCTCGGTGCCACTTTTTCAAGTTG
grna fwd 5	ACACTCTTTCCCTACACGACGCTCTTCCGATCTATTCAGAACTCGGTGCCACTTTTTCAAGTTG
grna fwd 6	ACACTCTTTCCCTACACGACGCTCTTCCGATCTGAATTCGTCTCGGTGCCACTTTTTCAAGTTG
grna fwd 7	ACACTCTTTCCCTACACGACGCTCTTCCGATCTCTGAAGCTCTCGGTGCCACTTTTTCAAGTTG
grna fwd 8	ACACTCTTTCCCTACACGACGCTCTTCCGATCTTAATGCGCCTCGGTGCCACTTTTTCAAGTTG
grna fwd 9	ACACTCTTTCCCTACACGACGCTCTTCCGATCTCGGCTATGCTCGGTGCCACTTTTTCAAGTTG
grna fwd 10	ACACTCTTTCCCTACACGACGCTCTTCCGATCTTCCGCGAACTCGGTGCCACTTTTTCAAGTTG
grna fwd 11	ACACTCTTTCCCTACACGACGCTCTTCCGATCTTCTCGCGCCTCGGTGCCACTTTTTCAAGTTG
grna fwd 12	ACACTCTTTCCCTACACGACGCTCTTCCGATCTAGCGATAGCTCGGTGCCACTTTTTCAAGTTG
grna rev	TGACTGGAGTTCAGACGTGTGCTCTTCCGATCTTACGATACAAGGCTGTTAGAGAG

Illumina barcoding primers (second PCR):

Illu fwd 1	AATGATACGGCGACCACCGAGATCTACAC**TATAGCCT**ACACTCTTTCCCTACACGACGCT
Illu fwd 2	AATGATACGGCGACCACCGAGATCTACAC**ATAGAGGC**ACACTCTTTCCCTACACGACGCT
Illu fwd 3	AATGATACGGCGACCACCGAGATCTACAC**CCTATCCT**ACACTCTTTCCCTACACGACGCT
Illu fwd 4	AATGATACGGCGACCACCGAGATCTACAC**GGCTCTGA**ACACTCTTTCCCTACACGACGCT
Illu fwd 5	AATGATACGGCGACCACCGAGATCTACAC**AGGCGAAG**ACACTCTTTCCCTACACGACGCT
Illu fwd 6	AATGATACGGCGACCACCGAGATCTACAC**TAATCTTA**ACACTCTTTCCCTACACGACGCT
Illu fwd 7	AATGATACGGCGACCACCGAGATCTACAC**CAGGACGT**ACACTCTTTCCCTACACGACGCT
Illu fwd 8	AATGATACGGCGACCACCGAGATCTACAC**GTACTGAC**ACACTCTTTCCCTACACGACGCT
Illu fwd 9	AATGATACGGCGACCACCGAGATCTACAC**GCGAAGAT**ACACTCTTTCCCTACACGACGCT
Illu fwd 10	AATGATACGGCGACCACCGAGATCTACAC**CCCTTGTG**ACACTCTTTCCCTACACGACGCT
Illu fwd 11	AATGATACGGCGACCACCGAGATCTACAC**TCGCATCA**ACACTCTTTCCCTACACGACGCT
Illu fwd 12	AATGATACGGCGACCACCGAGATCTACAC**AGAATCAA**ACACTCTTTCCCTACACGACGCT
Illu fwd 13	AATGATACGGCGACCACCGAGATCTACAC**GTTTGAGC**ACACTCTTTCCCTACACGACGCT
Illu fwd 14	AATGATACGGCGACCACCGAGATCTACAC**GACTAACG**ACACTCTTTCCCTACACGACGCT
Illu fwd 15	AATGATACGGCGACCACCGAGATCTACAC**CTCACATA**ACACTCTTTCCCTACACGACGCT
Illu fwd 16	AATGATACGGCGACCACCGAGATCTACAC**CCTGAAAC**ACACTCTTTCCCTACACGACGCT

Illu rev 1	CAAGCAGAAGACGGCATACGAGAT**CGAGTAAT**GTGACTGGAGTTCAGACGTGTGCT
Illu rev 2	CAAGCAGAAGACGGCATACGAGAT**TCTCCGGA**GTGACTGGAGTTCAGACGTGTGCT
Illu rev 3	CAAGCAGAAGACGGCATACGAGAT**AATGAGCG**GTGACTGGAGTTCAGACGTGTGCT
Illu rev 4	CAAGCAGAAGACGGCATACGAGAT**GGAATCTC**GTGACTGGAGTTCAGACGTGTGCT
Illu rev 5	CAAGCAGAAGACGGCATACGAGAT**TTCTGAAT**GTGACTGGAGTTCAGACGTGTGCT
Illu rev 6	CAAGCAGAAGACGGCATACGAGAT**ACGAATTC**GTGACTGGAGTTCAGACGTGTGCT
Illu rev 7	CAAGCAGAAGACGGCATACGAGAT**AGCTTCAG**GTGACTGGAGTTCAGACGTGTGCT
Illu rev 8	CAAGCAGAAGACGGCATACGAGAT**GCGCATTA**GTGACTGGAGTTCAGACGTGTGCT
Illu rev 9	CAAGCAGAAGACGGCATACGAGAT**CATAGCCG**GTGACTGGAGTTCAGACGTGTGCT
Illu rev 10	CAAGCAGAAGACGGCATACGAGAT**TTCGCGGA**GTGACTGGAGTTCAGACGTGTGCT
Illu rev 11	CAAGCAGAAGACGGCATACGAGAT**GCGCGAGA**GTGACTGGAGTTCAGACGTGTGCT
Illu rev 12	CAAGCAGAAGACGGCATACGAGAT**CTATCGCT**GTGACTGGAGTTCAGACGTGTGCT
Illu rev 13	CAAGCAGAAGACGGCATACGAGAT**TGTAGTGC**GTGACTGGAGTTCAGACGTGTGCT
Illu rev 14	CAAGCAGAAGACGGCATACGAGAT**GCGTCGAC**GTGACTGGAGTTCAGACGTGTGCT
Illu rev 15	CAAGCAGAAGACGGCATACGAGAT**GGTCTTCT**GTGACTGGAGTTCAGACGTGTGCT
Illu rev 16	CAAGCAGAAGACGGCATACGAGAT**AAATGTCC**GTGACTGGAGTTCAGACGTGTGCT
Illu rev 17	CAAGCAGAAGACGGCATACGAGAT**GTTGAAAC**GTGACTGGAGTTCAGACGTGTGCT
Illu rev 18	CAAGCAGAAGACGGCATACGAGAT**TCTTTACG**GTGACTGGAGTTCAGACGTGTGCT
Illu rev 19	CAAGCAGAAGACGGCATACGAGAT**ATGCCTGG**GTGACTGGAGTTCAGACGTGTGCT
Illu rev 20	CAAGCAGAAGACGGCATACGAGAT**CAATAAGG**GTGACTGGAGTTCAGACGTGTGCT
Illu rev 21	CAAGCAGAAGACGGCATACGAGAT**CGCCGTAA**GTGACTGGAGTTCAGACGTGTGCT
Illu rev 22	CAAGCAGAAGACGGCATACGAGAT**TAAGGCTT**GTGACTGGAGTTCAGACGTGTGCT
Illu rev 23	CAAGCAGAAGACGGCATACGAGAT**TTGCTGCC**GTGACTGGAGTTCAGACGTGTGCT
Illu rev 24	CAAGCAGAAGACGGCATACGAGAT**CTCAATGT**GTGACTGGAGTTCAGACGTGTGCT

Supplementary Table S7 | Vector sequences

pR-U6-gRNA

CTGCAGCCTGAATATGGGCCAAACAGGATATCTGTGGTAAGCAGTTCCTGCCCCGGCTCAGGGCCAAGAACAGAT
GGAACAGCTGAATATGGGCCAAACAGGATATCTGTGGTAAGCAGTTCCTGCCCCGGCTCAGGGCCAAGAACAGAT
GGTCCCCAGATGCGGTCCAGCCCTCAGCAGTTTCTAGAGAACCATCAGATGTTTCCAGGGTGCCCCAAGGACCTG
AAATGACCCTGTGCCTTATTTGAACTAACCAATCAGTTCGCTTCTCGCTTCTGTTCGCGCGCTTCTGCTCCCCGA
GCTCAATAAAAGAGCCCACAACCCCTCACTCGGGGCGCCAGTCCTCCGATTGACTGAGTCGCCCGGGTACCCGTG
TATCCAATAAACCCTCTTGCAGTTGCATCCGACTTGTGGTCTCGCTGTTCCTTGGGAGGGTCTCCTCTGAGTGAT
TGACTACCCGTCAGCGGGGGTCTTTCATTTGGGGGCTCGTCCGGGATCGGGAGACCCCTGCCCAGGGACCACCGA
CCCACCACCGGGAGGTAAGCTGGCCAGCAACTTATCTGTGTCTGTCCGATTGTCTAGTGTCTATGACTGATTTTA
TGCGCCTGCGTCGGTACTAGCTAGTTAGCTAACTAGCTCTGTATCTGGCGGACCCGTGGTGGAACTGACGAGTTC
TGAACACCCGGCCGCAACCCTGGGAGACGTCCCAGGGACTTTGGGGGCCGTTTTTGTGGCCCGACCTGAGGAAGG
GAGTCGATGTGGAATCCGACCCCGTCAGGATATGTGGTTCTGGTAGGAGACGAGAACCTAAAACAGTTCCCGCCT
CCGTCTGAATTTTTGCTTTCGGTTTGGAACCGAAGCCGCGCGTCTTGTCTGCTGCAGCATCGTTCTGTGTTGTCT
CTGTCTGACTGTGTTTCTGTATTTGTCTGAAAATTAGGGCCAGACTGTTACCACTCCCTTAAGTTTGACCTTAGA
TCACTGGAAAGATGTCGAGCGGCTCGCTCACAACCAGTCGGTAGATGTCAAGAAGAGACGTTGGGTTACCTTCTG
CTCTGCAGAATGGCCAACCTTTAACGTCGGATGGCCGCGAGACGGCACCTTTAACCGAGACCTCATCACCCAGGT
TAAGATCAAGGTCTTTTCACCTGGCCCGCATGGACACCCAGACCAGGTCCCCTACATCGTGACCTGGGAAGCCTT
GGCTTTTGACCCCCCTCCCTGGGTCAAGCCCTTTGTACACCCTAAGCCTCCGCCTCCTCTTCTTCCATCCGCGCC
GTCTCTCCCCCTTGAACCTCCTCTTTCGACCCCGCCTCAATCCTCCCTTTATCCAGCCCTCACTCCTTCTCTAGG
CGCCGGCCGGATCCGAGGGCCTATTTCCCATGATTCCTTCATATTTGCATATACGATACAAGGCTGTTAGAGAGA
TAATTGGAATTAATTTGACTGTAAACACAAAGATATTAGTACAAAATACGTGACGTAGAAAGTAATAATTTCTTG
GGTAGTTTGCAGTTTTAAAATTATGTTTTAAAATGGACTATCATATGCTTACCGTAACTTGAAAGTATTTCGATT
TCTTGGCTTTATATATCTTGTGGAAAGGACGAAACACCGGGCCCTCGATATCGACTAGTCCGTTATCAACTTGAA
AAAGTGGCACCGAGTCGGTGCTTTTTTTCGAATTCGCCAGCACAGTGGTCGACCCTGTGGAATGTGTGTCAGTTA
GGGTGTGGAAAGTCCCCAGGCTCCCCAGCAGGCAGAAGTATGCAAAGCATGCATCTCAATTAGTCAGCAACCAGG
TGTGGAAAGTCCCCAGGCTCCCCAGCAGGCAGAAGTATGCAAAGCATGCATCTCAATTAGTCAGCAACCATAGTC
CCGCCCCTAACTCCGCCCATCCCGCCCCTAACTCCGCCCAGTTCCGCCCATTCTCCGCCCCATGGCTGACTAATT
TTTTTTATTTATGCAGAGGCCGAGGCCGCCTCGGCCTCTGAGCTATTCCAGAAGTAGTGAGGAGGCTTTTTTGGA
GGCCTAGGCTTTTGCAAAAAGCTTACCATGACCGAGTACAAGCCCACGGTGCGCCTCGCCACCCGCGACGACGTC
CCCAGGGCCGTACGCACCCTCGCCGCCGCGTTCGCCGACTACCCCGCCACGCGCCACACCGTCGATCCGGACCGC
CACATCGAGCGGGTCACCGAGCTGCAAGAACTCTTCCTCACGCGCGTCGGGCTCGACATCGGCAAGGTGTGGGTC
GCGGACGACGGCGCCGCGGTGGCGGTCTGGACCACGCCGGAGAGCGTCGAAGCGGGGGCGGTGTTCGCCGAGATC
GGCCCGCGCATGGCCGAGTTGAGCGGTTCCCGGCTGGCCGCGCAGCAACAGATGGAAGGCCTCCTGGCGCCGCAC
CGGCCCAAGGAGCCCGCGTGGTTCCTGGCCACCGTCGGCGTCTCGCCCGACCACCAGGGCAAGGGTCTGGGCAGC
GCCGTCGTGCTCCCCGGAGTGGAGGCGGCCGAGCGCGCCGGGGTGCCCGCCTTCCTGGAGACCTCCGCGCCCCGC
AACCTCCCCTTCTACGAGCGGCTCGGCTTCACCGTCACCGCCGACGTCGAGTGCCCGAAGGACCGCGCGACCTGG
TGCATGACCCGCAAGCCCGGTGCCTGACGCCCGCCCCACGACCCGCAGCGCCCGACCGAAAGGAGCGCACGACCC
CATGCATCGATAAAATAAAAGATTTTATTTAGTCTCCAGAAAAAGGGGGGAATGAAAGACCCCACCTGTAGGTTT
GGCAAGCTAGCTTAAGTAACGCCATTTTGCAAGGCATGGAAAAATACATAACTGAGAATAGAGAAGTTCAGATCA
AGGTCAGGAACAGATGGAACAGCTGAATATGGGCCAAACAGGATATCTGTGGTAAGCAGTTCCTGCCCCGGCTCA
GGGCCAAGAACAGATGGAACAGCTGAATATGGGCCAAACAGGATATCTGTGGTAAGCAGTTCCTGCCCCGGCTCA
GGGCCAAGAACAGATGGTCCCCAGATGCGGTCCAGCCCTCAGCAGTTTCTAGAGAACCATCAGATGTTTCCAGGG
TGCCCCAAGGACCTGAAATGACCCTGTGCCTTATTTGAACTAACCAATCAGTTCGCTTCTCGCTTCTGTTCGCGC
GCTTCTGCTCCCCGAGCTCAATAAAAGAGCCCACAACCCCTCACTCGGGGCGCCAGTCCTCCGATTGACTGAGTC
GCCCGGGTACCCGTGTATCCAATAAACCCTCTTGCAGTTGCATCCGACTTGTGGTCTCGCTGTTCCTTGGGAGGG
TCTCCTCTGAGTGATTGACTACCCGTCAGCGGGGGTCTTTCATTTCCGACTTGTGGTCTCGCTGCCTTGGGAGGG
TCTCCTCTGAGTGATTGACTACCCGTCAGCGGGGGTCTTCACATGCAGCATGTATCAAAATTAATTTGGTTTTTT
TTCTTAAGTATTTACATTAAATGGCCATAGTTGCATTAATGAATCGGCCAACGCGCGGGGAGAGGCGGTTTGCGT
ATTGGCGCTCTTCCGCTTCCTCGCTCACTGACTCGCTGCGCTCGGTCGTTCGGCTGCGGCGAGCGGTATCAGCTC
ACTCAAAGGCGGTAATACGGTTATCCACAGAATCAGGGGATAACGCAGGAAAGAACATGTGAGCAAAAGGCCAGC
AAAAGGCCAGGAACCGTAAAAAGGCCGCGTTGCTGGCGTTTTTCCATAGGCTCCGCCCCCCTGACGAGCATCACA
AAAATCGACGCTCAAGTCAGAGGTGGCGAAACCCGACAGGACTATAAAGATACCAGGCGTTTCCCCCTGGAAGCT
CCCTCGTGCGCTCTCCTGTTCCGACCCTGCCGCTTACCGGATACCTGTCCGCCTTTCTCCCTTCGGGAAGCGTGG
CGCTTTCTCATAGCTCACGCTGTAGGTATCTCAGTTCGGTGTAGGTCGTTCGCTCCAAGCTGGGCTGTGTGCACG
AACCCCCCGTTCAGCCCGACCGCTGCGCCTTATCCGGTAACTATCGTCTTGAGTCCAACCCGGTAAGACACGACT
TATCGCCACTGGCAGCAGCCACTGGTAACAGGATTAGCAGAGCGAGGTATGTAGGCGGTGCTACAGAGTTCTTGA
AGTGGTGGCCTAACTACGGCTACACTAGAAGGACAGTATTTGGTATCTGCGCTCTGCTGAAGCCAGTTACCTTCG

GAAAAAGAGTTGGTAGCTCTTGATCCGGCAAACAAACCACCGCTGGTAGCGGTGGTTTTTTTGTTTGCAAGCAGC
AGATTACGCGCAGAAAAAAAGGATCTCAAGAAGATCCTTTGATCTTTTCTACGGGGTCTGACGCTCAGTGGAACG
AAAACTCACGTTAAGGGATTTTGGTCATGAGATTATCAAAAAGGATCTTCACCTAGATCCTTTTAAATTAAAAAT
GAAGTTTGCGGCCGCAAATCAATCTAAAGTATATATGAGTAAACTTGGTCTGACAGTTACCAATGCTTAATCAGT
GAGGCACCTATCTCAGCGATCTGTCTATTTCGTTCATCCATAGTTGCCTGACTCCCCGTCGTGTAGATAACTACG
ATACGGGAGGGCTTACCATCTGGCCCCAGTGCTGCAATGATACCGCGAGACCCACGCTCACCGGCTCCAGATTTA
TCAGCAATAAACCAGCCAGCCGGAAGGGCCGAGCGCAGAAGTGGTCCTGCAACTTTATCCGCCTCCATCCAGTCT
ATTAATTGTTGCCGGGAAGCTAGAGTAAGTAGTTCGCCAGTTAATAGTTTGCGCAACGTTGTTGCCATTGCTACA
GGCATCGTGGTGTCACGCTCGTCGTTTGGTATGGCTTCATTCAGCTCCGGTTCCCAACGATCAAGGCGAGTTACA
TGATCCCCCATGTTGTGCAAAAAAGCGGTTAGCTCCTTCGGTCCTCCGATCGTTGTCAGAAGTAAGTTGGCCGCA
GTGTTATCACTCATGGTTATGGCAGCACTGCATAATTCTCTTACTGTCATGCCATCCGTAAGATGCTTTTCTGTG
ACTGGTGAGTACTCAACCAAGTCATTCTGAGAATAGTGTATGCGGCGACCGAGTTGCTCTTGCCCGGCGTCAACA
CGGGATAATACCGCGCCACATAGCAGAACTTTAAAAGTGCTCATCATTGGAAAACGTTCTTCGGGGCGAAAACTC
TCAAGGATCTTACCGCTGTTGAGATCCAGTTCGATGTAACCCACTCGTGCACCCAACTGATCTTCAGCATCTTTT
ACTTTCACCAGCGTTTCTGGGTGAGCAAAAACAGGAAGGCAAAATGCCGCAAAAAAGGGAATAAGGGCGACACGG
AAATGTTGAATACTCATACTCTTCCTTTTTCAATATTATTGAAGCATTTATCAGGGTTATTGTCTCATGAGCGGA
TACATATTTGAATGTATTTAGAAAAATAAACAAATAGGGGTTCCGCGCACATTTCCCCGAAAAGTGCCAC

pL-U6-gRNA

TGACATTGATTATTGTCTAGTTATTAATAGTAATCAATTACGGGGTCATTAGTTCATAGCCCATATATGGAGTTC
CGCGTTACATAACTTACGGTAAATGGCCCGCCTGGCTGACCGCCCAACGACCCCCGCCCATTGACGTCAATAATG
ACGTATGTTCCCATAGTAACGCCAATAGGGACTTTCCATTGACGTCAATGGGTGGAGTATTTACGGTAAACTGCC
CACTTGGCAGTACATCAAGTGTATCATATGCCAAGTACGCCCCCTATTGACGTCAATGACGGTAAATGGCCCGCC
TGGCATTATGCCCAGTACATGACCTTATGGGACTTTCCTACTTGGCAGTACATCTACGTATTAGTCATCGCTATT
ACCATGGTGATGCGGTTTTGGCAGTACATCAATGGGCGTGGATAGCGGTTTGACTCACGGGGATTTCCAAGTCTC
CACCCCATTGACGTCAATGGGAGTTTGTTTTGGCACCAAAATCAACGGGACTTTCCAAAATGTCGTAACAACTCC
GCCCCATTGACGCAAATGGGCGGTAGGCGTGTACGGTGGGAGGTCTATATAAGCAGAGCTCTCTGGCTAACTAGA
GAACCCACTGCTTACTGGCTTATCGAAATTGGATCCATGGTGAGCAAGGGCGAGGAGCTGTTCACCGGGGTGGTG
CCCATCCTGGTCGAGCTGGACGGCGACGTAAACGGCCACAAGTTCAGCGTGTCCGGCGAGGGCGAGGGCGATGCC
ACCTACGGCAAGCTGACCCTGAAGTTCATCTGCACCACCGGCAAGCTGCCCGTGCCCTGGCCCACCCTCGTGACC
ACCCTGACCTACGGCGTGCAGTGCTTCAGCCGCTACCCCGACCACATGAAGCAGCACGACTTCTTCAAGTCCGCC
ATGCCCGAAGGCTACGTCCAGGAGCGCACCATCTTCTTCAAGGACGACGGCAACTACAAGACCCGCGCCGAGGTG
AAGTTCGAGGGCGACACCCTGGTGAACCGCATCGAGCTGAAGGGCATCGACTTCAAGGAGGACGGCAACATCCTG
GGGCACAAGCTGGAGTACAACTACAACAGCCACAACGTCTATATCATGGCCGACAAGCAGAAGAACGGCATCAAG
GTGAACTTCAAGATCCGCCACAACATCGAGGACGGCAGCGTGCAGCTCGCCGACCACTACCAGCAGAACACCCCC
ATCGGCGACGGCCCCGTGCTGCTGCCCGACAACCACTACCTGAGCACCCAGTCCGCCCTGAGCAAAGACCCCAAC
GAGAAGCGCGATCACATGGTCCTGCTGGAGTTCGTGACCGCCGCCGGGATCACTCTCGGCATGGACGAGCTGTAC
AAGTAAGGTACCTTTAAGACCAATGACTTACAAGGCAGCTGTAGATCTTAGCCACTTTTTAAAAGAAAAGGGGGG
ACTGGAAGGGCTAATTCACTCCCAACGAAGACAAGATCTGCTTTTTGCTTGTACTGGGTCTCTCTGGTTAGACCA
GATCTGAGCCTGGGAGCTCTCTGGCTAACTAGGGAACCCACTGCTTAAGCCTCAATAAAGCTTGCCTTGAGTGCT
TCAAGTAGTGTGTGCCCGTCTGTTGTGTGACTCTGGTAACTAGAGATCCCTCAGACCCTTTTAGTCAGTGTGGAA
AATCTCTAGCAGTAGTAGTTCATGTCATCTTATTATTCAGTATTTATAACTTGCAAAGAAATGAATATCAGAGAG
TGAGAGGAACTTGTTTATTGCAGCTTATAATGGTTACAAATAAAGCAATAGCATCACAAATTTCACAAATAAAGC
ATTTTTTTCACTGCATTCTAGTTGTGGTTTGTCCAAACTCATCAATGTATCTTATCATGTCTGGCTCTAGCTATC
CCGCCCCTAACTCCGCCCATCCCGCCCCTAACTCCGCCCAGTTCCGCCCATTCTCCGCCCCATGGCTGACTAATT
TTTTTTATTTATGCAGAGGCCGAGGCCGCCTCGGCCTCTGAGCTATTCCAGAAGTAGTGAGGAGGCTTTTTTGGA
GGCCTAGGGACGTACCCAATTCGCCCTATAGTGAGTCGTATTACGCGCGCTCACTGGCCGTCGTTTTACAACGTC
GTGACTGGGAAAACCCTGGCGTTACCCAACTTAATCGCCTTGCAGCACATCCCCCTTTCGCCAGCTGGCGTAATA
GCGAAGAGGCCCGCACCGATCGCCCTTCCCAACAGTTGCGCAGCCTGAATGGCGAATGGGACGCGCCCTGTAGCG
GCGCATTAAGCGCGGCGGGTGTGGTGGTTACGCGCAGCGTGACCGCTACACTTGCCAGCGCCCTAGCGCCCGCTC
CTTTCGCTTTCTTCCCTTCCTTTCTCGCCACGTTCGCCGGCTTTCCCCGTCAAGCTCTAAATCGGGGGCTCCCTT
TAGGGTTCCGATTTAGTGCTTTACGGCACCTCGACCCCAAAAAACTTGATTAGGGTGATGGTTCACGTAGTGGGC
CATCGCCCTGATAGACGGTTTTTCGCCCTTTGACGTTGGAGTCCACGTTCTTTAATAGTGGACTCTTGTTCCAAA
CTGGAACAACACTCAACCCTATCTCGGTCTATTCTTTTGATTTATAAGGGATTTTGCCGATTTCGGCCTATTGGT
TAAAAAATGAGCTGATTTAACAAAAATTTAACGCGAATTTTAACAAAATATTAACGCTTACAATTTAGGTGGCAC
TTTTCGGGGAAATGTGCGCGGAACCCCTATTTGTTTATTTTTCTAAATACATTCAAATATGTATCCGCTCATGAG
ACAATAACCCTGATAAATGCTTCAATAATATTGAAAAAGGAAGAGTATGAGTATTCAACATTTCCGTGTCGCCCT
TATTCCCTTTTTTGCGGCATTTTGCCTTCCTGTTTTTGCTCACCCAGAAACGCTGGTGAAAGTAAAAGATGCTGA
AGATCAGTTGGGTGCACGAGTGGGTTACATCGAACTGGATCTCAACAGCGGTAAGATCCTTGAGAGTTTTCGCCC
CGAAGAACGTTTTCCAATGATGAGCACTTTTAAAGTTCTGCTATGTGGCGCGGTATTATCCCGTATTGACGCCGG

GCAAGAGCAACTCGGTCGCCGCATACACTATTCTCAGAATGACTTGGTTGAGTACTCACCAGTCACAGAAAAGCA
TCTTACGGATGGCATGACAGTAAGAGAATTATGCAGTGCTGCCATAACCATGAGTGATAACACTGCGGCCAACTT
ACTTCTGACAACGATCGGAGGACCGAAGGAGCTAACCGCTTTTTTGCACAACATGGGGGATCATGTAACTCGCCT
TGATCGTTGGGAACCGGAGCTGAATGAAGCCATACCAAACGACGAGCGTGACACCACGATGCCTGTAGCAATGGC
AACAACGTTGCGCAAACTATTAACTGGCGAACTACTTACTCTAGCTTCCCGGCAACAATTAATAGACTGGATGGA
GGCGGATAAAGTTGCAGGACCACTTCTGCGCTCGGCCCTTCCGGCTGGCTGGTTTATTGCTGATAAATCTGGAGC
CGGTGAGCGTGGGTCTCGCGGTATCATTGCAGCACTGGGGCCAGATGGTAAGCCCTCCCGTATCGTAGTTATCTA
CACGACGGGGAGTCAGGCAACTATGGATGAACGAAATAGACAGATCGCTGAGATAGGTGCCTCACTGATTAAGCA
TTGGTAACTGTCAGACCAAGTTTACTCATATATACTTTAGATTGATTTAAAACTTCATTTTTAATTTAAAAGGAT
CTAGGTGAAGATCCTTTTTGATAATCTCATGACCAAAATCCCTTAACGTGAGTTTTCGTTCCACTGAGCGTCAGA
CCCCGTAGAAAAGATCAAAGGATCTTCTTGAGATCCTTTTTTTCTGCGCGTAATCTGCTGCTTGCAAACAAAAAA
ACCACCGCTACCAGCGGTGGTTTGTTTGCCGGATCAAGAGCTACCAACTCTTTTTCCGAAGGTAACTGGCTTCAG
CAGAGCGCAGATACCAAATACTGTTCTTCTAGTGTAGCCGTAGTTAGGCCACCACTTCAAGAACTCTGTAGCACC
GCCTACATACCTCGCTCTGCTAATCCTGTTACCAGTGGCTGCTGCCAGTGGCGATAAGTCGTGTCTTACCGGGTT
GGACTCAAGACGATAGTTACCGGATAAGGCGCAGCGGTCGGGCTGAACGGGGGGTTCGTGCACACAGCCCAGCTT
GGAGCGAACGACCTACACCGAACTGAGATACCTACAGCGTGAGCTATGAGAAAGCGCCACGCTTCCCGAAGGGAG
AAAGGCGGACAGGTATCCGGTAAGCGGCAGGGTCGGAACAGGAGAGCGCACGAGGGAGCTTCCAGGGGGAAACGC
CTGGTATCTTTATAGTCCTGTCGGGTTTCGCCACCTCTGACTTGAGCGTCGATTTTTGTGATGCTCGTCAGGGGG
GCGGAGCCTATGGAAAAACGCCAGCAACGCGGCCTTTTTACGGTTCCTGGCCTTTTGCTGGCCTTTTGCTCACAT
GTTCTTTCCTGCGTTATCCCCTGATTCTGTGGATAACCGTATTACCGCCTTTGAGTGAGCTGATACCGCTCGCCG
CAGCCGAACGACCGAGCGCAGCGAGTCAGTGAGCGAGGAAGCGGAAGAGCGCCCAATACGCAAACCGCCTCTCCC
CGCGCGTTGGCCGATTCATTAATGCAGCTGGCACGACAGGTTTCCCGACTGGAAAGCGGGCAGTGAGCGCAACGC
AATTAATGTGAGTTAGCTCACTCATTAGGCACCCCAGGCTTTACACTTTATGCTTCCGGCTCGTATGTTGTGTGG
AATTGTGAGCGGATAACAATTTCACACAGGAAACAGCTATGACCATGATTACGCCAAGCGCGCAATTAACCCTCA
CTAAAGGGAACAAAAGCTGGAGCTGCAAGCTTAATGTAGTCTTATGCAATACTCTTGTAGTCTTGCAACATGGTA
ACGATGAGTTAGCAACATGCCTTACAAGGAGAGAAAAAGCACCGTGCATGCCGATTGGTGGAAGTAAGGTGGTAC
GATCGTGCCTTATTAGGAAGGCAACAGACGGGTCTGACATGGATTGGACGAACCACTGAATTGCCGCATTGCAGA
GATATTGTATTTAAGTGCCTAGCTCGATACATAAACGGGTCTCTCTGGTTAGACCAGATCTGAGCCTGGGAGCTC
TCTGGCTAACTAGGGAACCCACTGCTTAAGCCTCAATAAAGCTTGCCTTGAGTGCTTCAAGTAGTGTGTGCCCGT
CTGTTGTGTGACTCTGGTAACTAGAGATCCCTCAGACCCTTTTAGTCAGTGTGGAAAATCTCTAGCAGTGGCGCC
CGAACAGGGACTTGAAAGCGAAAGGGAAACCAGAGGAGCTCTCTCGACGCAGGACTCGGCTTGCTGAAGCGCGCA
CGGCAAGAGGCGAGGGGCGGCGACTGGTGAGTACGCCAAAAATTTTGACTAGCGGAGGCTAGAAGGAGAGAGATG
GGTGCGAGAGCGTCAGTATTAAGCGGGGGAGAATTAGATCGCGATGGGAAAAAATTCGGTTAAGGCCAGGGGGAA
AGAAAAAATATAAATTAAAACATATAGTATGGGCAAGCAGGGAGCTAGAACGATTCGCAGTTAATCCTGGCCTGT
TAGAAACATCAGAAGGCTGTAGACAAATACTGGGACAGCTACAACCATCCCTTCAGACAGGATCAGAAGAACTTA
GATCATTATATAATACAGTAGCAACCCTCTATTGTGTGCATCAAAGGATAGAGATAAAAGACACCAAGGAAGCTT
TAGACAAGATAGAGGAAGAGCAAAACAAAAGTAAGACCACCGCACAGCAAGCGGCCGCTGATCTTCAGACCTGGA
GGAGGAGATATGAGGGACAATTGGAGAAGTGAATTATATAAATATAAAGTAGTAAAAATTGAACCATTAGGAGTA
GCACCCACCAAGGCAAAGAGAAGAGTGGTGCAGAGAGAAAAAAGAGCAGTGGGAATAGGAGCTTTGTTCCTTGGG
TTCTTGGGAGCAGCAGGAAGCACTATGGGCGCAGCGTCAATGACGCTGACGGTACAGGCCAGACAATTATTGTCT
GGTATAGTGCAGCAGCAGAACAATTTGCTGAGGGCTATTGAGGCGCAACAGCATCTGTTGCAACTCACAGTCTGG
GGCATCAAGCAGCTCCAGGCAAGAATCCTGGCTGTGGAAAGATACCTAAAGGATCAACAGCTCCTGGGGATTTGG
GGTTGCTCTGGAAAACTCATTTGCACCACTGCTGTGCCTTGGAATGCTAGTTGGAGTAATAAATCTCTGGAACAG
ATTTGGAATCACACGACCTGGATGGAGTGGGACAGAGAAATTAACAATTACACAAGCTTAATACACTCCTTAATT
GAAGAATCGCAAAACCAGCAAGAAAAGAATGAACAAGAATTATTGGAATTAGATAAATGGGCAAGTTTGTGGAAT
TGGTTTAACATAACAAATTGGCTGTGGTATATAAAATTATTCATAATGATAGTAGGAGGCTTGGTAGGTTTAAGA
ATAGTTTTTGCTGTACTTTCTATAGTGAATAGAGTTAGGCAGGGATATTCACCATTATCGTTTCAGACCCACCTC
CCAACCCCGAGGGGACCCGACAGGCCCGAAGGAATAGAAGAAGAAGGTGGAGAGAGAGACAGAGACAGATCCATT
CGATTAGTGAACGGATCTCGACGGTATCGATCACGAGACTAGCCTCGAGCGGCCGCCCCCTTCACCGAGGGCCTA
TTTCCCATGATTCCTTCATATTTGCATATACGATACAAGGCTGTTAGAGAGATAATTGGAATTAATTTGACTGTA
AACACAAAGATATTAGTACAAAATACGTGACGTAGAAAGTAATAATTTCTTGGGTAGTTTGCAGTTTTAAAATTA
TGTTTTAAAATGGACTATCATATGCTTACCGTAACTTGAAAGTATTTCGATTTCTTGGCTTTATATATCTTGTGG
AAAGGACGAAACACCGGGCCCTCGATATCGACTAGTCCGTTATCAACTTGAAAAAGTGGCACCGAGTCGGTGCTT
TTTTTCGATGCGGATACTGCAGACAAATGGCAGTATTCATCCACAATTTTAAAAGAAAAGGGGGGATTGGGGGGT
ACAGTGCAGGGGAAAGAATAGTAGACATAATAGCAACAGACATACAAACTAAAGAATTACAAAAACAAATTACAA
AAATTCAAAATTTTCGGGTTTATTACAGGGACAGCAGAGATCCAGTGGCGCGT

pRZ-CMV-Cas9

GACGGATCGGGAGATCTCCCGATCCCCTATGGTGCACTCTCAGTACAATCTGCTCTGATGCCGCATAGTTAAGCC
AGTATCTGCTCCCTGCTTGTGTGTTGGAGGTCGCTGAGTAGTGCGCGAGCAAAATTTAAGCTACAACAAGGCAAG

GCTTGACCGACAATTGCATGAAGAATCTGCTTAGGGTTAGGCGTTTTGCGCTGCTTCGCGAGTTGACATTGATTA
TTGACTAGTTATTAATAGTAATCAATTACGGGGTCATTAGTTCATAGCCCATATATGGAGTTCCGCGTTACATAA
CTTACGGTAAATGGCCCGCCTGGCTGACCGCCCAACGACCCCCGCCCATTGACGTCAATAATGACGTATGTTCCC
ATAGTAACGCCAATAGGGACTTTCCATTGACGTCAATGGGTGGAGTATTTACGGTAAACTGCCCACTTGGCAGTA
CATCAAGTGTATCATATGCCAAGTACGCCCCCTATTGACGTCAATGACGGTAAATGGCCCGCCTGGCATTATGCC
CAGTACATGACCTTATGGGACTTTCCTACTTGGCAGTACATCTACGTATTAGTCATCGCTATTACCATGGTGATG
CGGTTTTGGCAGTACATCAATGGGCGTGGATAGCGGTTTGACTCACGGGGATTTCCAAGTCTCCACCCCATTGAC
GTCAATGGGAGTTTGTTTTGGCACCAAAATCAACGGGACTTTCCAAAATGTCGTAACAACTCCGCCCCATTGACG
CAAATGGGCGGTAGGCGTGTACGGTGGGAGGTCTATATAAGCAGAGCTCAATAAAAGAGCCCACAACCCCTCACT
CGGCGCGCCAGTCTTCCGATAGACTGCGTCGCCCGGGTACCCGTATTCCCAATAAAGCCTCTTGCTGTTTGCATC
CGAATCGTGGTCTCGCTGTTCCTTGGGAGGGTCTCCTCTGAGTGATTGACTACCCACGACGGGGGTCTTTCATTT
GGGGGCTCGTCCGGGATTTGGAGACCCCTGCCCAGGGACCACCGACCCACCACCGGGAGGTAAGCTGGCCAGCAA
CTTATCTGTGTCTGTCCGATTGTCTAGTGTCTATGTTTGATGTTATGCGCCTGCGTCTGTACTAGTTAGCTAACT
AGCTCTGTATCTGGCGGACCCGTGGTGGAACTGACGAGTTCTGAACACCCGGCCGCAACCCTGGGAGACGTCCCA
GGGACTTTGGGGGCCGTTTTTGTGGCCCGACCTGAGGAAGGGAGTCGATGTGGAATCCGACCCCGTCAGGATATG
TGGTTCTGGTAGGAGACGAGAACCTAAAACAGTTCCCGCCTCCGTCTGAATTTTTGCTTTCGGTTTGGAACCGAA
GCCGCGCGTCTTGTCTGCTGCAGCGCTGCAGCATCGTTCTGTGTTGTCTCTGTCTGACTGTGTTTCTGTATTTGT
CTGAAAATTAGGGCCAGACTGTTACCACTCCCTTAAGTTTGACCTTAGGTCACTGGAAAGATGTCGAGCGGATCG
CTCACAACCAGTCGGTAGATGTCAAGAAGAGACGTTGGGTTACCTTCTGCTCTGCAGAATGGCCAACCTTTAACG
TCGGATGGCCGCGAGACGGCACCTTTAACCGAGACCTCATCACCCAGGTTAAGATCAAGGTCTTTTCACCTGGCC
CGCATGGACACCCAGACCAGGTCCCCTACATCGTGACCTGGGAAGCCTTGGCTTTTGACCCCCCTCCCTGGGTCA
AGCCCTTTGTACACCCTAAGCCTCCGCCTCCTCTTCCTCCATCCGCCCCGTCTCTCCCCCTTGAACCTCCTCGTT
CGACCCCGCCTCGATCCTCCCTTTATCCAGCCCTCACTCCTTCTCTAGGCGCCTCCGGACCACCATGGCCAAGTT
GACCAGTGCCGTTCCGGTGCTCACCGCGCGCGACGTCGCCGGAGCGGTCGAGTTCTGGACCGACCGGCTCGGGTT
CTCCCGGGACTTCGTGGAGGACGACTTCGCCGGTGTGGTCCGGGACGACGTGACCCTGTTCATCAGCGCGGTCCA
GGACCAGGTGGTGCCGGACAACACCCTGGCCTGGGTGTGGGTGCGCGGCCTGGACGAGCTGTACGCCGAGTGGTC
GGAGGTCGTGTCCACGAACTTCCGGGACGCCTCCGGGCCGGCCATGACCGAGATCGGCGAGCAGCCGTGGGGGCG
GGAGTTCGCCCTGCGCGACCCGGCCGGCAACTGCGTGCACTTCGTGGCCGAGGAGCAGGACTGAAGATCCGGCCA
TTAGCCATATTATTCATTGGTTATATAGCATAAATCAATATTGGCTATTGGCCATTGCATACGTTGTATCCATAT
CATAATATGTACATTTATATTGGCTCATGTCCAACATTACCGCCATGTTGACATTGATTATTGACTAGTTATTAA
TAGTAATCAATTACGGGGTCATTAGTTCATAGCCCATATATGGAGTTCCGCGTTACATAACTTACGGTAAATGGC
CCGCCTGGCTGACCGCCCAACGACCCCCGCCCATTGACGTCAATAATGACGTATGTTCCCATAGTAACGCCAATA
GGGACTTTCCATTGACGTCAATGGGTGGAGTATTTACGGTAAACTGCCCACTTGGCAGTACATCAAGTGTATCAT
ATGCCAAGTACGCCCCCTATTGACGTCAATGACGGTAAATGGCCCGCCTGGCATTATGCCCAGTACATGACCTTA
TGGGACTTTCCTACTTGGCAGTACATCTACGTATTAGTCATCGCTATTACCATGGTGATGCGGTTTTGGCAGTAC
ATCAATGGGCGTGGATAGCGGTTTGACTCACGGGGATTTCCAAGTCTCCACCCCATTGACGTCAATGGGAGTTTG
TTTTGGCACCAAAATCAACGGGACTTTCCAAAATGTCGTAACAACTCCGCCCCATTGACGCAAATGGGCGGTAGG
CGTGTACGGTGGGAGGTCTATATAAGCAGAGCTCGTTTAGTGAACCGTCAGATCGCCTGGAGACGCCATCCACGC
TGTTTTGACCTCCATAGAAGACACCGGGACCGATCCAGCCTCCGCGGCCCCAAGCTTCCGCGTACGGCCACCATG
GATTATAAGGATGACGACGATAAGGTGAGCAAGGGCGAGGAGGATAACATGGCCATCATCAAGGAGTTCATGCGC
TTCAAGGTGCACATGGAGGGCTCCGTGAACGGCCACGAGTTCGAGATCGAGGGCGAGGGCGAGGGCCGCCCCTAC
GAGGGCACCCAGACCGCCAAGCTGAAGGTGACCAAGGGTGGCCCCCTGCCCTTCGCCTGGGACATCCTGTCCCCT
CAGTTCATGTACGGCTCCAAGGCCTACGTGAAGCACCCCGCCGACATCCCCGACTACTTGAAGCTGTCCTTCCCC
GAGGGCTTCAAGTGGGAGCGCGTGATGAACTTCGAGGACGGCGGCGTGGTGACCGTGACCCAGGACTCCTCCCTG
CAGGACGGCGAGTTCATCTACAAGGTGAAGCTGCGCGGCACCAACTTCCCCTCCGACGGCCCCGTAATGCAGAAG
AAGACCATGGGCTGGGAGGCCTCCTCCGAGCGGATGTACCCCGAGGACGGCGCCCTGAAGGGCGAGATCAAGCAG
AGGCTGAAGCTGAAGGACGGCGGCCACTACGACGCTGAGGTCAAGACCACCTACAAGGCCAAGAAGCCCGTGCAG
CTGCCCGGCGCCTACAACGTCAACATCAAGTTGGACATCACCTCCCACAACGAGGACTACACCATCGTGGAACAG
TACGAACGCGCCGAGGGCCGCCACTCCACCGGCGGCATGGACGAGCTGTACAAGGTCGCTACCATCGAGGGATCC
GGAGAGGGCAGAGGAAGTCTGCTAACATGCGGTGACGTCGAGGAGAATCCTGGCCCAATGGACAAGAAGTACAGC
ATCGGCCTGGACATCGGCACCAACAGCGTGGGCTGGGCCGTGATCACCGACGAGTACAAGGTGCCCAGCAAGAAG
TTCAAGGTGCTGGGCAACACCGACCGCCACAGCATCAAGAAGAACCTGATCGGCGCCCTGCTGTTCGACAGCGGC
GAGACCGCCGAGGCCACCCGCCTGAAGCGCACCGCCCGCCGCCGCTACACCCGCCGCAAGAACCGCATCTGCTAC
CTGCAGGAGATCTTCAGCAACGAGATGGCCAAGGTGGACGACAGCTTCTTCCACCGCCTGGAGGAGAGCTTCCTG
GTGGAGGAGGACAAGAAGCACGAGCGCCACCCCATCTTCGGCAACATCGTGGACGAGGTGGCCTACCACGAGAAG
TACCCCACCATCTACCACCTGCGCAAGAAGCTGGTGGACAGCACCGACAAGGCCGACCTGCGCCTGATCTACCTG
GCCCTGGCCCACATGATCAAGTTCCGCGGCCACTTCCTGATCGAGGGCGACCTGAACCCCGACAACAGCGACGTG
GACAAGCTGTTCATCCAGCTGGTGCAGACCTACAACCAGCTGTTCGAGGAGAACCCCATCAACGCCAGCGGCGTG
GACGCCAAGGCCATCCTGAGCGCCCGCCTGAGCAAGAGCCGCCGCCTGGAGAACCTGATCGCCCAGCTGCCCGGC
GAGAAGAAGAACGGCCTGTTCGGCAACCTGATCGCCCTGAGCCTGGGCCTGACCCCCAACTTCAAGAGCAACTTC

GACCTGGCCGAGGACGCCAAGCTGCAGCTGAGCAAGGACACCTACGACGACGACCTGGACAACCTGCTGGCCCAG
ATCGGCGACCAGTACGCCGACCTGTTCCTGGCCGCCAAGAACCTGAGCGACGCCATCCTGCTGAGCGACATCCTG
CGCGTGAACACCGAGATCACCAAGGCCCCCCTGAGCGCCAGCATGATCAAGCGCTACGACGAGCACCACCAGGAC
CTGACCCTGCTGAAGGCCCTGGTGCGCCAGCAGCTGCCCGAGAAGTACAAGGAGATCTTCTTCGACCAGAGCAAG
AACGGCTACGCCGGCTACATCGACGGCGGCGCCAGCCAGGAGGAGTTCTACAAGTTCATCAAGCCCATCCTGGAG
AAGATGGACGGCACCGAGGAGCTGCTGGTGAAGCTGAACCGCGAGGACCTGCTGCGCAAGCAGCGCACCTTCGAC
AACGGCAGCATCCCCCACCAGATCCACCTGGGCGAGCTGCACGCCATCCTGCGCCGCCAGGAGGACTTCTACCCC
TTCCTGAAGGACAACCGCGAGAAGATCGAGAAGATCCTGACCTTCCGCATCCCCTACTACGTGGGCCCCCTGGCC
CGCGGCAACAGCCGCTTCGCCTGGATGACCCGCAAGAGCGAGGAGACCATCACCCCCTGGAACTTCGAGGAGGTG
GTGGACAAGGGCGCCAGCGCCCAGAGCTTCATCGAGCGCATGACCAACTTCGACAAGAACCTGCCCAACGAGAAG
GTGCTGCCCAAGCACAGCCTGCTGTACGAGTACTTCACCGTGTACAACGAGCTGACCAAGGTGAAGTACGTGACC
GAGGGCATGCGCAAGCCCGCCTTCCTGAGCGGCGAGCAGAAGAAGGCCATCGTGGACCTGCTGTTCAAGACCAAC
CGCAAGGTGACCGTGAAGCAGCTGAAGGAGGACTACTTCAAGAAGATCGAGTGCTTCGACAGCGTGGAGATCAGC
GGCGTGGAGGACCGCTTCAACGCCAGCCTGGGCACCTACCACGACCTGCTGAAGATCATCAAGGACAAGGACTTC
CTGGACAACGAGGAGAACGAGGACATCCTGGAGGACATCGTGCTGACCCTGACCCTGTTCGAGGACCGCGAGATG
ATCGAGGAGCGCCTGAAGACCTACGCCCACCTGTTCGACGACAAGGTGATGAAGCAGCTGAAGCGCCGCCGCTAC
ACCGGCTGGGGCCGCCTGAGCCGCAAGCTGATCAACGGCATCCGCGACAAGCAGAGCGGCAAGACCATCCTGGAC
TTCCTGAAGAGCGACGGCTTCGCCAACCGCAACTTCATGCAGCTGATCCACGACGACAGCCTGACCTTCAAGGAG
GACATCCAGAAGGCCCAGGTGAGCGGCCAGGGCGACAGCCTGCACGAGCACATCGCCAACCTGGCCGGCAGCCCC
GCCATCAAGAAGGGCATCCTGCAGACCGTGAAGGTGGTGGACGAGCTGGTGAAGGTGATGGGCCGCCACAAGCCC
GAGAACATCGTGATCGAGATGGCCCGCGAGAACCAGACCACCCAGAAGGGCCAGAAGAACAGCCGCGAGCGCATG
AAGCGCATCGAGGAGGGCATCAAGGAGCTGGGCAGCCAGATCCTGAAGGAGCACCCCGTGGAGAACACCCAGCTG
CAGAACGAGAAGCTGTACCTGTACTACCTGCAGAACGGCCGCGACATGTACGTGGACCAGGAGCTGGACATCAAC
CGCCTGAGCGACTACGACGTGGACCACATCGTGCCCCAGAGCTTCCTGAAGGACGACAGCATCGACAACAAGGTG
CTGACCCGCAGCGACAAGAACCGCGGCAAGAGCGACAACGTGCCCAGCGAGGAGGTGGTGAAGAAGATGAAGAAC
TACTGGCGCCAGCTGCTGAACGCCAAGCTGATCACCCAGCGCAAGTTCGACAACCTGACCAAGGCCGAGCGCGGC
GGCCTGAGCGAGCTGGACAAGGCCGGCTTCATCAAGCGCCAGCTGGTGGAGACCCGCCAGATCACCAAGCACGTG
GCCCAGATCCTGGACAGCCGCATGAACACCAAGTACGACGAGAACGACAAGCTGATCCGCGAGGTGAAGGTGATC
ACCCTGAAGAGCAAGCTGGTGAGCGACTTCCGCAAGGACTTCCAGTTCTACAAGGTGCGCGAGATCAACAACTAC
CACCACGCCCACGACGCCTACCTGAACGCCGTGGTGGGCACCGCCCTGATCAAGAAGTACCCCAAGCTGGAGAGC
GAGTTCGTGTACGGCGACTACAAGGTGTACGACGTGCGCAAGATGATCGCCAAGAGCGAGCAGGAGATCGGCAAG
GCCACCGCCAAGTACTTCTTCTACAGCAACATCATGAACTTCTTCAAGACCGAGATCACCCTGGCCAACGGCGAG
ATCCGCAAGCGCCCCCTGATCGAGACCAACGGCGAGACCGGCGAGATCGTGTGGGACAAGGGCCGCGACTTCGCC
ACCGTGCGCAAGGTGCTGAGCATGCCCCAGGTGAACATCGTGAAGAAGACCGAGGTGCAGACCGGCGGCTTCAGC
AAGGAGAGCATCCTGCCCAAGCGCAACAGCGACAAGCTGATCGCCCGCAAGAAGGACTGGGACCCCAAGAAGTAC
GGCGGCTTCGACAGCCCCACCGTGGCCTACAGCGTGCTGGTGGTGGCCAAGGTGGAGAAGGGCAAGAGCAAGAAG
CTGAAGAGCGTGAAGGAGCTGCTGGGCATCACCATCATGGAGCGCAGCAGCTTCGAGAAGAACCCCATCGACTTC
CTGGAGGCCAAGGGCTACAAGGAGGTGAAGAAGGACCTGATCATCAAGCTGCCCAAGTACAGCCTGTTCGAGCTG
GAGAACGGCCGCAAGCGCATGCTGGCCAGCGCCGGCGAGCTGCAGAAGGGCAACGAGCTGGCCCTGCCCAGCAAG
TACGTGAACTTCCTGTACCTGGCCAGCCACTACGAGAAGCTGAAGGGCAGCCCCGAGGACAACGAGCAGAAGCAG
CTGTTCGTGGAGCAGCACAAGCACTACCTGGACGAGATCATCGAGCAGATCAGCGAGTTCAGCAAGCGCGTGATC
CTGGCCGACGCCAACCTGGACAAGGTGCTGAGCGCCTACAACAAGCACCGCGACAAGCCCATCCGCGAGCAGGCC
GAGAACATCATCCACCTGTTCACCCTGACCAACCTGGGCGCCCCCGCCGCCTTCAAGTACTTCGACACCACCATC
GACCGCAAGCGCTACACCAGCACCAAGGAGGTGCTGGACGCCACCCTGATCCACCAGAGCATCACCGGCCTGTAC
GAGACCCGCATCGACCTGAGCCAGCTGGGCGGCGACAGCCGCGCCGACCCCAAGAAGAAGCGCAAGGTGTGAGCG
GCCGCATCGATAAAATAAAAGATTTTATTTAGTCTCCAGAAAAAGGGGGGAATGAAAGACCCCACCTGTAGGTTT
GGCAAGCTAGCTTAAGTAACGCCATTTTGCAAGGCATGGAAAAATACATAACTGAGAATAGAGAAGTTCAGATCA
AGGTCAGGAACAGATGGAACAGCTGAATATGGGCCAAACAGGATATCTGTGGTAAGCAGTTCCTGCCCCGGCTCA
GGGCCAAGAACAGATGGAACAGCTGAATATGGGCCAAACAGGATATCTGTGGTAAGCAGTTCCTGCCCCGGCTCA
GGGCCAAGAACAGATGGTCCCCAGATGCGGTCCAGCCCTCAGCAGTTTCTAGAGAACCATCAGATGTTTCCAGGG
TGCCCCAAGGACCTGAAATGACCCTGTGCCTTATTTGAACTAACCAATCAGTTCGCTTCTCGCTTCTGTTCGCGC
GCTTCTGCTCCCCGAGCTCAATAAAAGAGCCCACAACCCCTCACTCGGGGCGCCAGTCCTCCGATTGACTGAGTC
GCCCGGGTACCCGTGTATCCAATAAACCCTCTTGCAGTTGCATCCGACTTGTGGTCTCGCTGTTCCTTGGGAGGG
TCTCCTCTGAGTGATTGACTACCCGTCAGCGGGGGTCTTTCATTTGGGGGCTCGTCCGGGATCGGGAGACCCCTG
CCCAGGGACCACCGACCCACCACCGGGAGGTAAGCTGGGAACTAATTCTGTGGAATGTGTGTCAGTTAGGGTGTG
GAAAGTCCCCAGGCTCCCCAGCAGGCAGAAGTATGCAAAGCATGCATCTCAATTAGTCAGCAACCAGGTGTGGAA
AGTCCCCAGGCTCCCCAGCAGGCAGAAGTATGCAAAGCATGCATCTCAATTAGTCAGCAACCATAGTCCCGCCCC
TAACTCCGCCCATCCCGCCCCTAACTCCGCCCAGTTCCGCCCATTCTCCGCCCCATGGCTGACTAATTTTTTTTA
TTTATGCAGAGGCCGAGGCCGCCTCTGCCTCTGAGCTATTCCAGAAGTAGTGAGGAGGCTTTTTTGGAGGCCTAG
TACCGTCGACCTCTAGCTAGAGCTTGGCGTAATCATGGTCATAGCTGTTTCCTGTGTGAAATTGTTATCCGCTCA

CAATTCCACACAACATACGAGCCGGAAGCATAAAGTGTAAAGCCTGGGGTGCCTAATGAGTGAGCTAACTCACAT
TAATTGCGTTGCGCTCACTGCCCGCTTTCCAGTCGGGAAACCTGTCGTGCCAGCTGCATTAATGAATCGGCCAAC
GCGCGGGGAGAGGCGGTTTGCGTATTGGGCGCTCTTCCGCTTCCTCGCTCACTGACTCGCTGCGCTCGGTCGTTC
GGCTGCGGCGAGCGGTATCAGCTCACTCAAAGGCGGTAATACGGTTATCCACAGAATCAGGGGATAACGCAGGAA
AGAACATGTGAGCAAAAGGCCAGCAAAAGGCCAGGAACCGTAAAAAGGCCGCGTTGCTGGCGTTTTTCCATAGGC
TCCGCCCCCCTGACGAGCATCACAAAAATCGACGCTCAAGTCAGAGGTGGCGAAACCCGACAGGACTATAAAGAT
ACCAGGCGTTTCCCCCTGGAAGCTCCCTCGTGCGCTCTCCTGTTCCGACCCTGCCGCTTACCGGATACCTGTCCG
CCTTTCTCCCTTCGGGAAGCGTGGCGCTTTCTCAATGCTCACGCTGTAGGTATCTCAGTTCGGTGTAGGTCGTTC
GCTCCAAGCTGGGCTGTGTGCACGAACCCCCCGTTCAGCCCGACCGCTGCGCCTTATCCGGTAACTATCGTCTTG
AGTCCAACCCGGTAAGACACGACTTATCGCCACTGGCAGCAGCCACTGGTAACAGGATTAGCAGAGCGAGGTATG
TAGGCGGTGCTACAGAGTTCTTGAAGTGGTGGCCTAACTACGGCTACACTAGAAGGACAGTATTTGGTATCTGCG
CTCTGCTGAAGCCAGTTACCTTCGGAAAAAGAGTTGGTAGCTCTTGATCCGGCAAACAAACCACCGCTGGTAGCG
GTGGTTTTTTTGTTTGCAAGCAGCAGATTACGCGCAGAAAAAAAGGATCTCAAGAAGATCCTTTGATCTTTTCTA
CGGGGTCTGACGCTCAGTGGAACGAAAACTCACGTTAAGGGATTTTGGTCATGAGATTATCAAAAAGGATCTTCA
CCTAGATCCTTTTAAATTAAAAATGAAGTTTTAAATCAATCTAAAGTATATATGAGTAAACTTGGTCTGACAGTT
ACCAATGCTTAATCAGTGAGGCACCTATCTCAGCGATCTGTCTATTTCGTTCATCCATAGTTGCCTGACTCCCCG
TCGTGTAGATAACTACGATACGGGAGGGCTTACCATCTGGCCCCAGTGCTGCAATGATACCGCGAGACCCACGCT
CACCGGCTCCAGATTTATCAGCAATAAACCAGCCAGCCGGAAGGGCCGAGCGCAGAAGTGGTCCTGCAACTTTAT
CCGCCTCCATCCAGTCTATTAATTGTTGCCGGGAAGCTAGAGTAAGTAGTTCGCCAGTTAATAGTTTGCGCAACG
TTGTTGCCATTGCTACAGGCATCGTGGTGTCACGCTCGTCGTTTGGTATGGCTTCATTCAGCTCCGGTTCCCAAC
GATCAAGGCGAGTTACATGATCCCCCATGTTGTGCAAAAAAGCGGTTAGCTCCTTCGGTCCTCCGATCGTTGTCA
GAAGTAAGTTGGCCGCAGTGTTATCACTCATGGTTATGGCAGCACTGCATAATTCTCTTACTGTCATGCCATCCG
TAAGATGCTTTTCTGTGACTGGTGAGTACTCAACCAAGTCATTCTGAGAATAGTGTATGCGGCGACCGAGTTGCT
CTTGCCCGGCGTCAATACGGGATAATACCGCGCCACATAGCAGAACTTTAAAAGTGCTCATCATTGGAAAACGTT
CTTCGGGGCGAAAACTCTCAAGGATCTTACCGCTGTTGAGATCCAGTTCGATGTAACCCACTCGTGCACCCAACT
GATCTTCAGCATCTTTTACTTTCACCAGCGTTTCTGGGTGAGCAAAAACAGGAAGGCAAAATGCCGCAAAAAAGG
GAATAAGGGCGACACGGAAATGTTGAATACTCATACTCTTCCTTTTTCAATATTATTGAAGCATTTATCAGGGTT
ATTGTCTCATGAGCGGATACATATTTGAATGTATTTAGAAAAATAAACAAATAGGGGTTCCGCGCACATTTCCCC
GAAAAGTGCCACCTGACGTC

pBABE-U6-gRNA-Cas9
CTGCAGCCTGAATATGGCCAAACAGGATATCTGTGGTAAGCAGTTCCTGCCCCGGCTCAGGGCCAAGAACAGAT
GGAACAGCTGAATATGGGCCAAACAGGATATCTGTGGTAAGCAGTTCCTGCCCCGGCTCAGGGCCAAGAACAGAT
GGTCCCCAGATGCGGTCCAGCCCTCAGCAGTTTCTAGAGAACCATCAGATGTTTCCAGGGTGCCCCAAGGACCTG
AAATGACCCTGTGCCTTATTTGAACTAACCAATCAGTTCGCTTCTCGCTTCTGTTCGCGCGCTTCTGCTCCCCGA
GCTCAATAAAAGAGCCCACAACCCCTCACTCGGGGCGCCAGTCCTCCGATTGACTGAGTCGCCCGGGTACCCGTG
TATCCAATAAACCCTCTTGCAGTTGCATCCGACTTGTGGTCTCGCTGTTCCTTGGGAGGGTCTCCTCTGAGTGAT
TGACTACCCGTCAGCGGGGGTCTTTCATTTGGGGGCTCGTCCGGGATCGGGAGACCCCTGCCCAGGGACCACCGA
CCCACCACCGGGAGGTAAGCTGGCCAGCAACTTATCTGTGTCTGTCCGATTGTCTAGTGTCTATGACTGATTTTA
TGCGCCTGCGTCGGTACTAGCTAGTTAGCTAACTAGCTCTGTATCTGGCGGACCCGTGGTGGAACTGACGAGTTC
TGAACACCCGGCCGCAACCCTGGGAGACGTCCCAGGGACTTTGGGGGCCGTTTTTGTGGCCCGACCTGAGGAAGG
GAGTCGATGTGGAATCCGACCCCGTCAGGATATGTGGTTCTGGTAGGAGACGAGAACCTAAAACAGTTCCCGCCT
CCGTCTGAATTTTTGCTTTCGGTTTGGAACCGAAGCCGCGCGTCTTGTCTGCTGCAGCATCGTTCTGTGTTGTCT
CTGTCTGACTGTGTTTCTGTATTTGTCTGAAAATTAGGGCCAGACTGTTACCACTCCCTTAAGTTTGACCTTAGA
TCACTGGAAAGATGTCGAGCGGCTCGCTCACAACCAGTCGGTAGATGTCAAGAAGAGACGTTGGGTTACCTTCTG
CTCTGCAGAATGGCCAACCTTTAACGTCGGATGGCCGCGAGACGGCACCTTTAACCGAGACCTCATCACCCAGGT
TAAGATCAAGGTCTTTTCACCTGGCCCGCATGGACACCCAGACCAGGTCCCCTACATCGTGACCTGGGAAGCCTT
GGCTTTTGACCCCCCTCCCTGGGTCAAGCCCTTTGTACACCCTAAGCCTCCGCCTCCTCTTCTTCCATCCGCGCC
GTCTCTCCCCCTTGAACCTCCTCTTTCGACCCCGCCTCAATCCTCCCTTTATCCAGCCCTCACTCCTTCTCTAGG
CGCCGGCCGGATCCGAGGGCCTATTTCCCATGATTCCTTCATATTTGCATATACGATACAAGGCTGTTAGAGAGA
TAATTGGAATTAATTTGACTGTAAACACAAAGATATTAGTACAAAATACGTGACGTAGAAAGTAATAATTTCTTG
GGTAGTTTGCAGTTTTAAAATTATGTTTTAAAATGGACTATCATATGCTTACCGTAACTTGAAAGTATTTCGATT
TCTTGGCTTTATATATCTTGTGGAAAGGACGAAACACCGGGCCCTCGATATCGACTAGTCCGTTATCAACTTGAA
AAAGTGGCACCGAGTCGGTGCTTTTTTTCGAATTCGCCAGCACAGTGGTCGACGTTGACATTGATTATTGTCTAG
TTATTAATAGTAATCAATTACGGGGTCATTAGTTCATAGCCCATATATGGAGTTCCGCGTTACATAACTTACGGT
AAATGGCCCGCCTGGCTGACCGCCCAACGACCCCCGCCCATTGACGTCAATAATGACGTATGTTCCCATAGTAAC
GCCAATAGGGACTTTCCATTGACGTCAATGGGTGGAGTATTTACGGTAAACTGCCCACTTGGCAGTACATCAAGT
GTATCATATGCCAAGTACGCCCCCTATTGACGTCAATGACGGTAAATGGCCCGCCTGGCATTATGCCCAGTACAT
GACCTTATGGGACTTTCCTACTTGGCAGTACATCTACGTATTAGTCATCGCTATTACCATGGTGATGCGGTTTTG
GCAGTACATCAATGGGCGTGGATAGCGGTTTGACTCACGGGGATTTCCAAGTCTCCACCCCATTGACGTCAATGG

GAGTTTGTTTTGGCACCAAAATCAACGGGACTTTCCAAAATGTCGTAACAACTCCGCCCCATTGACGCAAATGGG
CGGTAGGCGTGTACGGTGGGAGGTCTATATAAGCAGAGCTCGTTTAGTGAACCGTCAGATCGCCTGGAGACGCCA
TCCACGCTGTTTTGACCTCCATAGAAGACACCGGGACCGATCCAGCCTCCGCGGCCCCAAGCTTCCGCGTACGGC
CACCATGGATTATAAGGATGACGACGATAAGGTGAGCAAGGGCGAGGAGGATAACATGGCCATCATCAAGGAGTT
CATGCGCTTCAAGGTGCACATGGAGGGCTCCGTGAACGGCCACGAGTTCGAGATCGAGGGCGAGGGCGAGGGCCG
CCCCTACGAGGGCACCCAGACCGCCAAGCTGAAGGTGACCAAGGGTGGCCCCCTGCCCTTCGCCTGGGACATCCT
GTCCCCTCAGTTCATGTACGGCTCCAAGGCCTACGTGAAGCACCCCGCCGACATCCCCGACTACTTGAAGCTGTC
CTTCCCCGAGGGCTTCAAGTGGGAGCGCGTGATGAACTTCGAGGACGGCGGCGTGGTGACCGTGACCCAGGACTC
CTCCCTGCAGGACGGCGAGTTCATCTACAAGGTGAAGCTGCGCGGCACCAACTTCCCCTCCGACGGCCCCGTAAT
GCAGAAGAAGACCATGGGCTGGGAGGCCTCCTCCGAGCGGATGTACCCCGAGGACGGCGCCCTGAAGGGCGAGAT
CAAGCAGAGGCTGAAGCTGAAGGACGGCGGCCACTACGACGCTGAGGTCAAGACCACCTACAAGGCCAAGAAGCC
CGTGCAGCTGCCCGGCGCCTACAACGTCAACATCAAGTTGGACATCACCTCCCACAACGAGGACTACACCATCGT
GGAACAGTACGAACGCGCCGAGGGCCGCCACTCCACCGGCGGCATGGACGAGCTGTACAAGGTCGCTACCATCGA
GGGATCCGGAGAGGGCAGAGGAAGTCTGCTAACATGCGGTGACGTCGAGGAGAATCCTGGCCCAATGGACAAGAA
GTACAGCATCGGCCTGGACATCGGCACCAACAGCGTGGGCTGGGCCGTGATCACCGACGAGTACAAGGTGCCCAG
CAAGAAGTTCAAGGTGCTGGGCAACACCGACCGCCACAGCATCAAGAAGAACCTGATCGGCGCCCTGCTGTTCGA
CAGCGGCGAGACCGCCGAGGCCACCCGCCTGAAGCGCACCGCCCGCCGCCGCTACACCCGCCGCAAGAACCGCAT
CTGCTACCTGCAGGAGATCTTCAGCAACGAGATGGCCAAGGTGGACGACAGCTTCTTCCACCGCCTGGAGGAGAG
CTTCCTGGTGGAGGAGGACAAGAAGCACGAGCGCCACCCCATCTTCGGCAACATCGTGGACGAGGTGGCCTACCA
CGAGAAGTACCCCACCATCTACCACCTGCGCAAGAAGCTGGTGGACAGCACCGACAAGGCCGACCTGCGCCTGAT
CTACCTGGCCCTGGCCCACATGATCAAGTTCCGCGGCCACTTCCTGATCGAGGGCGACCTGAACCCCGACAACAG
CGACGTGGACAAGCTGTTCATCCAGCTGGTGCAGACCTACAACCAGCTGTTCGAGGAGAACCCCATCAACGCCAG
CGGCGTGGACGCCAAGGCCATCCTGAGCGCCCGCCTGAGCAAGAGCCGCCGCCTGGAGAACCTGATCGCCCAGCT
GCCCGGCGAGAAGAAGAACGGCCTGTTCGGCAACCTGATCGCCCTGAGCCTGGGCCTGACCCCCAACTTCAAGAG
CAACTTCGACCTGGCCGAGGACGCCAAGCTGCAGCTGAGCAAGGACACCTACGACGACGACCTGGACAACCTGCT
GGCCCAGATCGGCGACCAGTACGCCGACCTGTTCCTGGCCGCCAAGAACCTGAGCGACGCCATCCTGCTGAGCGA
CATCCTGCGCGTGAACACCGAGATCACCAAGGCCCCCCTGAGCGCCAGCATGATCAAGCGCTACGACGAGCACCA
CCAGGACCTGACCCTGCTGAAGGCCCTGGTGCGCCAGCAGCTGCCCGAGAAGTACAAGGAGATCTTCTTCGACCA
GAGCAAGAACGGCTACGCCGGCTACATCGACGGCGGCGCCAGCCAGGAGGAGTTCTACAAGTTCATCAAGCCCAT
CCTGGAGAAGATGGACGGCACCGAGGAGCTGCTGGTGAAGCTGAACCGCGAGGACCTGCTGCGCAAGCAGCGCAC
CTTCGACAACGGCAGCATCCCCCACCAGATCCACCTGGGCGAGCTGCACGCCATCCTGCGCCGCCAGGAGGACTT
CTACCCCTTCCTGAAGGACAACCGCGAGAAGATCGAGAAGATCCTGACCTTCCGCATCCCCTACTACGTGGGCCC
CCTGGCCCGCGGCAACAGCCGCTTCGCCTGGATGACCCGCAAGAGCGAGGAGACCATCACCCCCTGGAACTTCGA
GGAGGTGGTGGACAAGGGCGCCAGCGCCCAGAGCTTCATCGAGCGCATGACCAACTTCGACAAGAACCTGCCCAA
CGAGAAGGTGCTGCCCAAGCACAGCCTGCTGTACGAGTACTTCACCGTGTACAACGAGCTGACCAAGGTGAAGTA
CGTGACCGAGGGCATGCGCAAGCCCGCCTTCCTGAGCGGCGAGCAGAAGAAGGCCATCGTGGACCTGCTGTTCAA
GACCAACCGCAAGGTGACCGTGAAGCAGCTGAAGGAGGACTACTTCAAGAAGATCGAGTGCTTCGACAGCGTGGA
GATCAGCGGCGTGGAGGACCGCTTCAACGCCAGCCTGGGCACCTACCACGACCTGCTGAAGATCATCAAGGACAA
GGACTTCCTGGACAACGAGGAGAACGAGGACATCCTGGAGGACATCGTGCTGACCCTGACCCTGTTCGAGGACCG
CGAGATGATCGAGGAGCGCCTGAAGACCTACGCCCACCTGTTCGACGACAAGGTGATGAAGCAGCTGAAGCGCCG
CCGCTACACCGGCTGGGGCCGCCTGAGCCGCAAGCTGATCAACGGCATCCGCGACAAGCAGAGCGGCAAGACCAT
CCTGGACTTCCTGAAGAGCGACGGCTTCGCCAACCGCAACTTCATGCAGCTGATCCACGACGACAGCCTGACCTT
CAAGGAGGACATCCAGAAGGCCCAGGTGAGCGGCCAGGGCGACAGCCTGCACGAGCACATCGCCAACCTGGCCGG
CAGCCCCGCCATCAAGAAGGGCATCCTGCAGACCGTGAAGGTGGTGGACGAGCTGGTGAAGGTGATGGGCCGCCA
CAAGCCCGAGAACATCGTGATCGAGATGGCCCGCGAGAACCAGACCACCCAGAAGGGCCAGAAGAACAGCCGCGA
GCGCATGAAGCGCATCGAGGAGGGCATCAAGGAGCTGGGCAGCCAGATCCTGAAGGAGCACCCCGTGGAGAACAC
CCAGCTGCAGAACGAGAAGCTGTACCTGTACTACCTGCAGAACGGCCGCGACATGTACGTGGACCAGGAGCTGGA
CATCAACCGCCTGAGCGACTACGACGTGGACCACATCGTGCCCCAGAGCTTCCTGAAGGACGACAGCATCGACAA
CAAGGTGCTGACCCGCAGCGACAAGAACCGCGGCAAGAGCGACAACGTGCCCAGCGAGGAGGTGGTGAAGAAGAT
GAAGAACTACTGGCGCCAGCTGCTGAACGCCAAGCTGATCACCCAGCGCAAGTTCGACAACCTGACCAAGGCCGA
GCGCGGCGGCCTGAGCGAGCTGGACAAGGCCGGCTTCATCAAGCGCCAGCTGGTGGAGACCCGCCAGATCACCAA
GCACGTGGCCCAGATCCTGGACAGCCGCATGAACACCAAGTACGACGAGAACGACAAGCTGATCCGCGAGGTGAA
GGTGATCACCCTGAAGAGCAAGCTGGTGAGCGACTTCCGCAAGGACTTCCAGTTCTACAAGGTGCGCGAGATCAA
CAACTACCACCACGCCCACGACGCCTACCTGAACGCCGTGGTGGGCACCGCCCTGATCAAGAAGTACCCCAAGCT
GGAGAGCGAGTTCGTGTACGGCGACTACAAGGTGTACGACGTGCGCAAGATGATCGCCAAGAGCGAGCAGGAGAT
CGGCAAGGCCACCGCCAAGTACTTCTTCTACAGCAACATCATGAACTTCTTCAAGACCGAGATCACCCTGGCCAA
CGGCGAGATCCGCAAGCGCCCCCTGATCGAGACCAACGGCGAGACCGGCGAGATCGTGTGGGACAAGGGCCGCGA
CTTCGCCACCGTGCGCAAGGTGCTGAGCATGCCCCAGGTGAACATCGTGAAGAAGACCGAGGTGCAGACCGGCGG
CTTCAGCAAGGAGAGCATCCTGCCCAAGCGCAACAGCGACAAGCTGATCGCCCGCAAGAAGGACTGGGACCCCAA
GAAGTACGGCGGCTTCGACAGCCCCACCGTGGCCTACAGCGTGCTGGTGGTGGCCAAGGTGGAGAAGGGCAAGAG

CAAGAAGCTGAAGAGCGTGAAGGAGCTGCTGGGCATCACCATCATGGAGCGCAGCAGCTTCGAGAAGAACCCCAT
CGACTTCCTGGAGGCCAAGGGCTACAAGGAGGTGAAGAAGGACCTGATCATCAAGCTGCCCAAGTACAGCCTGTT
CGAGCTGGAGAACGGCCGCAAGCGCATGCTGGCCAGCGCCGGCGAGCTGCAGAAGGGCAACGAGCTGGCCCTGCC
CAGCAAGTACGTGAACTTCCTGTACCTGGCCAGCCACTACGAGAAGCTGAAGGGCAGCCCCGAGGACAACGAGCA
GAAGCAGCTGTTCGTGGAGCAGCACAAGCACTACCTGGACGAGATCATCGAGCAGATCAGCGAGTTCAGCAAGCG
CGTGATCCTGGCCGACGCCAACCTGGACAAGGTGCTGAGCGCCTACAACAAGCACCGCGACAAGCCCATCCGCGA
GCAGGCCGAGAACATCATCCACCTGTTCACCCTGACCAACCTGGGCGCCCCCGCCGCCTTCAAGTACTTCGACAC
CACCATCGACCGCAAGCGCTACACCAGCACCAAGGAGGTGCTGGACGCCACCCTGATCCACCAGAGCATCACCGG
CCTGTACGAGACCCGCATCGACCTGAGCCAGCTGGGCGGCGACAGCCGCGCCGACCCCAAGAAGAAGCGCAAGGT
GTGAATCGATAAAATAAAAGATTTTATTTAGTCTCCAGAAAAAGGGGGGAATGAAAGACCCCACCTGTAGGTTTG
GCAAGCTAGCTTAAGTAACGCCATTTTGCAAGGCATGGAAAAATACATAACTGAGAATAGAGAAGTTCAGATCAA
GGTCAGGAACAGATGGAACAGCTGAATATGGGCCAAACAGGATATCTGTGGTAAGCAGTTCCTGCCCCGGCTCAG
GGCCAAGAACAGATGGAACAGCTGAATATGGGCCAAACAGGATATCTGTGGTAAGCAGTTCCTGCCCCGGCTCAG
GGCCAAGAACAGATGGTCCCCAGATGCGGTCCAGCCCTCAGCAGTTTCTAGAGAACCATCAGATGTTTCCAGGGT
GCCCCAAGGACCTGAAATGACCCTGTGCCTTATTTGAACTAACCAATCAGTTCGCTTCTCGCTTCTGTTCGCGCG
CTTCTGCTCCCCGAGCTCAATAAAAGAGCCCACAACCCCTCACTCGGGGCGCCAGTCCTCCGATTGACTGAGTCG
CCCGGGTACCCGTGTATCCAATAAACCCTCTTGCAGTTGCATCCGACTTGTGGTCTCGCTGTTCCTTGGGAGGGT
CTCCTCTGAGTGATTGACTACCCGTCAGCGGGGGTCTTTCATTTCCGACTTGTGGTCTCGCTGCCTTGGGAGGGT
CTCCTCTGAGTGATTGACTACCCGTCAGCGGGGGTCTTCACATGCAGCATGTATCAAAATTAATTTGGTTTTTTT
TCTTAAGTATTTACATTAAATGGCCATAGTTGCATTAATGAATCGGCCAACGCGCGGGGAGAGGCGGTTTGCGTA
TTGGCGCTCTTCCGCTTCCTCGCTCACTGACTCGCTGCGCTCGGTCGTTCGGCTGCGGCGAGCGGTATCAGCTCA
CTCAAAGGCGGTAATACGGTTATCCACAGAATCAGGGGATAACGCAGGAAAGAACATGTGAGCAAAAGGCCAGCA
AAAGGCCAGGAACCGTAAAAAGGCCGCGTTGCTGGCGTTTTTCCATAGGCTCCGCCCCCCTGACGAGCATCACAA
AAATCGACGCTCAAGTCAGAGGTGGCGAAACCCGACAGGACTATAAAGATACCAGGCGTTTCCCCCTGGAAGCTC
CCTCGTGCGCTCTCCTGTTCCGACCCTGCCGCTTACCGGATACCTGTCCGCCTTTCTCCCTTCGGGAAGCGTGGC
GCTTTCTCATAGCTCACGCTGTAGGTATCTCAGTTCGGTGTAGGTCGTTCGCTCCAAGCTGGGCTGTGTGCACGA
ACCCCCCGTTCAGCCCGACCGCTGCGCCTTATCCGGTAACTATCGTCTTGAGTCCAACCCGGTAAGACACGACTT
ATCGCCACTGGCAGCAGCCACTGGTAACAGGATTAGCAGAGCGAGGTATGTAGGCGGTGCTACAGAGTTCTTGAA
GTGGTGGCCTAACTACGGCTACACTAGAAGGACAGTATTTGGTATCTGCGCTCTGCTGAAGCCAGTTACCTTCGG
AAAAAGAGTTGGTAGCTCTTGATCCGGCAAACAAACCACCGCTGGTAGCGGTGGTTTTTTTGTTTGCAAGCAGCA
GATTACGCGCAGAAAAAAAGGATCTCAAGAAGATCCTTTGATCTTTTCTACGGGGTCTGACGCTCAGTGGAACGA
AAACTCACGTTAAGGGATTTTGGTCATGAGATTATCAAAAAGGATCTTCACCTAGATCCTTTTAAATTAAAAATG
AAGTTTGCGGCCGCAAATCAATCTAAAGTATATATGAGTAAACTTGGTCTGACAGTTACCAATGCTTAATCAGTG
AGGCACCTATCTCAGCGATCTGTCTATTTCGTTCATCCATAGTTGCCTGACTCCCCGTCGTGTAGATAACTACGA
TACGGGAGGGCTTACCATCTGGCCCCAGTGCTGCAATGATACCGCGAGACCCACGCTCACCGGCTCCAGATTTAT
CAGCAATAAACCAGCCAGCCGGAAGGGCCGAGCGCAGAAGTGGTCCTGCAACTTTATCCGCCTCCATCCAGTCTA
TTAATTGTTGCCGGGAAGCTAGAGTAAGTAGTTCGCCAGTTAATAGTTTGCGCAACGTTGTTGCCATTGCTACAG
GCATCGTGGTGTCACGCTCGTCGTTTGGTATGGCTTCATTCAGCTCCGGTTCCCAACGATCAAGGCGAGTTACAT
GATCCCCCATGTTGTGCAAAAAAGCGGTTAGCTCCTTCGGTCCTCCGATCGTTGTCAGAAGTAAGTTGGCCGCAG
TGTTATCACTCATGGTTATGGCAGCACTGCATAATTCTCTTACTGTCATGCCATCCGTAAGATGCTTTTCTGTGA
CTGGTGAGTACTCAACCAAGTCATTCTGAGAATAGTGTATGCGGCGACCGAGTTGCTCTTGCCCGGCGTCAACAC
GGGATAATACCGCGCCACATAGCAGAACTTTAAAAGTGCTCATCATTGGAAAACGTTCTTCGGGGCGAAAACTCT
CAAGGATCTTACCGCTGTTGAGATCCAGTTCGATGTAACCCACTCGTGCACCCAACTGATCTTCAGCATCTTTTA
CTTTCACCAGCGTTTCTGGGTGAGCAAAAACAGGAAGGCAAAATGCCGCAAAAAAGGGAATAAGGGCGACACGGA
AATGTTGAATACTCATACTCTTCCTTTTTCAATATTATTGAAGCATTTATCAGGGTTATTGTCTCATGAGCGGAT
ACATATTTGAATGTATTTAGAAAAATAAACAAATAGGGGTTCCGCGCACATTTCCCCGAAAAGTGCCAC
